# First-Line Chemotherapy Regimens for Unresectable Locally Advanced or Metastatic Biliary Tract Cancer: A Systematic Review and Bayesian Network Meta-Analysis

**DOI:** 10.1001/jamanetworkopen.2026.6849

**Published:** 2026-04-15

**Authors:** Abdulaziz Elemosho, Alex B. Blair, Meher Angez, Odysseas P. Chatzipanagiotou, Areesh Mevawalla, Qaidar Alizai, Kizuki Yuza, Timothy M. Pawlik

**Affiliations:** 1 Department of Surgery, The Ohio State University Wexner Medical Center and James Comprehensive Cancer Center, Columbus

## Abstract

**Importance:**

Biliary tract cancers (BTCs) are aggressive malignant neoplasms with a dismal prognosis. Recent advancements, including immunotherapy and targeted therapies, have expanded therapeutic options, yet head-to-head comparisons between regimens remain limited.

**Objective:**

To conduct a comprehensive network meta-analyses (NMA) to include latest advances in BTC therapy to guide clinical practice.

**Data Sources:**

PubMed, Embase, and Cochrane Central were searched for randomized clinical trials (RCT) from 2000 to 2024, with manual updates through August 2025 and conference abstracts and ClinicalTrials.gov screened.

**Study Selection:**

Phase 2 to 3 RCTs of unresectable, locally advanced, or metastatic BTC reporting progression-free survival (PFS) or overall survival (OS) were eligible.

**Data Extraction and Synthesis:**

The Preferred Reporting Items for Systematic Reviews and Meta-Analyses reporting guideline was followed. Two reviewers independently extracted data, resolving discrepancies with a third. Data were pooled with a fixed-effect model.

**Main Outcomes and Measures:**

Primary outcomes were PFS and OS; secondary outcomes included objective response rate (ORR) and grade 3 to 4 adverse events.

**Results:**

This systematic review and bayesian NMA synthesized data from 33 RCTs involving 7303 patients (median [IQR] age, 64 [63-65] years; 3704 [51.5%] male). Compared with gemcitabine plus cisplatin (GC), GC plus sintilimab plus anlotinib (hazard ratio [HR], 0.47; 95% CI, 0.28-0.80), GC plus S-1 (HR, 0.75; 95% CI, 0.59-0.97), and GC plus durvalumab (HR, 0.80; 95% CI, 0.66-0.97) were associated with highest PFS benefits. Capecitabine plus oxaliplatin (HR, 0.64; 95% CI, 0.44-0.92), GC plus durvalumab (HR, 0.71; 95% CI, 0.61-0.84), and gemcitabine plus oxaliplatin (GO) (HR, 0.78; 95% CI, 0.63-0.96) were associated with improved OS. ORR was highest with GC plus S-1 (odds ratio [OR], 4.13; 95% CI, 2.20-7.70), GC plus cediranib (OR, 3.20; 95% CI, 1.40-7.20), and GC plus durvalumab (OR, 1.60; 95% CI, 1.10-2.28) compared with GC. Toxicity profiles showed no significant hematological differences between regimens, but nonhematological risks varied. Overall, GC plus durvalumab, GC plus S-1, GO plus panitumumab, and GO plus cetuximab showed the greatest consistency in the surface under the cumulative ranking curve value for PFS, OS, ORR, and safety.

**Conclusions and relevance:**

This NMA establishes GC plus durvalumab and GC plus S-1 as potential leading first-line options for advanced BTC due to a consistent clear benefit in PFS, OS, ORR, and safety. This study also establishes that GC remains among the top regimens, as most other therapy lacked a clear superior benefit or were associated with worse PFS or OS compared with GC.

## Key Points

**Question:**

Among first-line regimens for unresectable locally advanced or metastatic biliary tract cancers, how do progression-free survival, overall survival, and objective response rate associated with different therapies compare?

**Findings:**

Among 33 randomized clinical trials involving 7303 patients, gemcitabine and cisplatin (GC) plus durvalumab, GC plus S-1, gemcitabine and oxaliplatin (GO) plus panitumumab, and GO plus cetuximab demonstrated the greatest consistency in the surface under the cumulative ranking curve value for progression-free survival, overall survival, and objective response rate, making these the top 5 regimens in terms of overall outcomes. These regimens also demonstrated a comparable risk of hematological toxic effects.

**Meaning:**

These findings suggest these 5 regimens were associated with the best overall treatment outcome as well as hematological safety in patients with locally advanced or metastatic biliary tract cancers.

## Teaser

This systematic review and bayesian network meta-analysis evaluates the latest advances in metastatic biliary tract cancer (BTC) therapy to guide clinical practice.

## Introduction

Biliary tract cancers (BTCs) include a diverse group of aggressive malignant neoplasms originating from the biliary epithelium, anatomically categorized into intrahepatic cholangiocarcinoma (iCCA), extrahepatic cholangiocarcinoma (eCCA, including perihilar and distal subtypes), and gallbladder carcinoma (GBC).^1,2,3,4,5,6^ Diagnosis often occurs at advanced stages in 60% to 70% of cases with curative resection feasible in only 20% to 30% of patients; in turn, many patients have a dismal prognosis with 5-year overall survival (OS) below 20% across subtypes and frequently under 10% for metastatic disease.^7,8^

For unresectable, locally advanced, or metastatic BTC, systemic therapy is the guideline-endorsed first-line approach.^9,10,11^ Monotherapy regimens (eg, fluorouracil or gemcitabine) produced only modest survival (median OS 6-8 months), motivating evaluation of combination strategies.^12,13^ Platinum-based doublet therapy such as gemcitabine plus oxaliplatin (GO) demonstrated improved outcomes in phase 2 studies, and phase 3 evidence subsequently established gemcitabine-based combinations as the standard.^13,14,15^ In ABC-02, gemcitabine plus cisplatin (GC) improved median OS compared with gemcitabine alone (11.7 vs 8.1 months; hazard ratio [HR], 0.64), which was supported by BT-22 in a Japanese cohort (11.2 vs 7.7 months; HR, 0.69).^13,15^

More recently, biomarker-driven therapies and immunotherapy have expanded therapeutic options.^16,17,18,19^ Actionable alterations such as fibroblast growth factor receptor (FGFR) fusions and isocitrate dehydrogenase 1 variants have enabled targeted agents (pemigatinib in 2020 and ivosidenib in 2021) in later lines.^16,17^ In the first-line setting, TOPAZ-1 (2022) demonstrated that adding durvalumab to GC improved median OS (12.8 vs 11.5 months), and KEYNOTE-966 (2023) reported longer median OS with pembrolizumab plus GC (12.7 vs 10.9 months).^18,19^

Despite these advances, direct head-to-head comparisons remain limited, and regimen selection often depends on patient and disease factors (eg, performance status, comorbidities, biomarkers).^20^ Prior network meta-analyses (NMAs) have attempted to clarify comparative effectiveness. A 2021 NMA (24 randomized clinical trials [RCTs]) evaluated chemotherapy plus targeted therapy relative to objective response rate (ORR), noting GO plus erlotinib had the highest ORR with potential toxicity trade-offs.^21^ A 2022 NMA (17 RCTs) suggested GC plus durvalumab was associated with favorable OS and progression-free survival (PFS), with GO and gemcitabine plus S-1 as alternatives.^22^ More recently, a 2023 umbrella review of 14 systematic reviews noted moderate to high evidence that fluoropyrimidine- and gemcitabine-based regimens improve OS and ORR but increase neutropenia risk, while emphasizing low certainty and methodological limitations.^23^

Given the rapidly evolving therapeutic landscape, the objective of the current study was to perform a systematic review and bayesian NMA of phase 2 to 3 randomized trials to compare first-line therapies for unresectable, locally advanced, or metastatic BTC. By jointly evaluating OS, PFS, ORR, and toxic effects, we aimed to define a therapeutic hierarchy to inform clinical decision-making and future trial design.

## Methods

### Search Strategy and Selection Criteria

This systematic review and NMA adhered to the Preferred Reporting Items for Systematic Reviews and Meta-Analyses (PRISMA) reporting guideline for NMAs. A comprehensive search was performed across PubMed, Cochrane Central Register of Controlled Trials, and Embase for RCTs published between January 1, 2000, and December 31, 2024. Manual searches for updates of included studies were conducted up to August 1, 2025, and integrated into the analysis. Relevant oncology conference websites, including the American Society of Clinical Oncology (ASCO), ASCO Gastrointestinal Cancers Symposium, European Society for Medical Oncology (ESMO), ESMO Gastrointestinal Cancer Congress, and ClinicalTrials.gov were reviewed. Reference lists of selected studies, meta-analyses, and reviews were also examined. Search terms encompassed key treatments, regimens, and terms related to BTC and first-line therapies, with the complete search strategy provided in the eMethods in Supplement 1.

Two investigators (A.E. and M.A.) independently conducted the literature search and study selection, resolving discrepancies through discussion or consultation with additional investigators (T.M.P. or A.B.B.). Eligible studies included phase 2 to 3 RCTs involving patients with previously untreated, unresectable, locally advanced, or metastatic BTC. Studies with borderline-resectable tumors were excluded to reduce bias from varying prognoses. Studies were required to report PFS or OS. No language restrictions were applied, and case reports, retrospective studies, single-group trials, reviews, meta-analyses, and editorials were excluded.

### Statistical Analysis

The primary outcomes of the systematic review and NMA were PFS and OS. Secondary outcomes included ORR and grade 3 to 4 adverse events, specifically anemia, neutropenia, thrombocytopenia, diarrhea, neuropathy, fatigue, nausea, and vomiting. Data were extracted from published reports, encompassing trial title, first author, publication year, study design, phase, location, patient demographics, study groups, treatment regimens, primary end points, tumor grade, HRs with 95% CIs, response rates, and adverse event counts. When CIs were unavailable but *P* values were reported, CIs were calculated using the method outlined by Altman et al.^24^ If HRs were not provided, Kaplan-Meier curves were digitally reconstructed to estimate HRs with 95% CIs using a validated method.^25^

Additional extracted variables included the number of patients with metastatic and locally advanced disease; median or mean age; ethnicity; Eastern Cooperative Oncology Group (ECOG) performance status; sex; primary tumor site; number and location of metastatic sites; prior treatments for nonmetastatic disease; and genomic alterations, if reported. For trials with multiple publications, the most recent and comprehensive report was selected. Full articles were prioritized, but conference abstracts with sufficient data were included (see eMethods in Supplement 1). In multigroup trials comparing different dosages of the same drug, a 2-group comparison was selected based on efficacy outcomes. For multigroup trials evaluating distinct regimens, all groups were included. Data extraction was independently performed by 2 investigators (A.E. and M.A.), with discrepancies resolved through discussion or consultation with a third investigator (T.M.P.).

NMA for all outcomes were conducted within a bayesian framework with network diagrams generated for each analysis. Treatment effect sizes were parameterized relative to GC as a clinically standard backbone to improve interpretability across heterogeneous trial populations. The similarity assumption was assessed by examining study designs and patient characteristics. Network inconsistency was evaluated using an unrelated mean-effects model and the node-splitting approach, with model fit compared using the deviance information criterion (DIC). Between-study variability in treatment effect sizes was quantified using the heterogeneity standard deviation τ. Publication bias for PFS and OS was assessed by analyzing HRs centered on comparison-specific effect sizes, with treatments ordered by median publication year and tested using the Egger test.

Effect sizes with 95% credible intervals (CrI) were estimated using Hamiltonian Monte Carlo sampling, employing 10 000 iterations, with 2000 discarded as burn-in, across 4 chains. Convergence was evaluated using the potential scale reduction factor. Noninformative normal priors were applied for treatment effect sizes, and half-normal priors for the between-study standard deviation τ. The DIC was used to select between random-effects and fixed-effects models with a fixed-effects model chosen when the DIC difference was 5 or less. Treatment rankings were determined using the surface under the cumulative ranking curve (SUCRA).^26^ Relative effect-size tables were used to present comparisons, and meta-regressions were conducted to assess the outcomes of study and patient characteristics associated with treatment effect sizes. A sensitivity analysis using a frequentist framework was performed for survival outcomes. For binary data, trials with 0-count cells were excluded, and a penalized likelihood NMA was used as a sensitivity analysis to address data sparseness.^27^

Posterior samples of log HRs were generated via Markov chain Monte Carlo; treatment effect sizes were expressed as HRs with posterior medians. To support probabilistic ranking, for each posterior draw, all regimens were ranked by HR and (1) the posterior probability of being best (Pr[rank = 1]) and (2) the posterior probability of being among the top 3 (Pr[rank ≤3]) were calculated. Pairwise posterior superiority probabilities between regimens were visualized as heatmaps.

The risk of bias was evaluated using the Cochrane Risk of Bias 2 (RoB2) tool with studies classified as low, some concerns, or high risk of bias by 2 investigators (A.E. and M.A.). Discrepancies were resolved through consensus or consultation with a third investigator (T.M.P.). The quality of evidence was assessed using the Grading of Recommendations Assessment, Development, and Evaluation approach, considering risk of bias, inconsistency, indirectness, imprecision, and other factors such as publication bias, large effect sizes, and plausible confounding. Evidence certainty was graded as very low, low, moderate, or high.^28^ All statistical analyses were performed using R Studio version 4.5.0 (R Project for Statistical Computing). Statistical significance was set at a 2-tailed *P* < .05. Eligible trials were analyzed collectively to reflect global trends, with sensitivity analyses conducted for specific populations (eg, Asian patients with S-1 regimens and fluke-related BTC), phase 3 trials only, and trials with ECOG 0 to 1 status.

## Results

Of the 32 included trial records, 30 contributed PFS data and 29 contributed OS data. in addition, 30 trials contributed orr data and 25 contributed adverse-event data (eTable 1 in Supplement 1). These included 15 trials (46.9%) conducted in Asia, 12 (37.5%) in Europe, 4 (12.5%) that were multinational or global, and 1 (3.1%) conducted in the US. The most commonly studied treatment backbone was GC, evaluated in 18 trials (56.2%), followed by GO-containing regimens in 8 trials (25.0%), gemcitabine monotherapy in 6 trials (18.8%), gemcitabine plus S-1 in 4 trials (12.5%), and S-1 monotherapy in 2 trials (6.2%). Across the included trials, the mean (SD) sample size was 248.1 (268.3) participants and the median (IQR) sample size was 139 (88.8-300.0) participants.

All included studies were randomized phase 2 or 3 trials; accordingly, the overall evidentiary base was strengthened by randomized allocation, but it was not uniformly at low risk of bias because many trials were open-label. Using a RoB2 framework, the most plausible concerns were related to deviations from intended interventions and measurement of nonblinded outcomes, whereas the risk of bias for more objective end points such as overall survival was likely lower than for progression-based or response-based outcomes. At the network level, these judgments were supported by the appendix analyses: in the main connected networks, 33 PFS trials and 31 OS trials were retained; heterogeneity was low for PFS (τ approximately 0.14) and moderate for OS (τ approximately 0.20), with no important evidence of global or local inconsistency and no detectable small-study effects on Egger testing. Taken together, the certainty of evidence is best described as moderate to high for the principal survival outcomes, rather than uniformly very high, with greater caution warranted for more subjective secondary end points (eMethods in Supplement 1).

**Figure 1.**
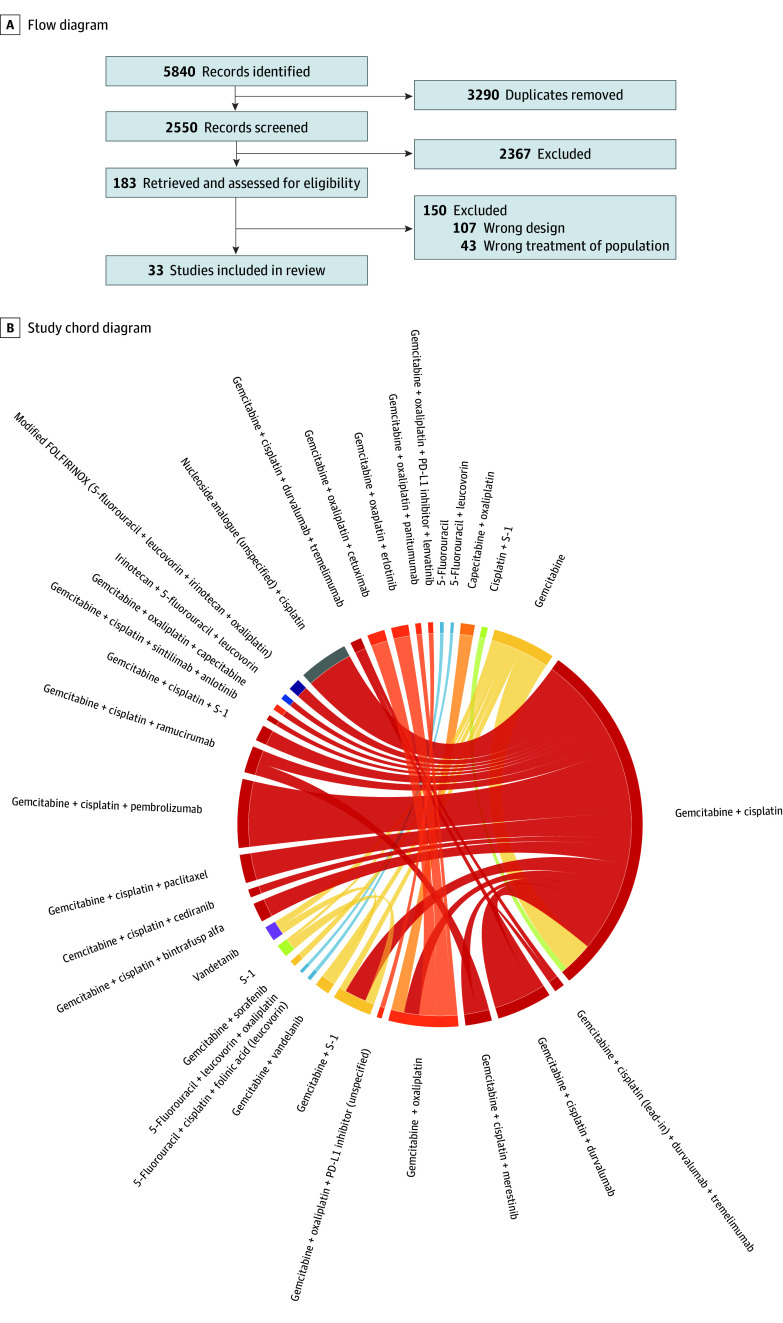
Flow and Chord Diagrams of the Included Studies PD-L1 indicates programmed death-ligand 1; +, plus.

The NMA for PFS included 30 trials^10,15,29,30,31,32,33,34,35,36,37,38,39,40,41,42,43,44,45,46,47,48,49,50,51,52,53,54,55,56,57,58^ for a total of 28 regimens and 7303 patients (Figure 1A and B and eFigures 1 and 2 in Supplement 1). A fixed-effects model was chosen justified by low heterogeneity (τ approximately 0.14; 95% CrI, 0.01-0.28) and a negligible difference in DIC (change in DIC, 0.1). Compared with GC, the quadruple regimen GC plus sintilimab plus anlotinib (HR, 0.47; 95% CI, 0.28-0.80), GC plus S-1 (HR, 0.75; 95% CI, 0.59-0.97), and GC plus durvalumab (HR, 0.80; 95% CI, 0.66-0.97) was associated with the highest PFS benefit. GC plus pembrolizumab (HR, 0.85; 95% CI, 0.76-0.96) also demonstrated higher PFS benefit compared with GC. The corresponding SUCRA scores and the HR of other regimens in this study are reported in eTable 2 and eFigures 3 and 4 in Supplement 1.

The NMA for OS included 29 trials^10,15,29,30,31,32,33,34,35,36,37,38,39,40,41,42,43,44,45,46,47,49,50,51,52,53,54,55,56,57^ for a total of 25 regimens and 7001 patients (eFigure 5 in Supplement 1). A random-effects model was chosen due to moderate heterogeneity (τ approximately 0.20; 95% CrI, 0.09-0.38) and a substantial DIC difference (change in DIC, 16.5). Compared with GC, capecitabine plus oxaliplatin (CAPOX; HR, 0.64; 95% CI, 0.44-0.92), GC plus durvalumab (HR, 0.71; 95% CI, 0.61-0.84), and GO (HR, 0.78; 95% CI, 0.63-0.96) were associated with the highest OS benefit. The corresponding SUCRA scores and the HR of other regimens in this study are reported in eTable 3 and eFigures 6 and 7 in Supplement 1.

In the analysis of response rate, 31 studies^10,15,29,30,31,32,33,34,35,36,37,38,39,40,41,42,43,44,45,46,47,49,51,52,53,54,55,56,57,58^ were included (27 regimens) for a total of 7160 patients and 1978 events (eFigure 8 in Supplement 1). Using a random-effects model (eAppendix in Supplement 1), GC plus S-1 (odds ratio [OR], 4.13; 95% CI, 2.2-7.70), GC plus cediranib (HR, 3.2; 95% CI, 1.4-7.2), GC plus durvalumab (HR, 1.6; 95% CI, 1.10-2.28), and GO plus erlotinib (HR, 2.48; 95% CI, 1.10-5.72) had higher likelihood to achieve an objective response compared with GC. The corresponding SUCRA scores and the ORs of other regimens are reported in eTable 4 and eFigures 9 and 10 in Supplement 1.

Subgroup analyses provided additional context for specific patient cohorts (eTables 5-10 and eFigures 11-28 in Supplement 1). In Asian populations, represented by 17 trials^10,15,29,33,34,35,36,37,38,39,42,43,48,50,57^ (12 regimens) (eFigures 20 and 21 in Supplement 1), GO plus cetuximab was associated with the highest PFS (HR, 0.61; 95% CI, 0.39-0.96) and OS (HR, 0.64; 95% CI, 0.43-0.92) benefits compared with GC. GC plus sintilimab plus anlotinib and GC plus S-1, respectively, were associated with a significant PFS benefit (sintilimab plus anlotinib: HR, 0.47; 95% CI, 0.28-0.80; GC plus S-1: HR, 0.75; 95% CI, 0.59-0.97) and with no clear OS benefit (sintilimab plus anlotinib: HR, 1.04; 95% CI, 0.54-2.01; GC plus S-1: HR, 0.81; 95% CI, 0.65-1.02) in the Asian subpopulation. Within the same subpopulation, GO and CAPOX, respectively, had a better OS benefit (GO: HR, 0.78; 95% CI, 0.64-0.96; CAPOX: HR, 0.64; 95% CI, 0.44-0.92) but no clear PFS benefit vs GC (eTables 10 and 11 in Supplement 1). In a subgroup analysis that included only patients with an ECOG of 0 to 1, GC plus durvalumab was associated with PFS (HR, 0.80; 95% CI, 0.66-0.97) or OS (HR, 0.71; 95% CI, 0.61-0.84) benefits vs GC alone.

In assessing data from phase 3 trials^29,34,36,38,40,43,47,49,51,54,57^ alone (eFigures 7 and 8 in Supplement 1), the results were consistent with the overall trend, with GC plus durvalumab having both better PFS (HR, 0.80; 95% CI, 0.66-0.97) and OS (HR, 0.71; 95% CI, 0.61-0.84). GC plus S-1 (HR, 0.75; 95% CI, 0.59-0.97) was associated with better PFS but not better OS. Of note, GC plus pembrolizumab was associated with a PFS benefit (HR, 0.85; 95% CI, 0.76-0.96) but a worse OS benefit compared with GC (HR, 1.20; 95% CI, 1.05-1.38) (eTables 8-9 in Supplement 1). Everything taken together, GC plus durvalumab, GC plus S-1, GO plus panitumumab, and GO plus cetuximab demonstrated the greatest consistency in the SUCRA value for OS, PFS, and ORR, making these the top 5 regimens in terms of overall outcomes.

### Posterior Ranking Probabilities

**Figure 2.**
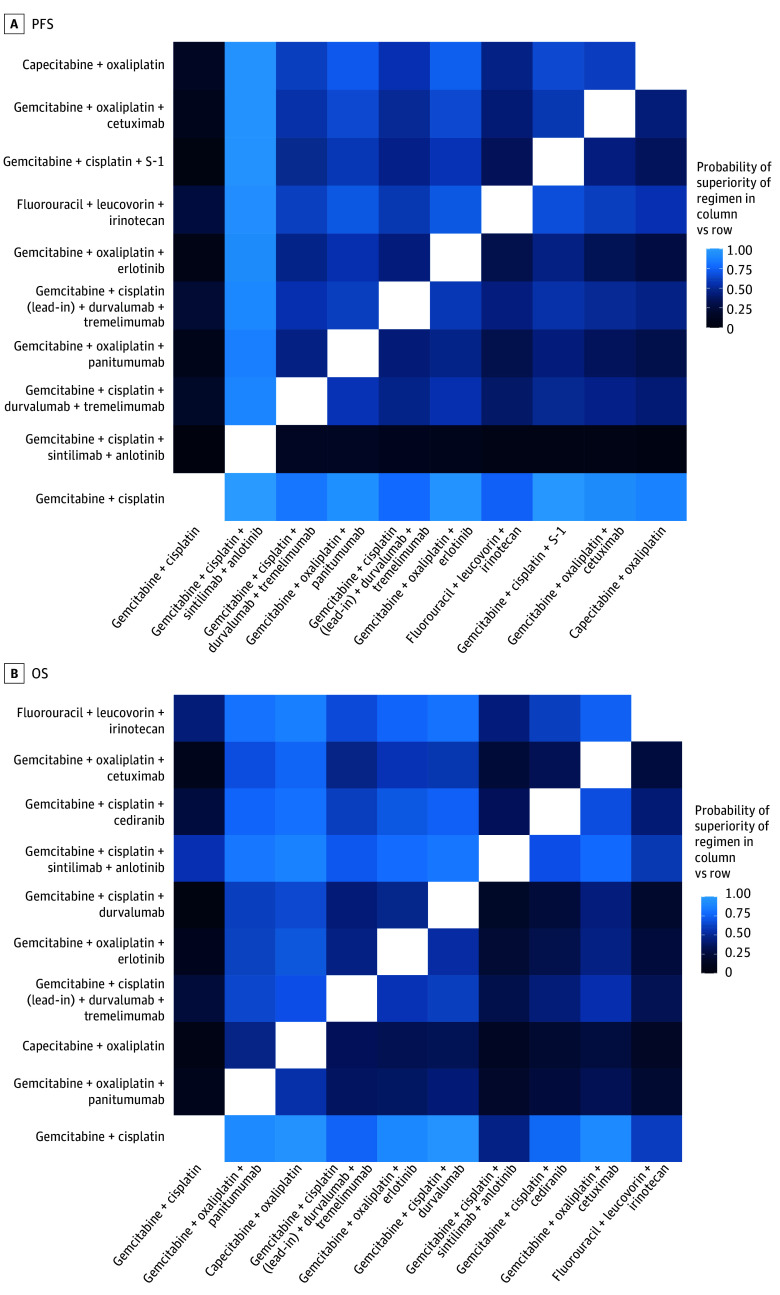
Heat Maps of the Top Regimens Associated With Progression-Free Survival (PFS) and Overall Survival (OS) Posterior Pairwise Superiority Probability Heat map showing, among the top 10 regimens by posterior ranking in the main network, the posterior probability that the regimen in the column is superior to the regimen in the row for A, PFS, defined as Pr(HR [column vs row]<1); and B, OS, defined as Pr(HR [column vs row]<1). + indicates plus.

GC plus durvalumab demonstrated less than 0.1% and 4.5% PFS probabilities of being best (PFS Pr[best]) and being top 3 (PFS Pr[top 3]), respectively, as well as 5.6% and 31.2% OS probabilities of being best (OS Pr[best]) and being top 3 (OS Pr[top 3]), respectively. GC plus S-1 had a PFS Pr(best) of 1.0% and Pr(top 3) of 20.2%, and OS Pr(best) of 2.5% and Pr(top 3) of 12.2%. GO plus panitumumab ranked highly for OS with a Pr(best) of 25.2% and a Pr(top 3) of 51.4% and also demonstrated meaningful PFS ranking (Pr[best], 8.0% and Pr[top 3], 39.4%). GO plus cetuximab had a PFS Pr(best) of 1.0% and Pr(top 3) of 14.5%, and OS Pr(best) of 3.5% and Pr(top 3) of 22.7% (Table). Figure 2A (PFS) and 2B (OS) depicts the posterior pairwise superiority probability heat map of the top regimens.

**Table.** Posterior Ranking Probabilities

Regimen (full labels)	Probability, %			
	PFS Pr(best)	PFS Pr(top 3)	OS Pr(best)	OS Pr(top 3)
Gemcitabine + cisplatin + durvalumab	<0.1	4.5	5.6	31.2
Gemcitabine + cisplatin + S-1	1.0	20.2	2.5	12.2
Gemcitabine + oxaliplatin + panitumumab	8.0	39.4	25.2	51.4
Gemcitabine + oxaliplatin + cetuximab	1.0	14.5	3.5	22.7
Gemcitabine + cisplatin + pembrolizumab	<0.1	1.0	<0.1	0.3
Gemcitabine (alone)	<0.1	<0.1	<0.1	<0.1
Gemcitabine + cisplatin + sintilimab + anlotinib	68.8	90.5	3.8	10.7
Capecitabine + oxaliplatin	0.6	9.5	23.7	58.0
Gemcitabine + oxaliplatin	<0.1	<0.1	<0.1	4.1

Abbreviations: +, plus; OS, overall survival; PFS, progression-free survival; Pr(best), probably of being the best treatment; Pr(top 3), probability of being in the top 3 treatments.

**Figure 3.**
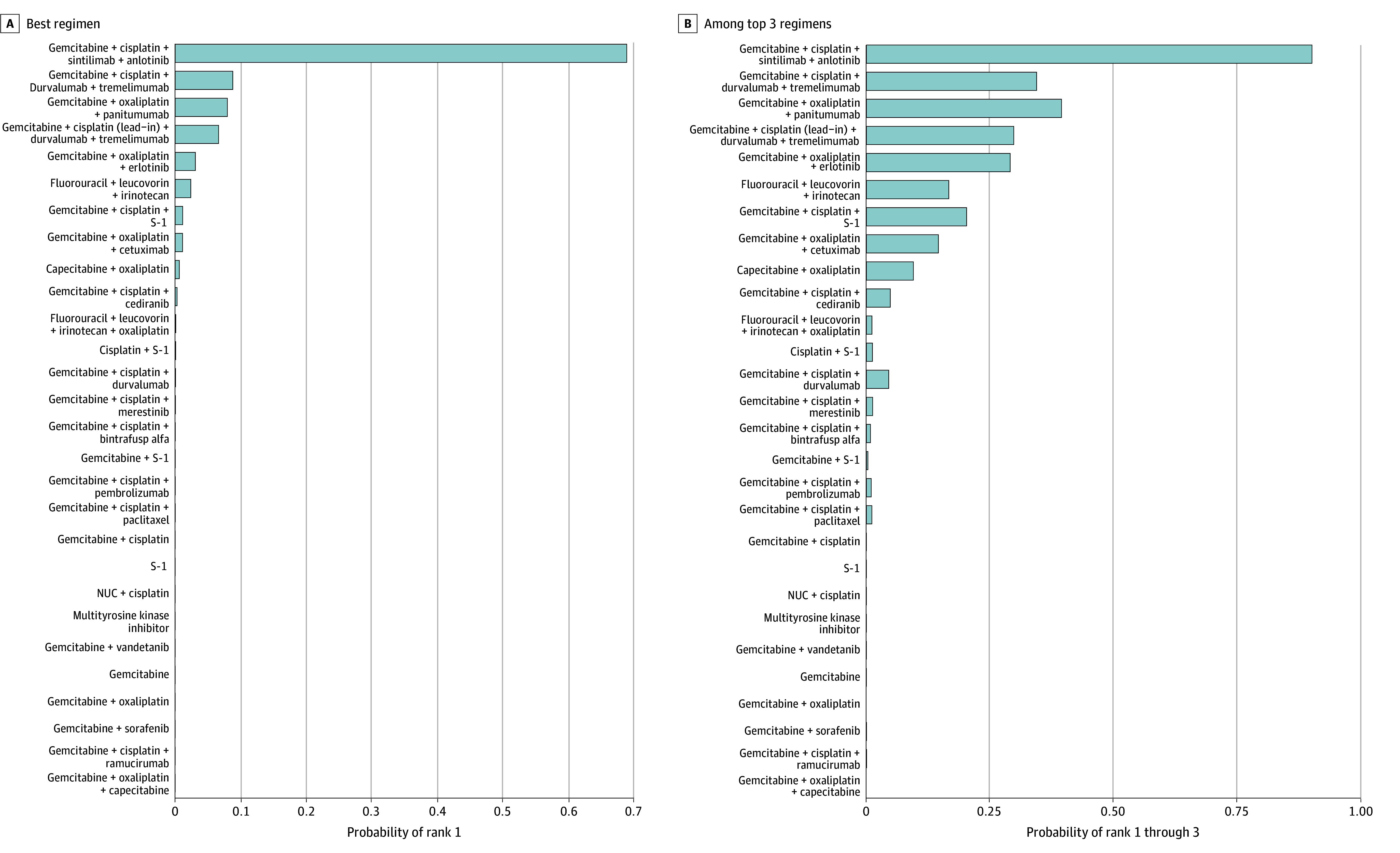
Bar Plots Showing Progression-Free Survival Posterior Probability of Being the Best and Among the Top 3 Regimens Bar plots showing, for each regimen in the main network, the posterior probability of being the best regimen (lowest hazard for progression-free survival; Pr[rank = 1]) and the probability of being among the top 3 regimens (Pr[rank ≤3]). Probabilities were estimated from posterior draws of hazard ratios from the bayesian network meta-analysis. + indicates plus.

**Figure 4.**
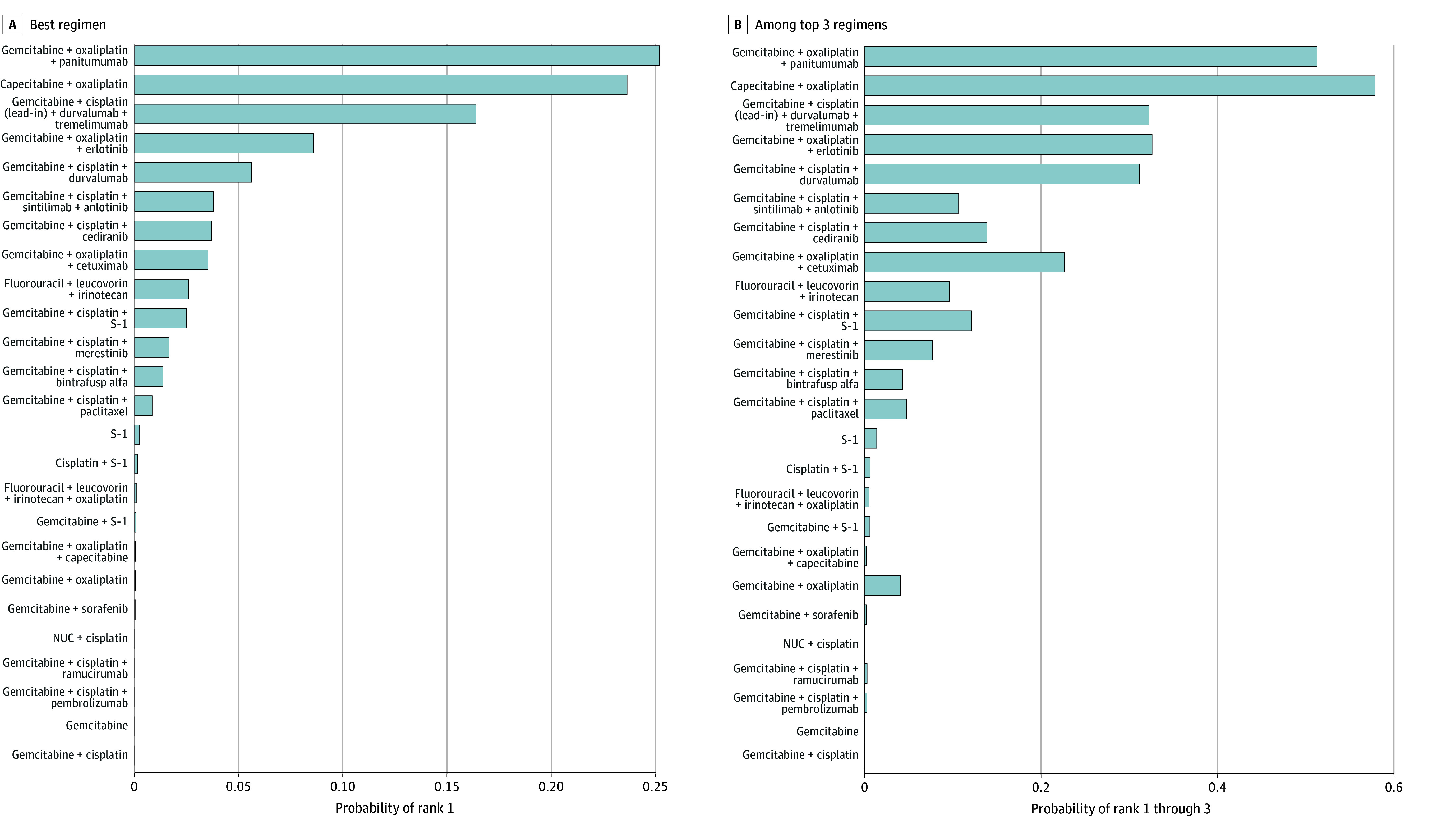
Bar Plots Showing Overall Survival Posterior Probability of Being the Best and Among the Top 3 Regimens Bar plots showing, for each regimen in the main network, the posterior probability of being the best regimen (lowest hazard for OS; Pr[rank = 1]) and the probability of being among the top 3 regimens (Pr[rank ≤3]). Probabilities were estimated from posterior draws of hazard ratios from the bayesian network meta-analysis.

Across the network, GC plus sintilimab plus anlotinib had the highest posterior support for PFS benefit (Pr[best], 68.8% and Pr[top 3], 90.5%), while CAPOX and GO plus panitumumab had the greatest posterior support for OS benefit (CAPOX: Pr[best], 23.7%; Pr[top 3], 58.0%; GO plus panitumumab: Pr[best], 25.2%; Pr[top 3], 51.4%). Figures 3 and 4 display the posterior probability of being best and among the top 3 regimens. The full heatmaps for all regimens for both PFS and OS are presented in eFigure 9 and 10 in Supplement 1.

Assessment of publication bias demonstrated no marked asymmetry around log-HR of 0 for either PFS or OS with nonsignificant intercepts, suggesting that the NMA estimates were unlikely to be distorted by selective publication of small or positive trials (eMethods in Supplement 1).

### Safety

In assessing adverse events, data related to the 5 most effective treatment regimens were reported. Of note, these 5 regimens had a comparable risk of hematological toxic effects (eTables 11-18 and eFigures 29-36 in Supplement 1). For nonhematological toxic effects, GC plus S-1 had a similar safety profile for diarrhea, nausea, vomiting, fatigue, and sensory neuropathy (eTable 6 in Supplement 1) compared with GC. GO plus cetuximab, and GO plus panitumumab both had higher rates of nausea and fatigue; however, they had a similar safety profile related to vomiting, diarrhea, and neuropathy vs GC. GC plus durvalumab was associated with a higher rate of fatigue compared with GC alone. In assessing overall outcomes, GC plus durvalumab, GC plus S-1, GO plus panitumumab, and GO plus cetuximab demonstrated the greatest consistency in the SUCRA value for OS, PFS, ORR, and safety. (eTables 11-18 and eFigures 29-36 in Supplement 1). eFigures 37 and 38 in Supplement 1 shows all regimens compared in a pairwise manner using a heatmap.

## Discussion

This systematic review and NMA constitutes an extensive assessment of first-line therapies for unresectable locally advanced or metastatic BTC, incorporating 30 trials related to PFS and 29 related to OS. The results demonstrate that triplet combinations were associated with better PFS or OS beyond standard doublet GC.^21,22,23^ GC plus durvalumab was consistently associated with better PFS (HR, 0.80; 95% CrI, 0.66-0.97) and OS (HR, 0.71; 95% CI, 0.61-0.84) compared with GC. Data in the current NMA reinforced the findings of the phase 3 TOPAZ-1 trial that GC plus durvalumab was a more effective therapeutic combination for patients with advanced BTC compared with GC alone.^18^ The TOPAZ-1 trial reported an OS HR of 0.80 (95% CI, 0.66-0.97), and a median PFS HR of 0.75 (95% CI, 0.63-0.89). Updated data also indicated that twice as many patients were alive at 3 years, a clinically meaningful outcome in a disease with a historically dismal long-term prognosis.^59^ The phase 3 KEYNOTE-966 trial reinforced the role of immune checkpoint blockade by evaluating pembrolizumab in combination with GC.^19^ This regimen yielded an OS and PFS benefit compared with GC alone, although ORRs were similar in both study groups.^19^ The current NMA did not demonstrate an OS advantage for pembrolizumab in combination with GC, which may have been due to study heterogeneity. While both durvalumab and pembrolizumab are immune checkpoint inhibitors, durvalumab is a programmed death-ligand 1 inhibitor, while pembrolizumab is a programmed protein 1 inhibitor, which may also explain differences in the respective trial results.^18,19^ Regardless, findings from both TOPAZ-1 and KEYNOTE-966 reinforce immune checkpoint inhibitors as central to modern first-line therapy for BTC, although the magnitude of benefit remains modest in absolute terms.^18,19^

Similarly, GC plus S-1 was associated with improved PFS (HR, 0.75; 95% CI, 0.59-0.97) and ORR (OR, 4.13; 95% CI, 2.2-7.70), consistent with the findings of the KHBO1401-MITSUBA trial.^29^ Given the lack of an association with improved OS, GC plus S-1 utility may be more in delaying disease progression in chemotherapy-sensitive cohorts.^29^ Specifically, whereas median OS was 13.5 vs 12.6 months (HR, 0.79; 95% CI, 0.63-1.0; *P* = .046), median PFS was 7.4 vs 5.5 months (HR, 0.75; 95% CI, 0.58-0.97; *P* = .02). The efficacy of GC plus S-1 noted in the current NMA was consistent with the KHBO1401-MITSUBA trial, which was conducted in an Asian cohort in which BTC was predominantly intrahepatic cholangiocarcinoma related to *Opisthorchis viverrini*, *Clonorchis sinensis*, and chronic hepatitis B/C.^60,61^ The trial’s study population necessitates caution when extrapolating the results to other global cohorts of patients. In addition to the varied BTC etiologies among Asian cohorts, there are also key pharmacogenomic differences in S-1 metabolism to consider.^62^ Conversion of tegafur to 5-fluorouracil (5-FU) is mediated by cytochrome P450 2A6 (*CYP2A6)*, which has population-specific polymorphisms. Low-activity alleles such as *CYP2A6* *4*, common in Asian populations, slow 5-FU conversion and enhance tolerability, whereas high-activity alleles prevalent in non-Asian populations increase systemic 5-FU exposure and risk of toxic effects.^62^ These differences highlight the rationale to evaluate capecitabine, which bypasses CYP2A6 metabolism, as a more universally applicable fluoropyrimidine backbone in Western populations.^29^ The current pooled analysis confirmed MITSUBA’s safety profile, as we noted no differences in hematologic toxic effects (anemia, neutropenia, or thrombocytopenia) between GC plus S-1 and GC.^29^ In the NMA, integration of MITSUBA with other predominantly phase 2 trials widened CrIs, diluting statistical significance for OS (HR, 0.81; 95% CI, 0.65-1.02). Nonetheless, GC plus S-1 maintained highly consistent SUCRA rankings across efficacy end points, underscoring robust relative performance despite heterogeneity. Evidence quality, however, remains moderate due to reliance on a single phase 3 trial.

Targeted strategies with epidermal growth factor receptor inhibition have been evaluated in trials of GO combined with monoclonal antibodies.^30,31^ In the current NMA, GO plus cetuximab and GO plus panitumumab had a notable consistency in the cumulative ranking curve values for survival, response, and safety end points. These data were consistent with outcomes from the BINGO and Vecti-BIL trials, which yielded ORRs of approximately 23% to 33% among patients with *KRAS* wild-type variants, albeit without significant survival prolongation over chemotherapy alone.^30,31^ The phase 2 Vecti-BIL trial in patients with *KRAS* wild-type BTC demonstrated no clear benefit with panitumumab compared with placebo.^30^ Similarly, the phase 2 BINGO trial of cetuximab plus GO failed to demonstrate OS superiority or PFS advantage, although KRAS wild-type subgroups demonstrated better outcomes.^31^ Data in the current NMA suggested a selective survival benefit compared with GC.

### Limitations

The current study had several limitations. Dependence on aggregated data impeded adjustments for confounders such as molecular profiles or regional disparities. To this point, GO plus cetuximab conferred superior PFS and OS in Asian subgroups, echoing pan-Asian guidelines and TOPAZ-1 subgroup analyses that noted enhanced responses in Asian cohorts due to genetic variances due to higher FGFR alterations. Additionally, trial design heterogeneity and incomplete adverse event documentation may have engendered bias, although this limitation was mitigated through subgroup and sensitivity evaluations. Inability to compare immune-related adverse events of different regimens also precluded a comprehensive overview of safety profiles. The omission of proportional hazards verification and the provisional character of quality-of-life deductions constrained applicability, consistent with exploratory analyses in BTC trials; however, assessment of publication bias revealed no notable asymmetry, thereby reinforcing estimate reliability. Future research should emphasize biomarker-guided studies to personalize and tailor treatment selection, as well as sequencing optimization with second-line agents. Such personalized individual approaches to therapy may also mitigate toxic effects by avoiding agents with little or no potential therapeutic benefit.

## Conclusions

In conclusion, the current NMA examined 32 randomized trials that investigated a wide spectrum of regimens, ranging from doublet and triplet cytotoxic chemotherapy to combinations with immunotherapy and targeted agents. GC plus durvalumab is recommended for patients with advanced BTC who have ECOG performance status 0 to 1; GC plus S-1 is a viable treatment option, especially in Asian populations. GC therapy remains a standard option for individuals unsuitable for triplet therapy. Additional phase 3 data are needed relative to targeted approaches including GO plus cetuximab, as well as regimens that incorporate sintilimab and anlotinib. While several strategies demonstrated modest improvements in ORR, PFS, or OS, GC retained a generally favorable balance of efficacy and tolerability in many experimental groups. These findings highlight that escalation through more drugs did not necessarily translate to better outcomes. Instead, a rational and thoughtful approach to synergistic combination of agents is needed, with a focus on biologically informed strategies that optimize therapeutic efficacy while minimizing toxic effects.

## Notes

Supplement 1. eMethods. Heterogeneity and Consistency Assessment eFigure 1. Network Chord Plot of Included Trials eTable 1. Trial-Level Characteristics and End Point Availability eTable 2. PFS of All Trials eFigure 2. Network Graph of PFS of All Trials eFigure 3. PFS SUCRA eFigure 4. PFS Forest Plot eTable 3. HRs of All OS TRIALS eFigure 5. Network Graph OS of All Trials eFigure 6. OS SUCRA eFigure 7. OS Forest Plot eTable 4. Objective Response Rate (ORR) of All Trials eFigure 8. Network Graph for ORR eFigure 9. ORR SUCRA eFigure 10. OS Forest Plot of All Trials eTable 5. ECOG 0-1 Trials: PFS eFigure 11. PFS Network Graph of ECOG 0-1 Trials eFigure 12. ECOG 0-1 PFS SUCRA eFigure 13. PFS Forest Plot ECOG 0-1 Trials eTable 6. Overall Survival (ECOG 0-1) eFigure 14. OS Network Graph of ECOG 0-1 Trials eFigure 15. ECOG 0-1 OS SUCRA eFigure 16. ECOG 0-1 OS Forest Plot eTable 7. Phase 3 Trials PFS eFigure 17. PFS Network Graph of Phase 3 Trials eFigure 18. PFS SUCRA of Phase 3 Trials eFigure 19. PFS Forest Plot of HRs of Phase 3 Trials eTable 8. OS Phase 3 Trials eFigure 20. OS Network Graph of Phase 3 Trials eFigure 21. OS SUCRA of Phase 3 Trials eFigure 22. OS Forest Plot of Phase 3 Trials eTable 9. Asian Trials PFS eFigure 23. PFS Network Graph of Asian Trials eFigure 24. PFS SUCRA of Asian Trials eFigure 25. PFS Forest Plot of Asian Trials eTable 10. OS Asian Trials eFigure 26. OS Network Graph of Asian Trials eFigure 27. OS SUCRA of Asian Trials eFigure 28. OS Forest Plot of Asian Trials eTable 11. Anemia eFigure 29. Network Graph of Anemia eTable 12. Thrombocytopenia eFigure 30. Network Graph of Thrombocytopenia eTable 13. Neutropenia eFigure 31. Network Graph of Neutropenia eTable 14. Nausea eFigure 32. Network Graph of Nausea eTable 15. Vomiting eFigure 33. Network Graph of Vomiting eTable 16. Diarrhea eFigure 34. Network Graph of Diarrhea eTable 17. Fatigue eFigure 35. Network Graph of Fatigue eTable 18. Sensory Neuropathy eFigure 36. Network Graph of Sensory Neuropathy eFigure 37. Heat Map Showing the Posterior Probability (of All Regimen) That the Regimen in the Column is Superior to the Regimen in the Row for PFS, Defined as Pr[HR (Column vs Row) < 1] eFigure 38. Heat Map Showing the Posterior Probability (of All Regimen) That the Regimen in the Column is Superior to the Regimen in the Row for OS, Defined as Pr[HR (Column vs Row) < 1]

Supplement 2. Data Sharing Statement
