# Supplementary material for: First-Line Chemotherapy Regimens for Unresectable Locally Advanced or Metastatic Biliary Tract Cancer: A Systematic Review and Bayesian Network Meta-Analysis
Source: JAMA Netw Open. 2026 Apr 15;9(4):e266849. doi: 10.1001/jamanetworkopen.2026.6849 (PMC13084461; doi:10.1001/jamanetworkopen.2026.6849)
Supplement: Supplement 1. — eMethods. Heterogeneity and Consistency Assessment eFigure 1. Network Chord Plot of Included Trials eTable 1. Trial-Level Characteristics and End Point Availability eTable 2. PFS of All Trials eFigure 2. Network Graph of PFS of All Trials eFigure 3. PFS SUCRA eFigure 4. PFS Forest Plot eTable 3. HRs of All OS TRIALS eFigure 5. Network Graph OS of All Trials eFigure 6. OS SUCRA eFigure 7. OS Forest Plot eTable 4. Objective Response Rate (ORR) of All Trials eFigure 8. Network Graph for ORR eFigure 9. ORR SUCRA eFigure 10. OS Forest Plot of All Trials eTable 5. ECOG 0-1 Trials: PFS eFigure 11. PFS Network Graph of ECOG 0-1 Trials eFigure 12. ECOG 0-1 PFS SUCRA eFigure 13. PFS Forest Plot ECOG 0-1 Trials eTable 6. Overall Survival (ECOG 0-1) eFigure 14. OS Network Graph of ECOG 0-1 Trials eFigure 15. ECOG 0-1 OS SUCRA eFigure 16. ECOG 0-1 OS Forest Plot eTable 7. Phase 3 Trials PFS eFigure 17. PFS Network Graph of Phase 3 Trials eFigure 18. PFS SUCRA of Phase 3 Trials eFigure 19. PFS Forest Plot of HRs of Phase 3 Trials eTable 8. OS Phase 3 Trials eFigure 20. OS Network Graph of Phase 3 Trials eFigure 21. OS SUCRA of Phase 3 Trials eFigure 22. OS Forest Plot of Phase 3 Trials eTable 9. Asian Trials PFS eFigure 23. PFS Network Graph of Asian Trials eFigure 24. PFS SUCRA of Asian Trials eFigure 25. PFS Forest Plot of Asian Trials eTable 10. OS Asian Trials eFigure 26. OS Network Graph of Asian Trials eFigure 27. OS SUCRA of Asian Trials eFigure 28. OS Forest Plot of Asian Trials eTable 11. Anemia eFigure 29. Network Graph of Anemia eTable 12. Thrombocytopenia eFigure 30. Network Graph of Thrombocytopenia eTable 13. Neutropenia eFigure 31. Network Graph of Neutropenia eTable 14. Nausea eFigure 32. Network Graph of Nausea eTable 15. Vomiting eFigure 33. Network Graph of Vomiting eTable 16. Diarrhea eFigure 34. Network Graph of Diarrhea eTable 17. Fatigue eFigure 35. Network Graph of Fatigue eTable 18. Sensory Neuropathy eFigure 36. Network Graph of Sensory Neuropathy eFigur [file jamanetwopen-e266849-s001.pdf]

Supplemental Online Content

Elemosho A, Blair AB, Angez M, et al. First-line chemotherapy regimens for unresectable locally advanced or metastatic biliary tract cancer: a systematic review and bayesian network meta-analysis. *JAMA Netw Open*. 2026;9(4):e266849.
doi:10.1001/jamanetworkopen.2026.6849

eMethods. Heterogeneity and Consistency Assessment
eFigure 1. Network Chord Plot of Included Trials
eTable 1. Trial-Level Characteristics and End Point Availability
eTable 2. PFS of All Trials
eFigure 2. Network Graph of PFS of All Trials
eFigure 3. PFS SUCRA
eFigure 4. PFS Forest Plot
eTable 3. HRs of All OS TRIALS
eFigure 5. Network Graph OS of All Trials
eFigure 6. OS SUCRA
eFigure 7. OS Forest Plot
eTable 4. Objective Response Rate (ORR) of All Trials
eFigure 8 Network Graph for ORR
eFigure 9. ORR SUCRA
eFigure 10. OS Forest Plot of All Trials
eTable 5. ECOG 0-1 Trials: PFS
eFigure 11. PFS Network Graph of ECOG 0-1 Trials
eFigure 12. ECOG 0-1 PFS SUCRA
eFigure 13. PFS Forest Plot ECOG 0-1 Trials
eTable 6. Overall Survival (ECOG 0-1)
eFigure 14. OS Network Graph of ECOG 0-1 Trials
eFigure 15. ECOG 0-1 OS SUCRA
eFigure 16. ECOG 0-1 OS Forest Plot
eTable 7. Phase 3 Trials PFS
eFigure 17. PFS Network Graph of Phase 3 Trials
eFigure 18. PFS SUCRA of Phase 3 Trials
eFigure 19. PFS Forest Plot of HRs of Phase 3 Trials
eTable 8. OS Phase 3 Trials
eFigure 20. OS Network Graph of Phase 3 Trials
eFigure 21. OS SUCRA of Phase 3 Trials
eFigure 22. OS Forest Plot of Phase 3 Trials
eTable 9. Asian Trials PFS
eFigure 23. PFS Network Graph of Asian Trials
eFigure 24. PFS SUCRA of Asian Trials
eFigure 25 PFS Forest Plot of Asian Trials
eTable 10. OS Asian Trials
eFigure 26 OS Network Graph of Asian Trials
eFigure 27. OS SUCRA of Asian Trials
eFigure 28. OS Forest Plot of Asian Trials
eTable 11. Anemia
eFigure 29. Network Graph of Anemia
eTable 12. Thrombocytopenia
eFigure 30. Network Graph of Thrombocytopenia
eTable 13. Neutropenia
eFigure 31 Network Graph of Neutropenia
eTable 14. Nausea
eFigure 32. Network Graph of Nausea
eTable 15. Vomiting
eFigure 33 Network Graph of Vomiting
eTable 16. Diarrhea
eFigure 34 Network Graph of Diarrhea
eTable 17. Fatigue
eFigure 35 Network Graph of Fatigue
eTable 18. Sensory Neuropathy
eFigure 36. Network Graph of Sensory Neuropathy
eFigure 37. Heat Map Showing the Posterior Probability (of All Regimen) That the Regimen in the Column is Superior to the Regimen in the Row for PFS, Defined as Pr[HR (Column vs Row) < 1]
eFigure 38. Heat Map Showing the Posterior Probability (of All Regimen) That the Regimen in the Column is Superior to the Regimen in the Row for OS, Defined as Pr[HR (Column vs Row) < 1]

This supplemental material has been provided by the authors to give readers additional information about their work.

© 2026 Elemosho A et al. *JAMA Network Open*.

eMethods. Heterogeneity and Consistency Assessment
We conducted contrast-based network meta-analyses for progression-free survival (PFS) and overall survival (OS) using Bayesian and frequentist frameworks in R (v4.x) with the **gemtc** (v0.8) and **netmeta** packages.

1. **Connected-component pruning**
   - We built treatment-network graphs from two-arm contrasts and identified the largest connected component by treatment.
   - Only studies whose both arms lay within that component were retained for heterogeneity estimation: 33 of 36 PFS studies and 31 of 33 OS studies.
2. **Heterogeneity & model selection**
   - For each endpoint's main component, we fit random-effects and fixed-effect models of log-hazard ratios using **gemtc**.
   - Between-study heterogeneity (τ) was summarized as the posterior standard deviation with 95% credible intervals (CrI).
   - Model fit was compared via Deviance Information Criterion (DIC), computing ΔDIC = DIC_FE – DIC_RE; ΔDIC ≤ 5 indicates that fixed-effect modelling is adequate.
3. **Consistency assessment**
   - **Global consistency** was evaluated via design-by-treatment interaction decomposition in **netmeta**, partitioning the total Q statistic into within-design and between-design (inconsistency) components; $p > 0.05$ for the between-design Q indicates no evidence against consistency.
   - **Local consistency** was assessed by separating direct and indirect evidence (SIDE) within each closed loop via Z-tests; $p < 0.05$ denotes significant disagreement.

Endpoint	Total Trials	Trials in Main Component	τ (Mean, 95% CrI)	DIC_RE	DIC_FE	ΔDIC (FE–RE)	Preferred Model	Global Consistency (Q, df, p)
**PFS**	36	33	0.14 (0.01–0.28)	2.0	2.0	0.1	Fixed-effect	Q_between = 5.52, df = 2, p = 0.0634
**OS**	33	31	0.20 (0.09–0.38)	55.9	72.4	+16.5	Random-effects	Q_between = 2.28, df = 2, p = 0.3198

- **PFS**: Low heterogeneity (τ ≈ 0.14). Nearly identical DICs (ΔDIC = 0.1) support the fixed-effect model. Global consistency holds (p = 0.0634). All SIDE loop p-values exceeded 0.05 (range 0.0537–0.1916), indicating no local inconsistency.
- **OS**: Moderate heterogeneity (τ ≈ 0.20). DIC strongly favors random-effects (ΔDIC = +16.5). Global consistency holds (p = 0.3198), and local loop tests showed no significant discrepancies (all $p > 0.05$).

With heterogeneity and consistency assumptions confirmed, the analyses proceed under a fixed-effect framework for PFS and a random-effects framework for OS. The next step is to assess publication bias via comparison-adjusted funnel plots and Egger's tests.

Publication-Bias Assessment

Methods
We assessed small-study effects and publication bias using comparison-adjusted funnel plots and Egger's regression tests within the **netmeta** framework. For each endpoint's main connected network (PFS: 33 studies; OS: 31 studies), we:

1. Plotted comparison-adjusted funnel plots of study-specific log-HRs against their standard errors, ordering treatments by their random-effects pooled estimates and adjusting for multi-arm studies.
2. Applied Egger's linear regression test of funnel-plot asymmetry, regressing the treatment effect on its standard error and weighting by inverse variance; a non-significant intercept ($p > 0.05$) indicates no evidence of small-study effects.

Endpoint	Egger's t	df	p-value	Bias Estimate (SE)	τ^2 (residual het.)
**PFS**	0.35	31	0.7272	0.0638 (0.1812)	0.1850
**OS**	0.16	29	0.8747	0.0724 (0.4552)	1.0405

- **Funnel-plot symmetry:** Visual inspection of both PFS and OS comparison-adjusted funnels showed no marked asymmetry around log-HR = 0.
- **Egger's tests:** Non-significant intercepts for PFS (p = 0.727) and OS (p = 0.875) indicate no detectable small-study effects or publication bias.

Interpretation
The absence of funnel-plot asymmetry and non-significant Egger's tests suggest that our network meta-analysis estimates for both PFS and OS are unlikely to be distorted by selective publication of small or positive trials. We therefore proceed with confidence to report pooled treatment effects.

Regimen Keys

Abbreviation	Full Regimen Name
CAPOX	Capecitabine plus Oxaliplatin
CIS_S1	Cisplatin plus S-1
FOLFIRI	Fluorouracil plus Leucovorin plus Irinotecan
FOLFIRINOX	Fluorouracil plus Leucovorin plus Irinotecan plus Oxaliplatin
GEM	Gemcitabine
GEM_S1	Gemcitabine plus S-1
GEM_SORAF	Gemcitabine plus Sorafenib
GEM_VAN	Gemcitabine plus Vandetanib
GEMCIS_BINTRAFUSP	Gemcitabine plus Cisplatin plus Bintrafusp Alfa
GEMCIS_CEDIR	Gemcitabine plus Cisplatin plus Cediranib
GEMCIS_DURVA	Gemcitabine plus Cisplatin plus Durvalumab

© 2026 Elemosho A et al. *JAMA Network Open*.

GEMCIS_DURVA_TREME	Gemcitabine plus Cisplatin plus Durvalumab plus Tremelimumab
GEMCIS_Lead_in_DURVA_TREME	Gemcitabine plus Cisplatin (Lead-in) plus Durvalumab plus Tremelimumab
GEMCIS_MERES	Gemcitabine plus Cisplatin plus Merestinib
GEMCIS_PACLITAXEL	Gemcitabine plus Cisplatin plus Paclitaxel
GEMCIS_PEMBRO	Gemcitabine plus Cisplatin plus Pembrolizumab
GEMCIS_RAM	Gemcitabine plus Cisplatin plus Ramucirumab
GEMCIS_S1	Gemcitabine plus Cisplatin plus S-1
GEMCIS_SINT_ANLO	Gemcitabine plus Cisplatin plus Sintilimab plus Anlotinib
GEMOX	Gemcitabine plus Oxaliplatin
GEMOX_CAP	Gemcitabine plus Oxaliplatin plus Capecitabine
GEMOX_CETUX	Gemcitabine plus Oxaliplatin plus Cetuximab
GEMOX_ERLO	Gemcitabine plus Oxaliplatin plus Erlotinib
GEMOX_PANIT	Gemcitabine plus Oxaliplatin plus Panitumumab
MTKI	Multi-Tyrosine Kinase Inhibitor
NUC_CIS	Nuc plus Cisplatin
S1	S-1
VAN	Vandetanib
XELOX	Capecitabine plus Oxaliplatin

*Notes: This key provides the full regimen names corresponding to abbreviations used in the network meta-analysis datasets. Abbreviations are expanded based on standard oncology terminology.

© 2026 Elemosho A et al. *JAMA Network Open*.

eTable 1. Trial-Level Characteristics and End Point Availability

Study	Continent	Phase	ECOG eligibility	Regimen/comparison category	PFS	OS	ORR	Safety
Ioka[1]	Asia	III	0–2	GemCis/GC-containing	Yes	Yes	Yes	Yes
Ducreaux[2]	Europe	II	0–2	5-FU/cisplatin-based	Yes	Yes	Yes	Yes
Chen 2015[3]	Asia	II	0–1	GEMOX-containing	Yes	Yes	Yes	Yes
Do-Youn 2025[4]	Asia	II/III	NR	GemCis/GC-containing	Yes	Yes	Yes	Yes
Kang 2012[5]	Asia	II	0–2	GemCis/GC-containing	Yes	Yes	Yes	Yes
Lee 2012[6]	Asia	III	0–2	GEMOX-containing	Yes	Yes	Yes	Yes
Li H 2016[7]	Asia	NR in sheet	NR	GS-containing	Yes	Yes	Yes	No
Morizane 2013[8]	Asia	II	0–1	GS-containing	Yes	Yes	Yes	Yes
Morizane 2019[9]	Asia	III	0–1	GemCis/GC-containing	Yes	Yes	Yes	Yes
Oh 2022[10]	Asia	II	0–1	GemCis/GC-containing	Yes	Yes	Yes	Yes
Oh 2025 (TOPAZ-1 3-year OS update)[11]	Global	III	0–1	GemCis/GC-containing	No	Yes	Yes	Yes
Okusaka 2010[12]	Asia	II	0–1	GemCis/GC-containing	Yes	Yes	Yes	Yes
Phelip 2021[13]	Europe	II	0–1	GemCis/GC-containing	Yes	Yes	Yes	Yes
Sasaki 2013[14]	Asia	II	0–2	GS-containing	Yes	Yes	Yes	Yes
Sharma 2019[15]	Asia	III	0–2	GemCis/GC-containing	Yes	Yes	Yes	Yes
Valle 2015[16]	Europe	II	0–1	GemCis/GC-containing	Yes	Yes	Yes	Yes
Valle ramucirumab/merestinib[17]	Global	II	0–1	GemCis/GC-containing	Yes	Yes	Yes	Yes
Santoro[18]	Europe	II	0–2	Gemcitabine monotherapy	Yes	No	Yes	No
Valle 2010[19]	Europe	III	0–2	GemCis/GC-containing	Yes	Yes	Yes	Yes
Zhang 2024[20]	Asia	II	NR	GEMOX-containing	Yes	No	No	No
Ueno 2024[21]	Global	III	0–1	GemCis/GC-containing	Yes	Yes	Yes	No
Li 2023[22]	Asia	II	NR	GemCis/GC-containing	Yes	Yes	No	No
Shroff 2023[23]	US	III	NR	GemCis/GC-containing	Yes	Yes	Yes	No
Ettrich 2024[24]	Europe	II	0–1	GemCis/GC-containing	Yes	Yes	Yes	Yes
Markussen[25]	Europe	II	0–1	GemCis/GC-containing	Yes	Yes	Yes	Yes
Knox 2025[26]	Global	III	0–1	GemCis/GC-containing	Yes	Yes	Yes	Yes
Moehler 2014[27]	Europe	II	0–2	Gemcitabine monotherapy	Yes	Yes	Yes	Yes
Schinzari 2017[28]	Europe	II	0–2	5-FU monotherapy	Yes	Yes	Yes	Yes
Kim ST 2019[29]	Asia	III	0–2	GEMOX-containing	Yes	Yes	Yes	Yes
Malka 2014[30]	Europe	II	0–1	GEMOX-containing	Yes	Yes	Yes	Yes
Leone 2015[31]	Europe	II	NR	GEMOX-containing	Yes	Yes	Yes	Yes
Amin 2021[32]	Europe	II	0–2	Other	No	No	Yes	No

© 2026 Elemosho A et al. *JAMA Network Open*.

eFigure 1. Network Chord Plot of Included Trials

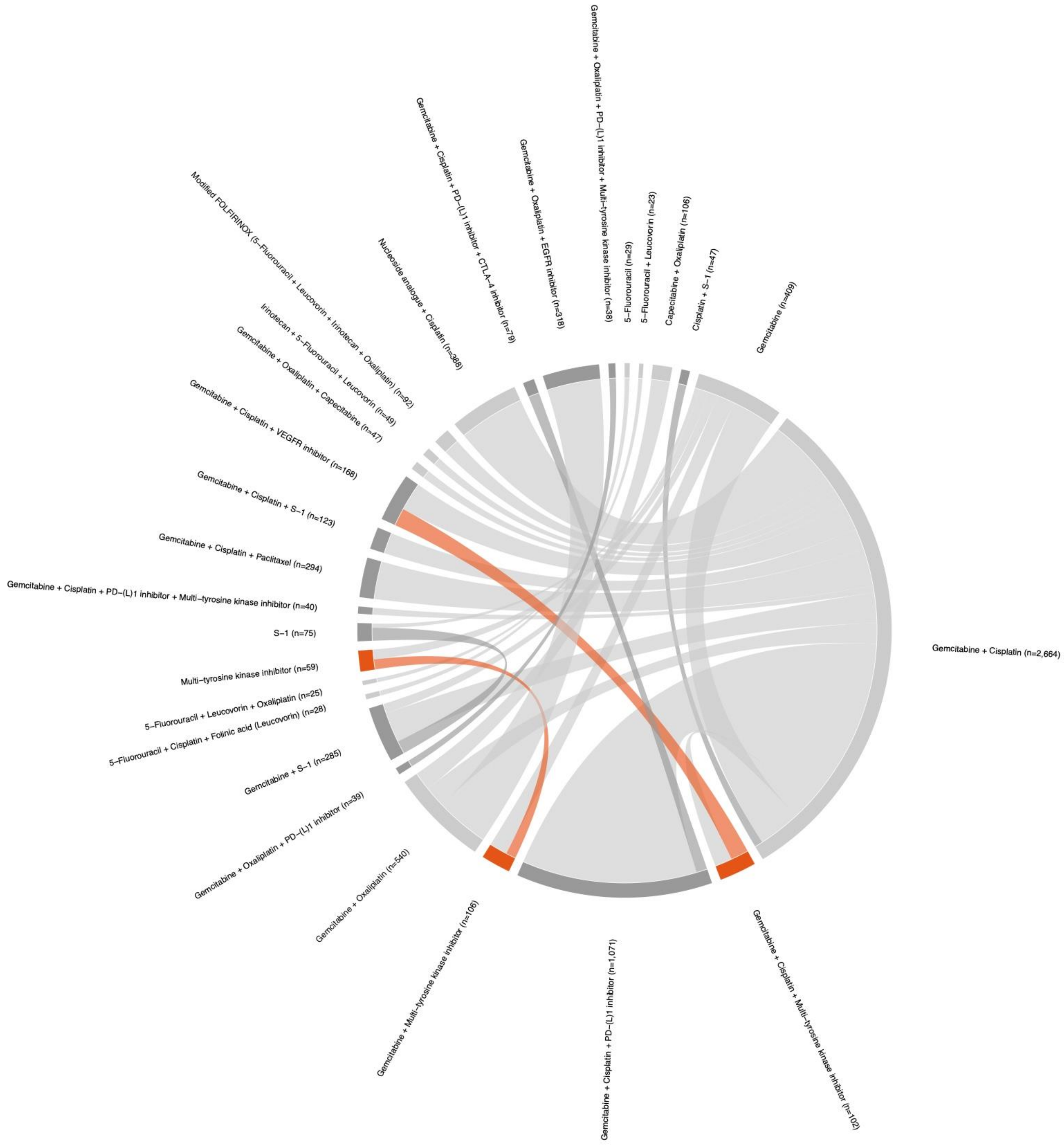

© 2026 Elemosho A et al. *JAMA Network Open*.

All (Global) BTC Population/Trials

eTable 1. PFS of All Trials

Regimen	HR with CI (95%)	SUCRA Rank
Gemcitabine plus Cisplatin plus Sintilimab plus Anlotinib	0.476 (0.284–0.799)	1
Gemcitabine plus Oxaliplatin plus Erlotinib	0.727 (0.511–1.035)	2
Gemcitabine plus Oxaliplatin plus Panitumumab	0.710 (0.435–1.159)	3
Gemcitabine plus Cisplatin plus S-1	0.752 (0.585–0.966)	4
Gemcitabine plus Cisplatin plus Durvalumab	0.800 (0.663–0.965)	5
Gemcitabine plus Oxaliplatin plus Cetuximab	0.785 (0.552–1.117)	6
Gemcitabine plus Cisplatin plus Durvalumab plus Tremelimumab	0.755 (0.405–1.408)	7
Gemcitabine plus Cisplatin (Lead-in) plus Durvalumab plus Tremelimumab	0.784 (0.422–1.456)	8
Capecitabine plus Oxaliplatin	0.819 (0.571–1.174)	9
Gemcitabine plus Cisplatin plus Pembrolizumab	0.847 (0.746–0.963)	10
Fluorouracil plus Leucovorin plus Irinotecan	0.847 (0.523–1.373)	11
Gemcitabine plus Cisplatin plus Cediranib	0.909 (0.639–1.294)	12
Gemcitabine plus Cisplatin plus Merestinib	0.909 (0.724–1.141)	13
Gemcitabine plus Oxaliplatin	0.909 (0.718–1.151)	14
Gemcitabine plus Cisplatin plus Paclitaxel	0.926 (0.730–1.174)	15
Gemcitabine plus S-1	0.928 (0.771–1.118)	16
Gemcitabine plus Cisplatin plus Bintrafusp Alfa	0.931 (0.748–1.421)	17
Gemcitabine plus Cisplatin plus Ramucirumab	1.124 (0.903–1.399)	18
Fluorouracil plus Leucovorin plus Irinotecan plus Oxaliplatin	1.176 (0.727–1.903)	19
Cisplatin plus S-1	1.176 (0.727–1.903)	20
Gemcitabine plus Oxaliplatin plus Capecitabine	1.387 (0.943–2.040)	21
Nuc plus Cisplatin	1.449 (1.176–1.786)	22
Gemcitabine	1.488 (1.257–1.762)	23
Gemcitabine plus Sorafenib	1.908 (1.180–3.086)	24
S-1	1.869 (1.284–2.721)	25
Multi-Tyrosine Kinase Inhibitor	1.933 (1.239–3.014)	26
Gemcitabine plus Vandetanib	1.933 (1.244–3.002)	27

*Notes: HR = Hazard Ratio; CI = Confidence Interval; SUCRA = Surface Under the Cumulative Ranking curve. SUCRA ranks are assigned based on descending order of adjusted P-scores, with higher P-scores indicating better efficacy and assigned ranks from 1 (best) to 27 (worst) among the 27 regimens. Only regimens with complete HR and CI data are included in the ranking.

eFigure 2. Network Graph of PFS of All Trials

© 2026 Elemosho A et al. *JAMA Network Open*.

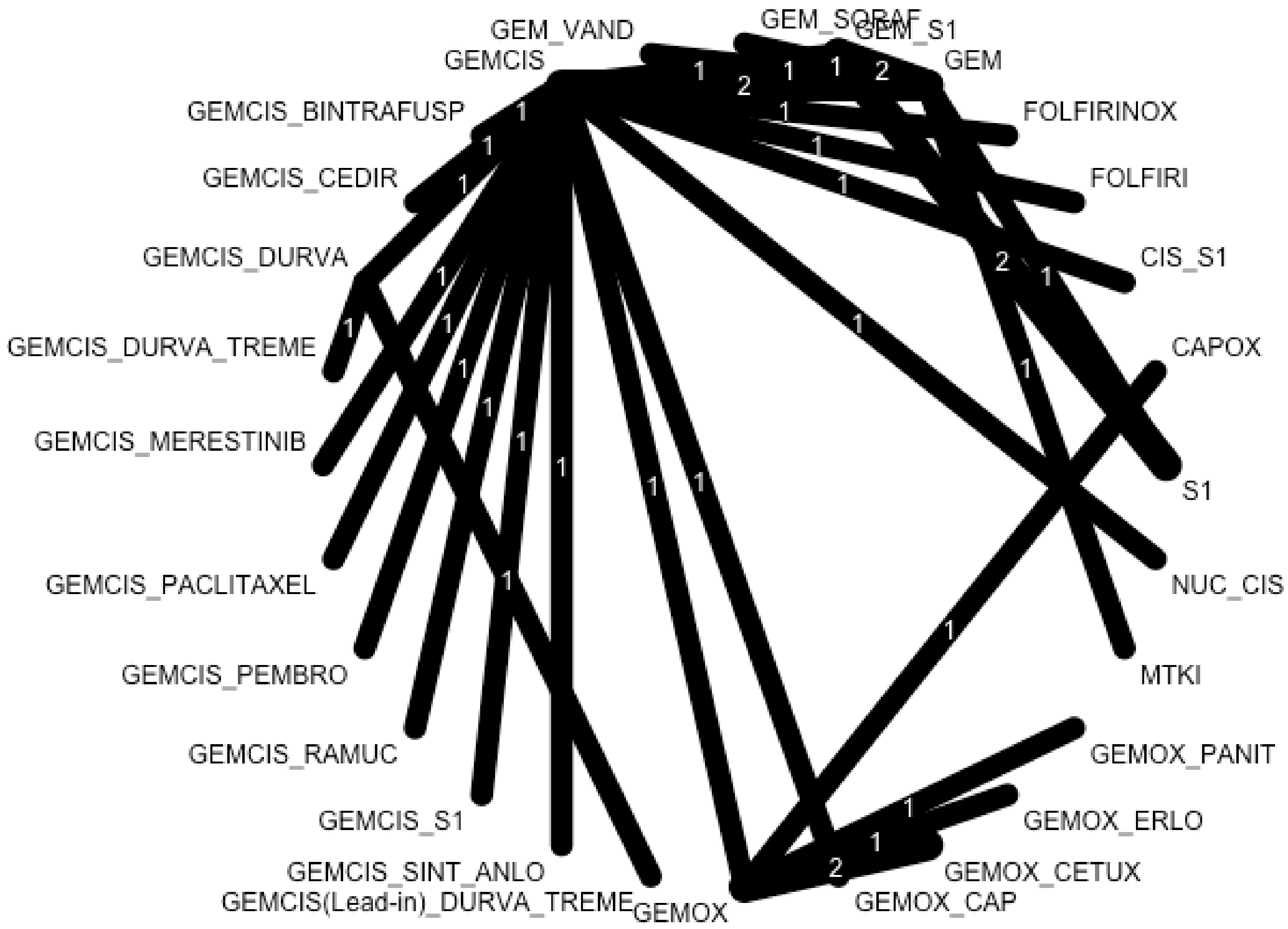

© 2026 Elemosho A et al. *JAMA Network Open*.

eFigure 3. PFS SUCRA

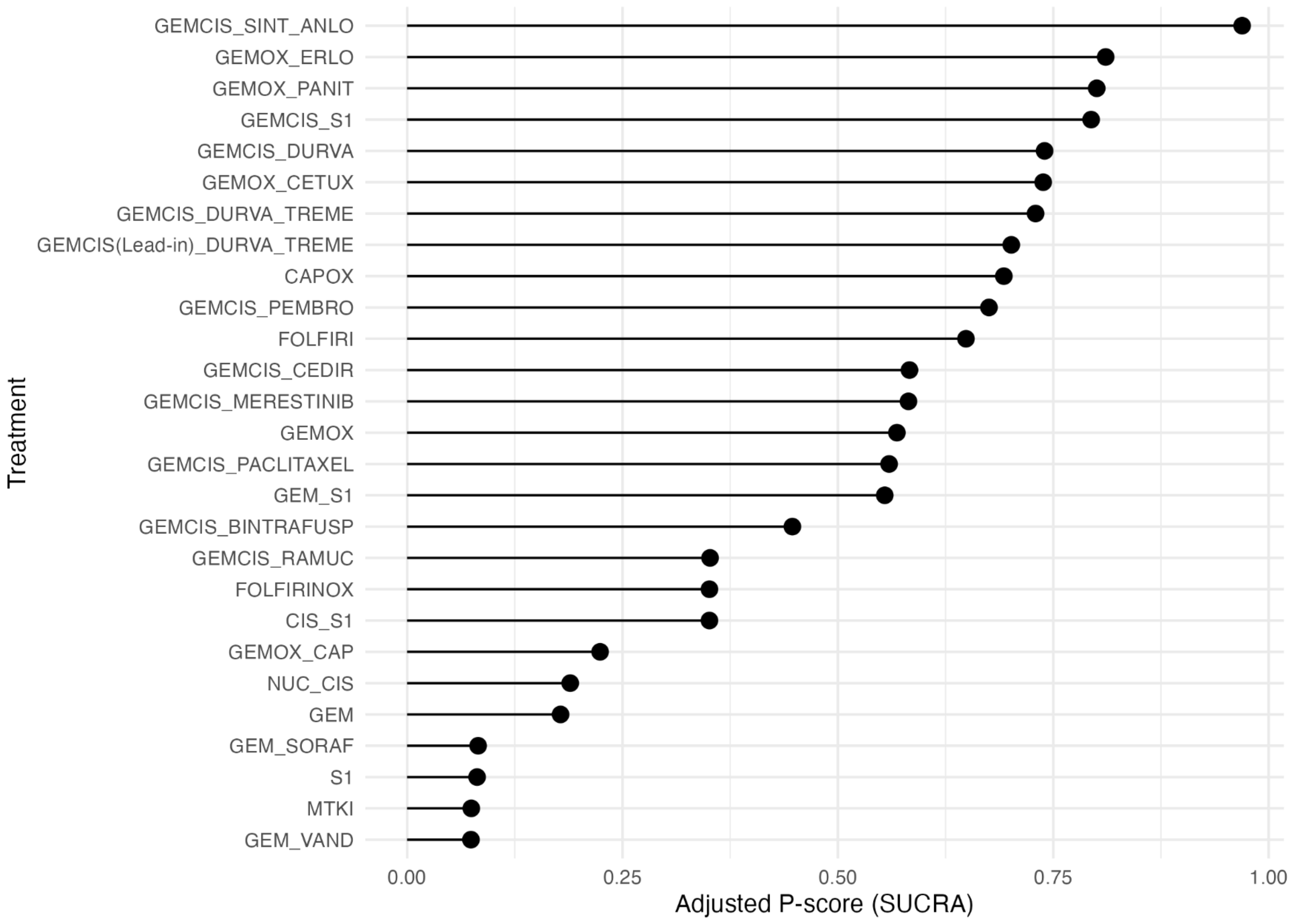

© 2026 Elemosho A et al. *JAMA Network Open*.

eFigure 4. PFS Forest Plot

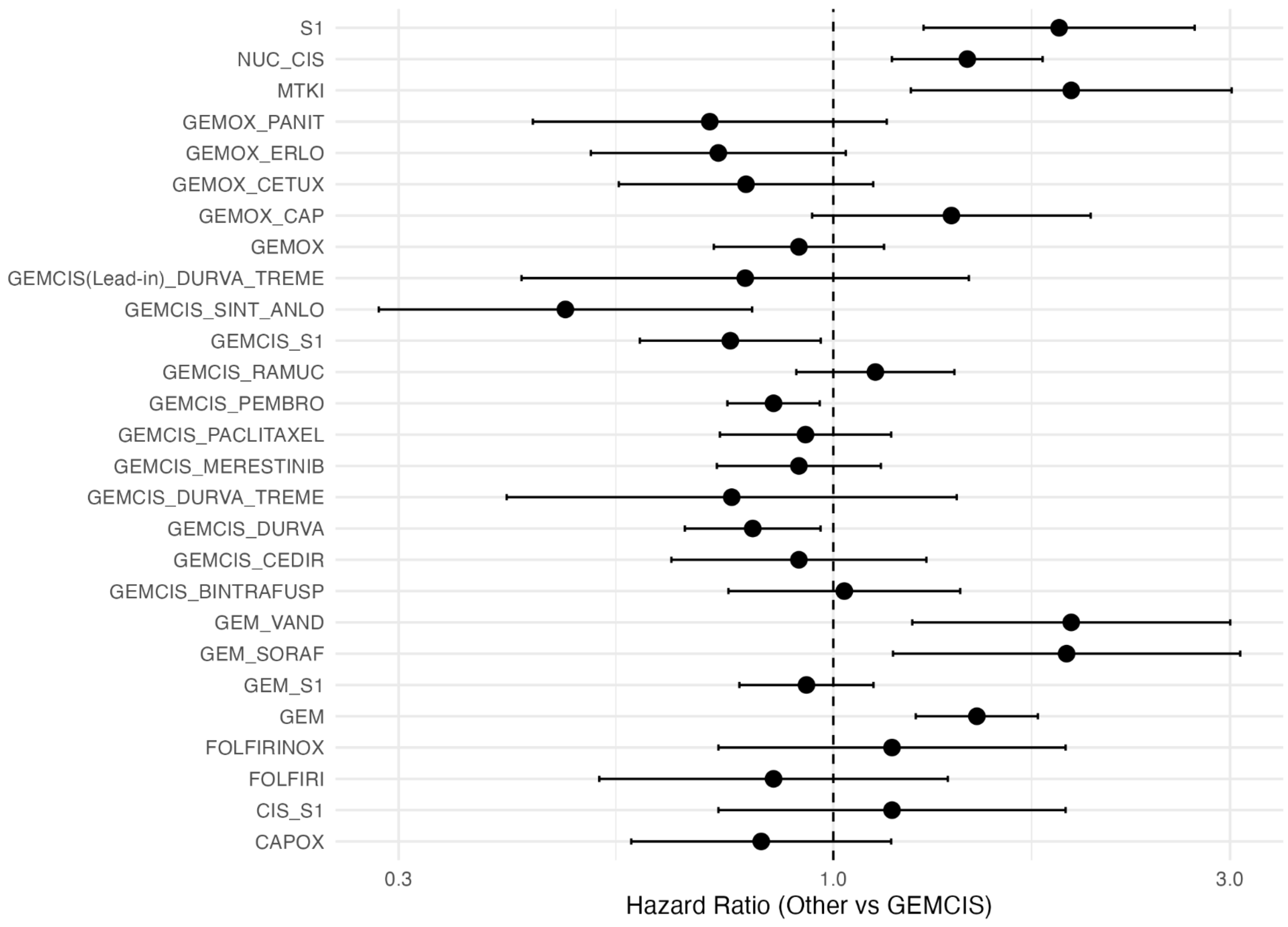

OS of All Trials

eTable 2. HRs of All OS TRIALS

Regimen	HR with CI (95%)	SUCRA Rank
Capecitabine plus Oxaliplatin	0.635 (0.441–0.916)	1
Gemcitabine plus Oxaliplatin plus Panitumumab	0.651 (0.397–1.068)	2
Gemcitabine plus Cisplatin plus Durvalumab	0.714 (0.607–0.840)	3
Gemcitabine plus Oxaliplatin plus Erlotinib	0.723 (0.503–1.040)	4
Gemcitabine plus Oxaliplatin plus Cetuximab	0.745 (0.546–1.015)	5
Gemcitabine plus Oxaliplatin	0.781 (0.635–0.962)	6
Gemcitabine plus Cisplatin plus S-1	0.813 (0.646–1.023)	7
Gemcitabine plus Cisplatin (Lead-in) plus Durvalumab plus Tremelimumab	0.776 (0.409–1.475)	8
Gemcitabine plus Cisplatin plus Cediranib	0.862 (0.584–1.272)	9
Gemcitabine plus Cisplatin plus Paclitaxel	0.909 (0.717–1.152)	10
Gemcitabine plus Cisplatin plus Merestinib	0.909 (0.645–1.281)	11
Fluorouracil plus Leucovorin plus Irinotecan	0.943 (0.589–1.512)	12
Gemcitabine plus S-1	0.981 (0.834–1.154)	13
Gemcitabine plus Cisplatin plus Sintilimab plus Anlotinib	1.042 (0.539–2.012)	14
S-1	1.020 (0.812–1.282)	15
Gemcitabine plus Cisplatin plus Bintrafusp Alfa	1.235 (0.634–2.405)	16
Gemcitabine plus Cisplatin plus Pembrolizumab	1.205 (1.049–1.384)	17
Fluorouracil plus Leucovorin plus Irinotecan plus Oxaliplatin	1.282 (0.907–1.813)	18
Gemcitabine plus Cisplatin plus Ramucirumab	1.333 (0.961–1.850)	19
Cisplatin plus S-1	1.389 (0.861–2.239)	20
Gemcitabine plus Oxaliplatin plus Capecitabine	1.368 (0.940–1.992)	21
Gemcitabine	1.482 (1.257–1.747)	22
Gemcitabine plus Sorafenib	1.785 (1.086–2.936)	23
Nuc plus Cisplatin	1.786 (1.271–2.510)	24

*Notes: HR = Hazard Ratio; CI = Confidence Interval; SUCRA = Surface Under the Cumulative Ranking curve. SUCRA ranks are assigned based on descending order of adjusted P-scores, with higher P-scores indicating better efficacy and assigned ranks from 1 (best) to 27 (worst) among the 27 regimens. Only regimens with complete HR and CI data are included in the ranking.

© 2026 Elemosho A et al. *JAMA Network Open*.

eFigure 5. Network Graph OS of All Trials

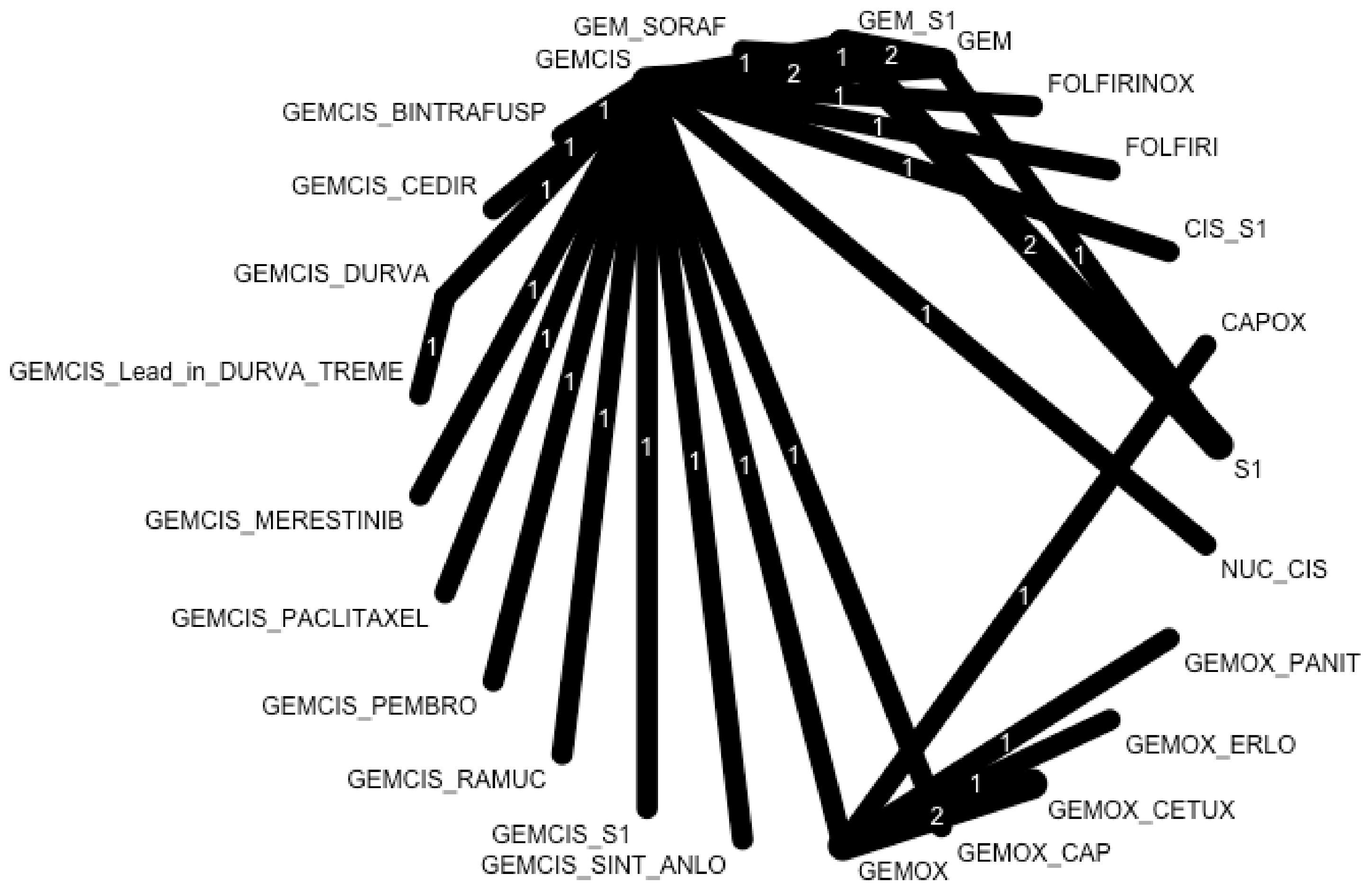

eFigure 6. OS SUCRA

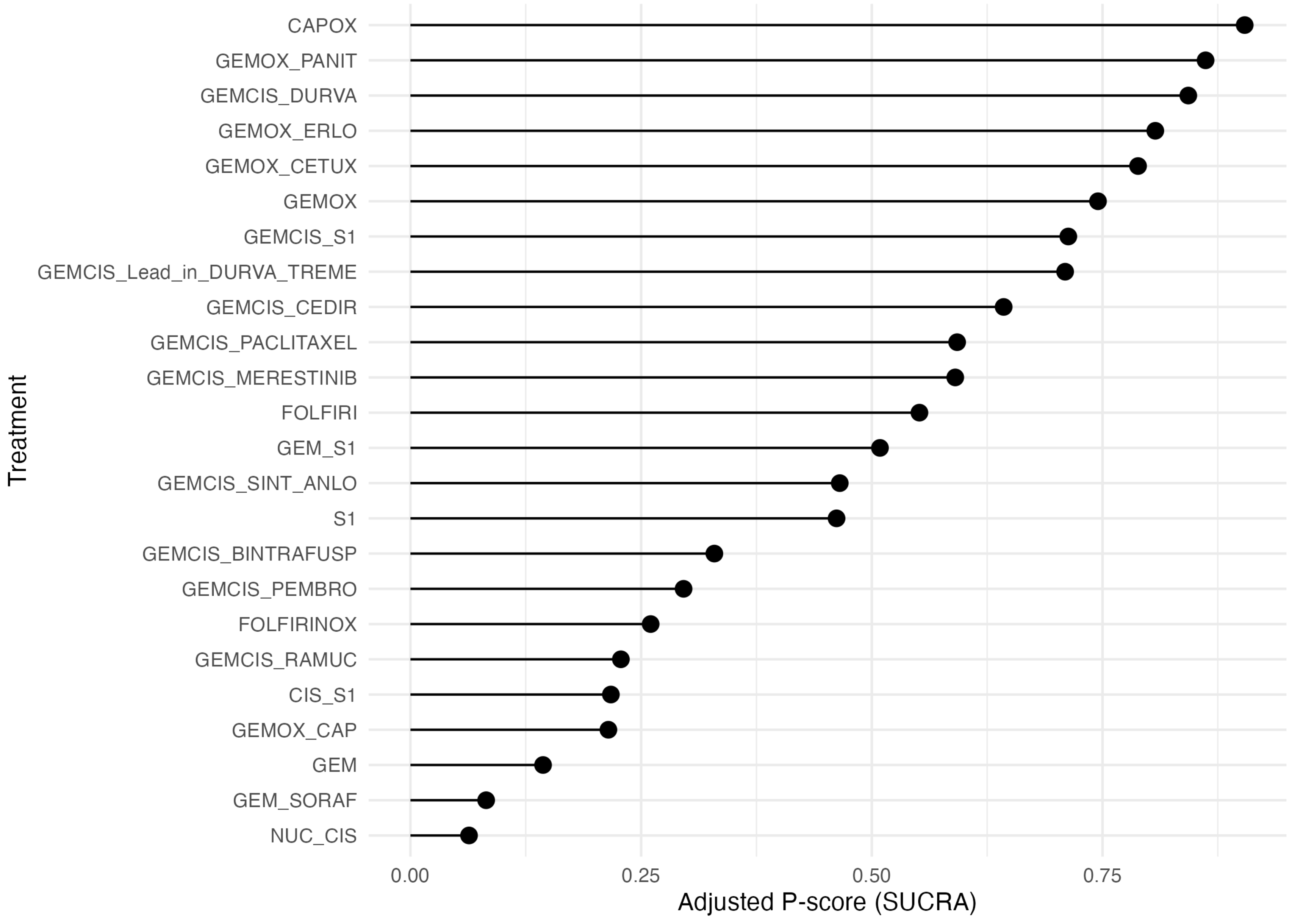

© 2026 Elemosho A et al. *JAMA Network Open*.

eFigure 7. OS Forest Plot

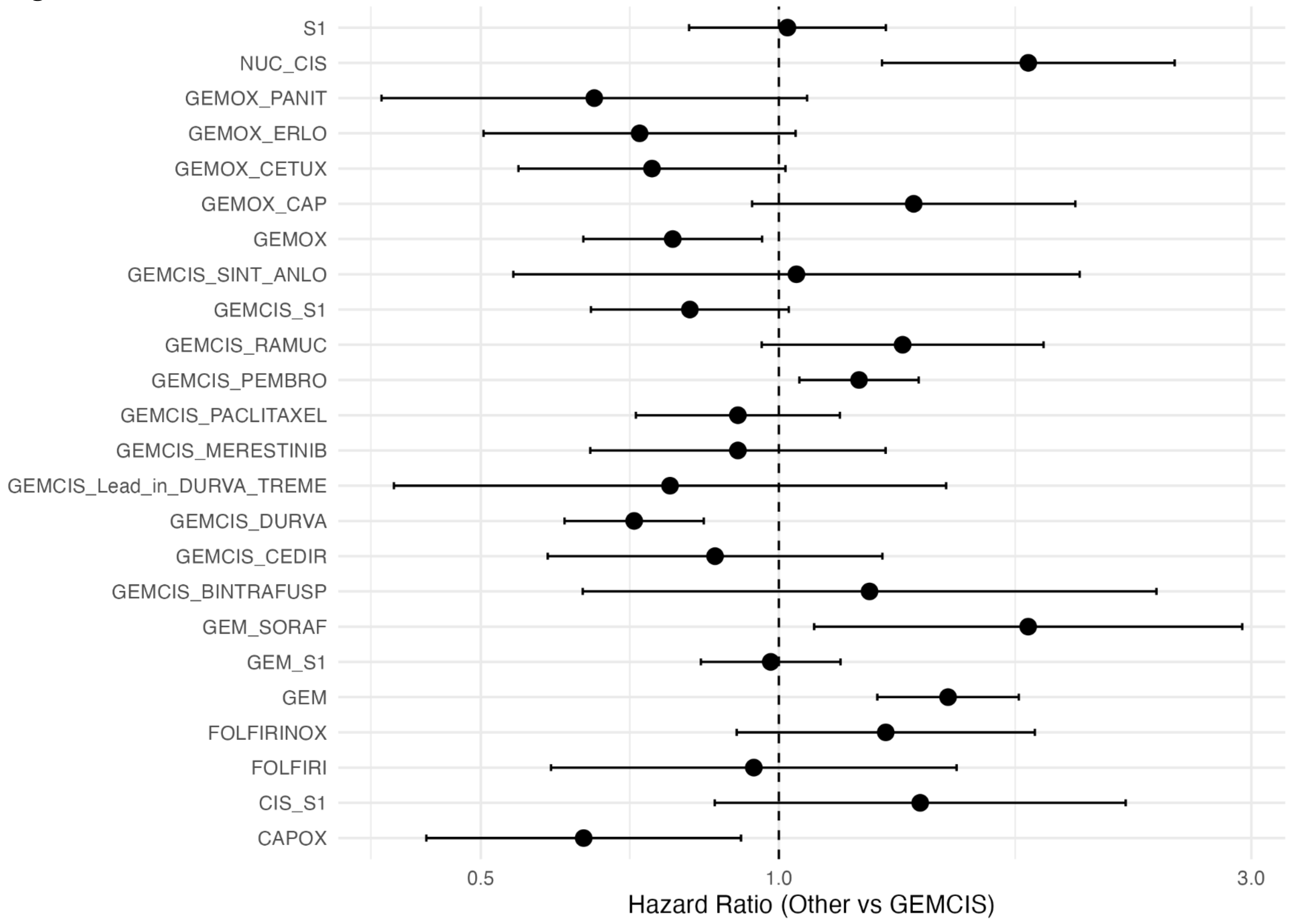

eTable 3. Objective Response Rate (ORR) of All Trials

Regimen	OR with CI	SUCRA Rank
Gemcitabine plus Cisplatin plus S-1	4.132 (2.233–7.645)	1
Gemcitabine plus Cisplatin plus Cediranib	3.214 (1.436–7.194)	2
Gemcitabine plus Oxaliplatin plus Erlotinib	2.480 (1.075–5.717)	3
Fluorouracil plus Leucovorin plus Irinotecan	2.400 (0.769–7.493)	4
Nuc plus Cisplatin	1.627 (1.096–2.416)	5
Gemcitabine plus Oxaliplatin plus Panitumumab	1.807 (0.561–5.818)	6
Gemcitabine plus Cisplatin plus Durvalumab	1.587 (1.104–2.280)	7
Gemcitabine plus Cisplatin plus Paclitaxel	1.532 (0.983–2.389)	8
Gemcitabine plus Cisplatin plus Durvalumab plus Tremelimumab	1.496 (0.577–3.876)	9
Gemcitabine plus Oxaliplatin plus Cetuximab	1.461 (0.649–3.286)	10
Fluorouracil plus Leucovorin plus Irinotecan plus Oxaliplatin	1.389 (0.691–2.792)	11
Cisplatin plus S-1	1.192 (0.452–3.140)	12
Gemcitabine plus Cisplatin plus Bintrafusp Alfa	1.183 (0.680–2.059)	13
Gemcitabine plus Oxaliplatin	1.104 (0.614–1.985)	14
Gemcitabine plus Oxaliplatin plus Capecitabine	1.051 (0.359–3.076)	15
Gemcitabine plus Cisplatin plus Pembrolizumab	0.999 (0.766–1.302)	16
Gemcitabine plus Cisplatin plus Ramucirumab	0.932 (0.519–1.672)	17
Gemcitabine plus Sorafenib	0.828 (0.228–3.004)	18
Gemcitabine plus S-1	0.898 (0.595–1.356)	19
Gemcitabine plus Vandetanib	0.792 (0.271–2.316)	20
Gemcitabine plus Cisplatin (Lead-in) plus Durvalumab plus Tremelimumab	0.635 (0.234–1.722)	21
Capecitabine plus Oxaliplatin	0.648 (0.265–1.587)	22
Gemcitabine	0.564 (0.378–0.842)	23
Gemcitabine plus Cisplatin plus Merestinib	0.503 (0.265–0.955)	24
S-1	0.269 (0.112–0.647)	25
Vandetanib	0.125 (0.024–0.651)	26

*Notes: HR = Hazard Ratio; CI = Confidence Interval; SUCRA = Surface Under the Cumulative Ranking curve. SUCRA ranks are assigned based on descending order of adjusted P-scores, with higher P-scores indicating better efficacy and assigned ranks from 1 (best) to 27 (worst) among the 27 regimens. Only regimens with complete HR and CI data are included in the ranking.

eFigure 8 Network Graph for ORR

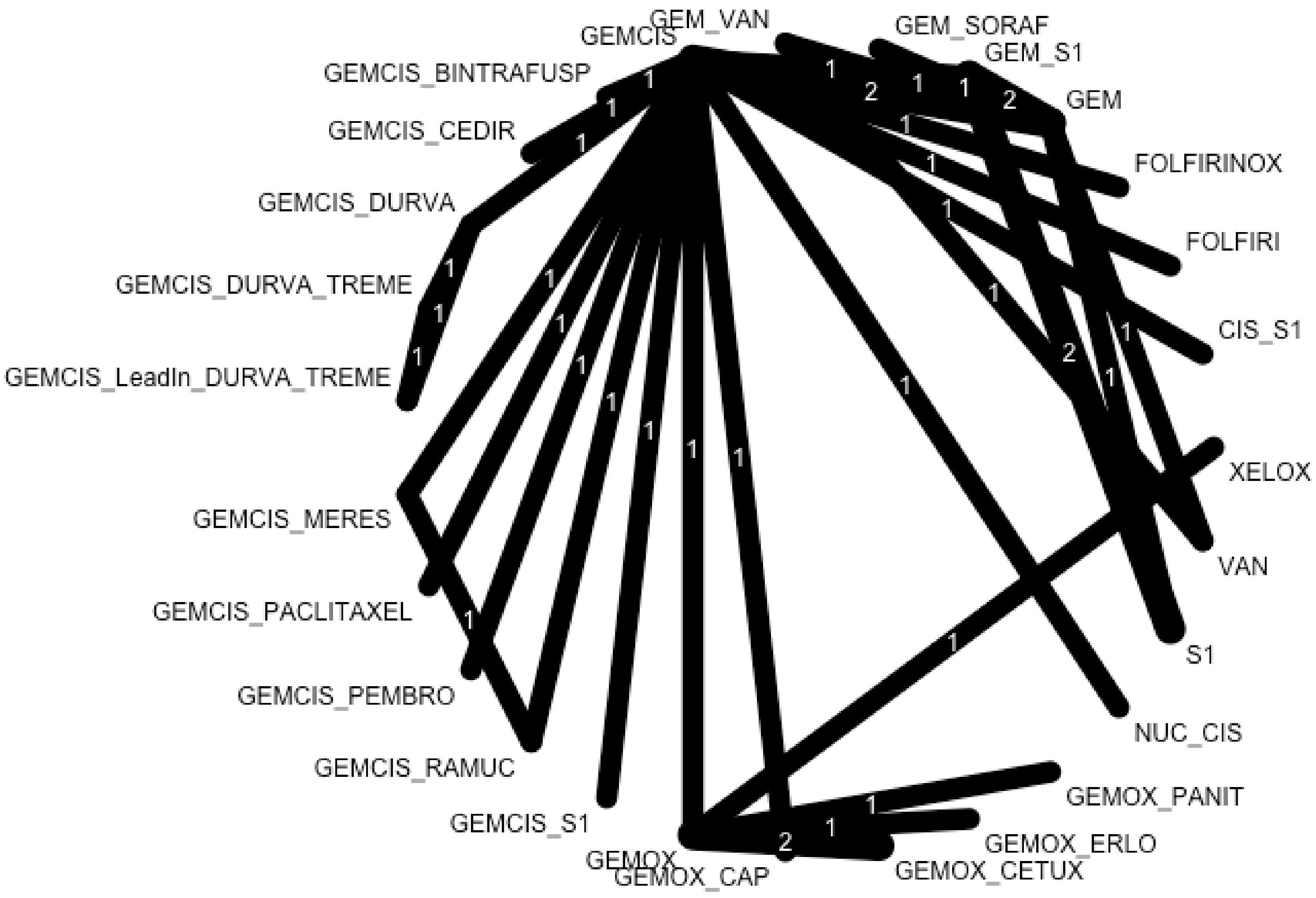

eFigure 9. ORR SUCRA

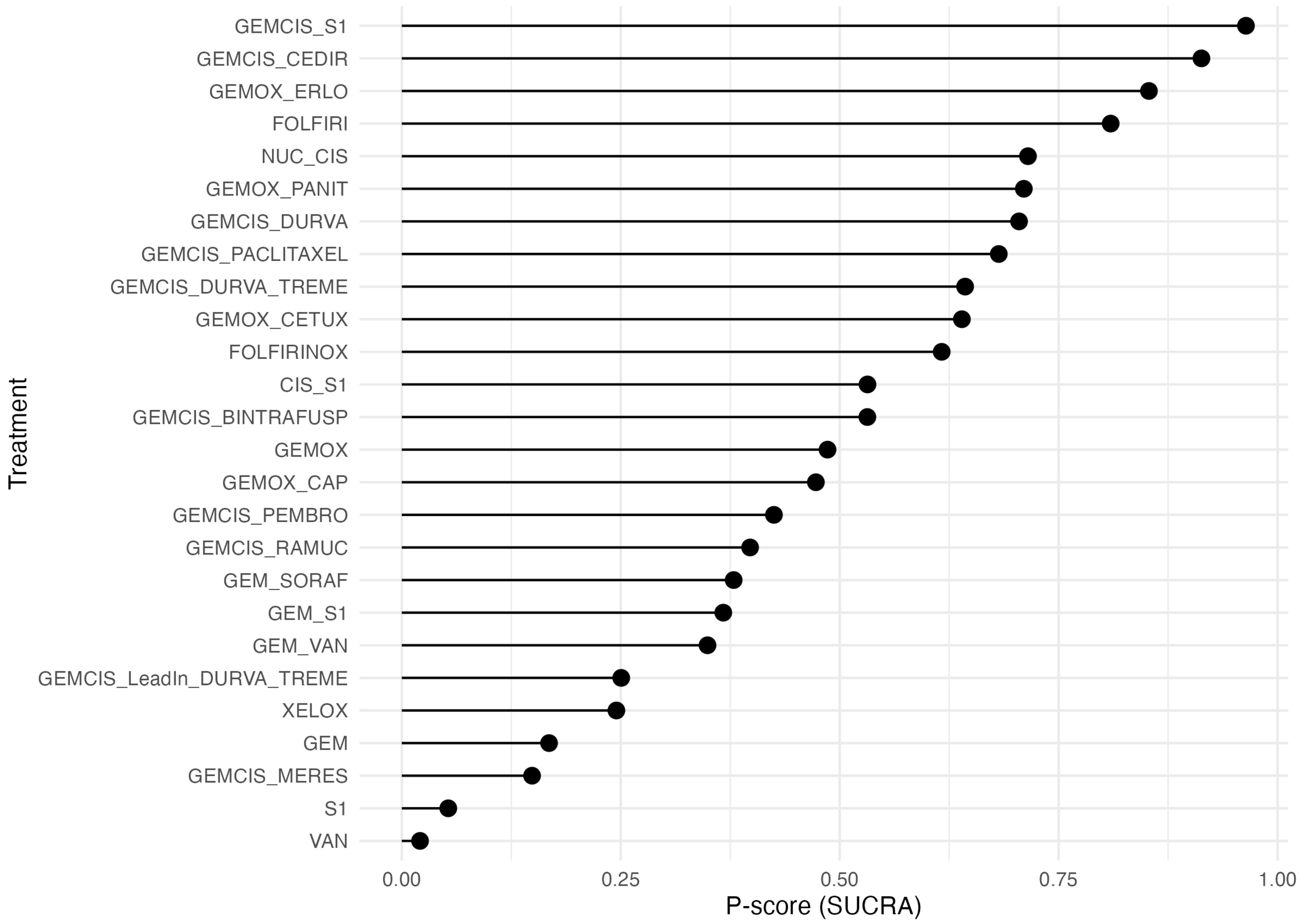

© 2026 Elemosho A et al. *JAMA Network Open*.

eFigure 10. OS Forest Plot of All Trials

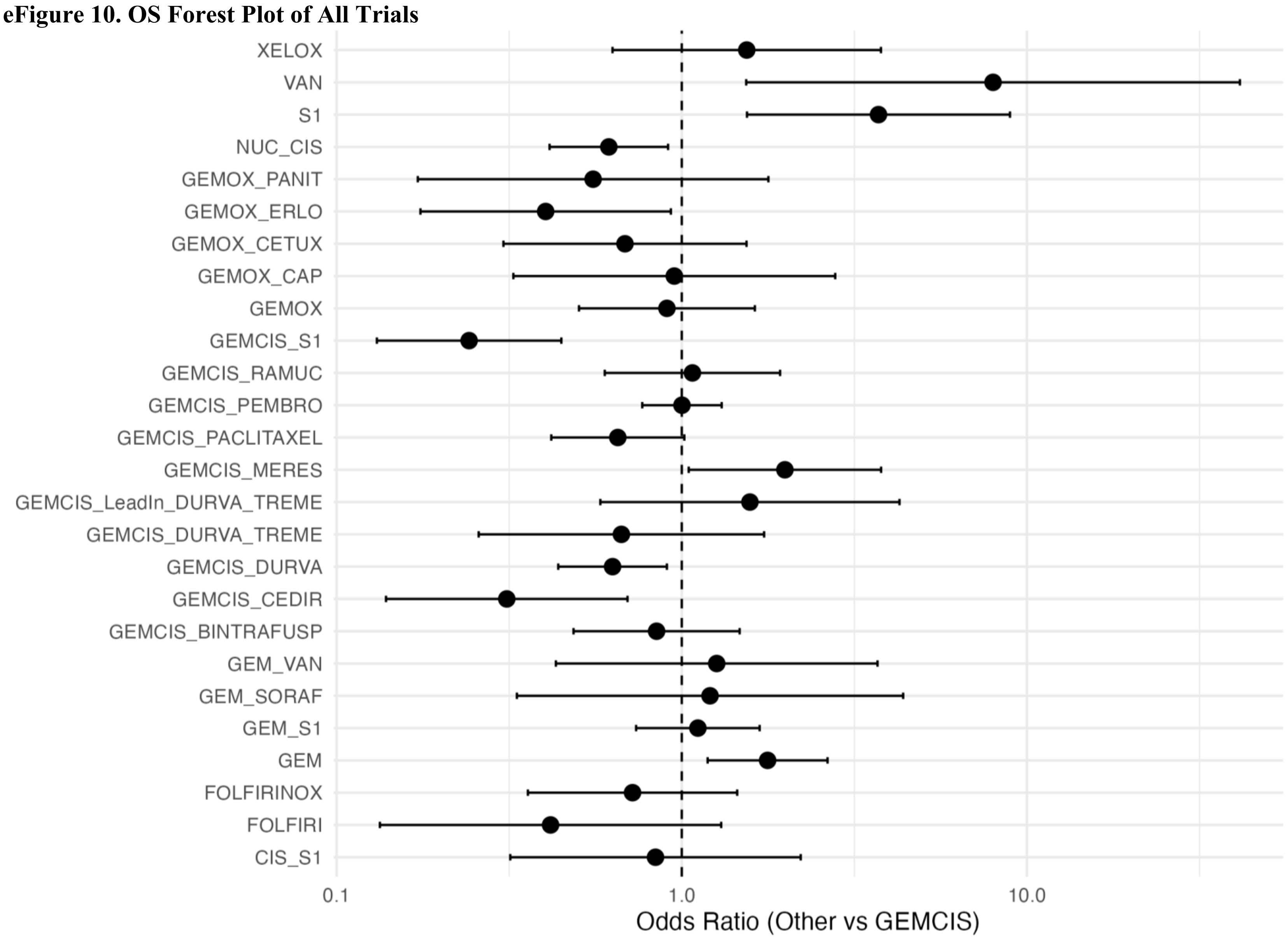

eTable 5. ECOG 0-1 Trials: PFS

Regimen	HR with CI (95%)	SUCRA Rank
Gemcitabine plus Cisplatin plus Durvalumab	0.800 (0.663–0.965)	1
Gemcitabine plus Cisplatin plus Durvalumab plus Tremelimumab	0.755 (0.405–1.408)	2
Gemcitabine plus Cisplatin (Lead-in) plus Durvalumab plus Tremelimumab	0.784 (0.422–1.456)	3
Gemcitabine plus S-1	0.862 (0.695–1.069)	4
Gemcitabine plus Cisplatin plus Merestinib	0.909 (0.724–1.141)	5
Gemcitabine plus Cisplatin plus Cediranib	0.909 (0.639–1.294)	6
Gemcitabine plus Cisplatin plus Bintrafusp Alfa	1.031 (0.748–1.421)	7
Fluorouracil plus Leucovorin plus Irinotecan plus Oxaliplatin	1.176 (0.727–1.903)	8
Gemcitabine plus Cisplatin plus Ramucirumab	1.124 (0.903–1.399)	9
Gemcitabine	1.515 (0.947–2.425)	10
Gemcitabine plus Sorafenib	1.943 (1.013–3.724)	11
S-1	1.973 (1.225–3.176)	12

*Notes: HR = Hazard Ratio; CI = Confidence Interval; SUCRA = Surface Under the Cumulative Ranking curve. SUCRA ranks are assigned based on descending order of adjusted P-scores, with higher P-scores indicating better efficacy and assigned ranks from 1 (best) to 27 (worst) among the 27 regimens. Only regimens with complete HR and CI data are included in the ranking.

© 2026 Elemosho A et al. *JAMA Network Open*.

eFigure 11. PFS Network Graph of ECOG 0-1 Trials

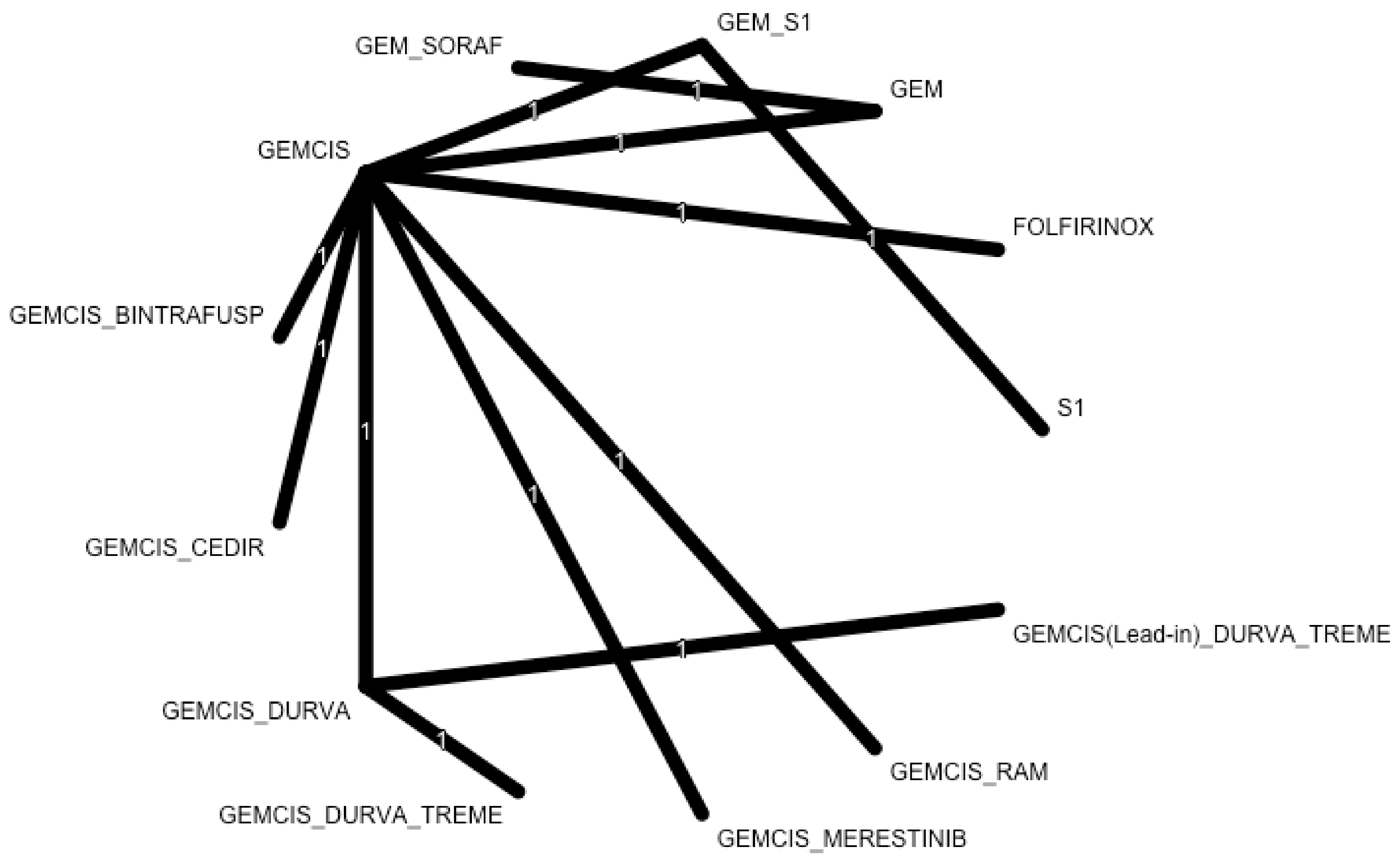

eFigure 12. ECOG 0-1 PFS SUCRA

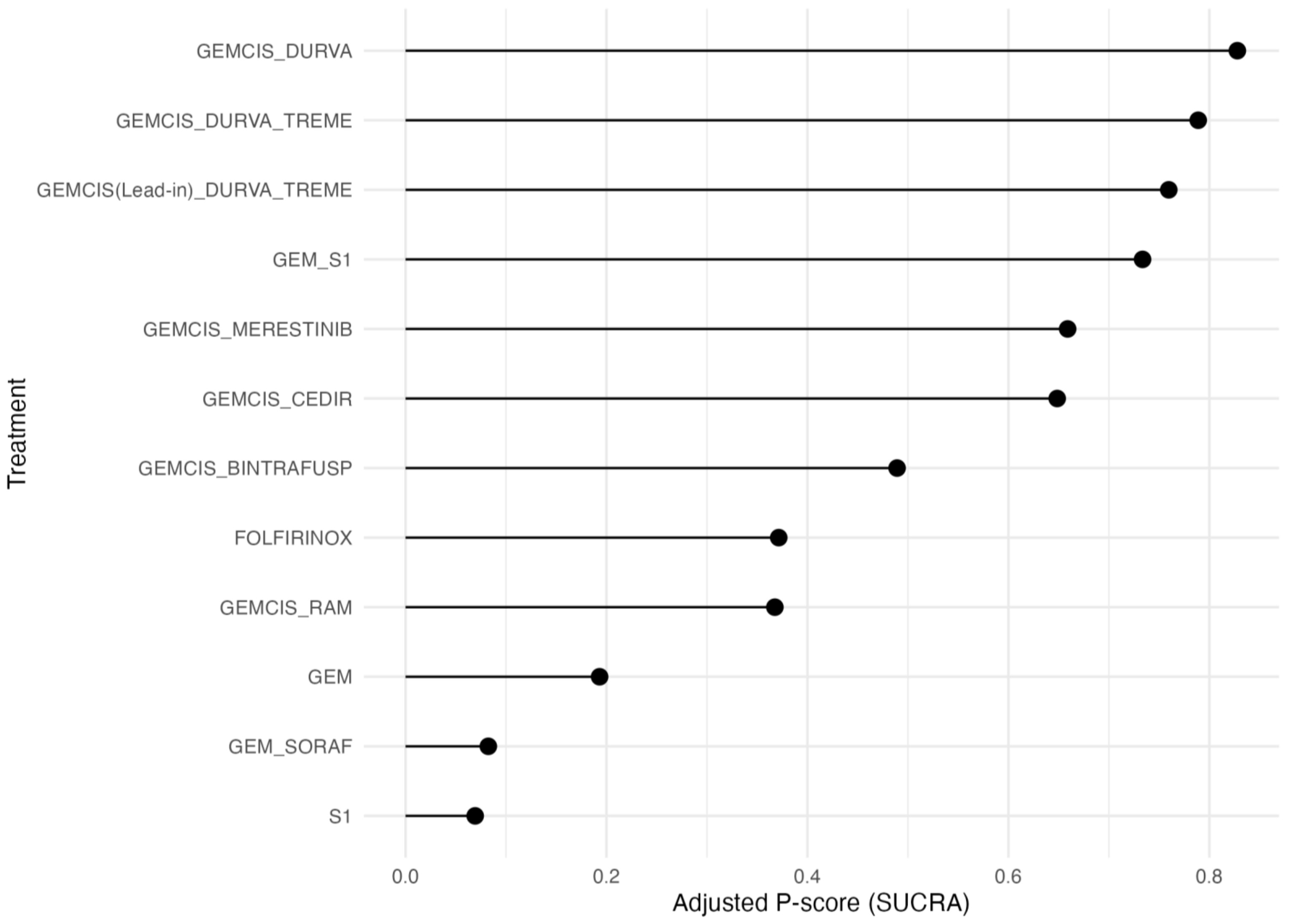

© 2026 Elemosho A et al. *JAMA Network Open*.

eFigure 13. PFS Forest Plot ECOG 0-1 Trials

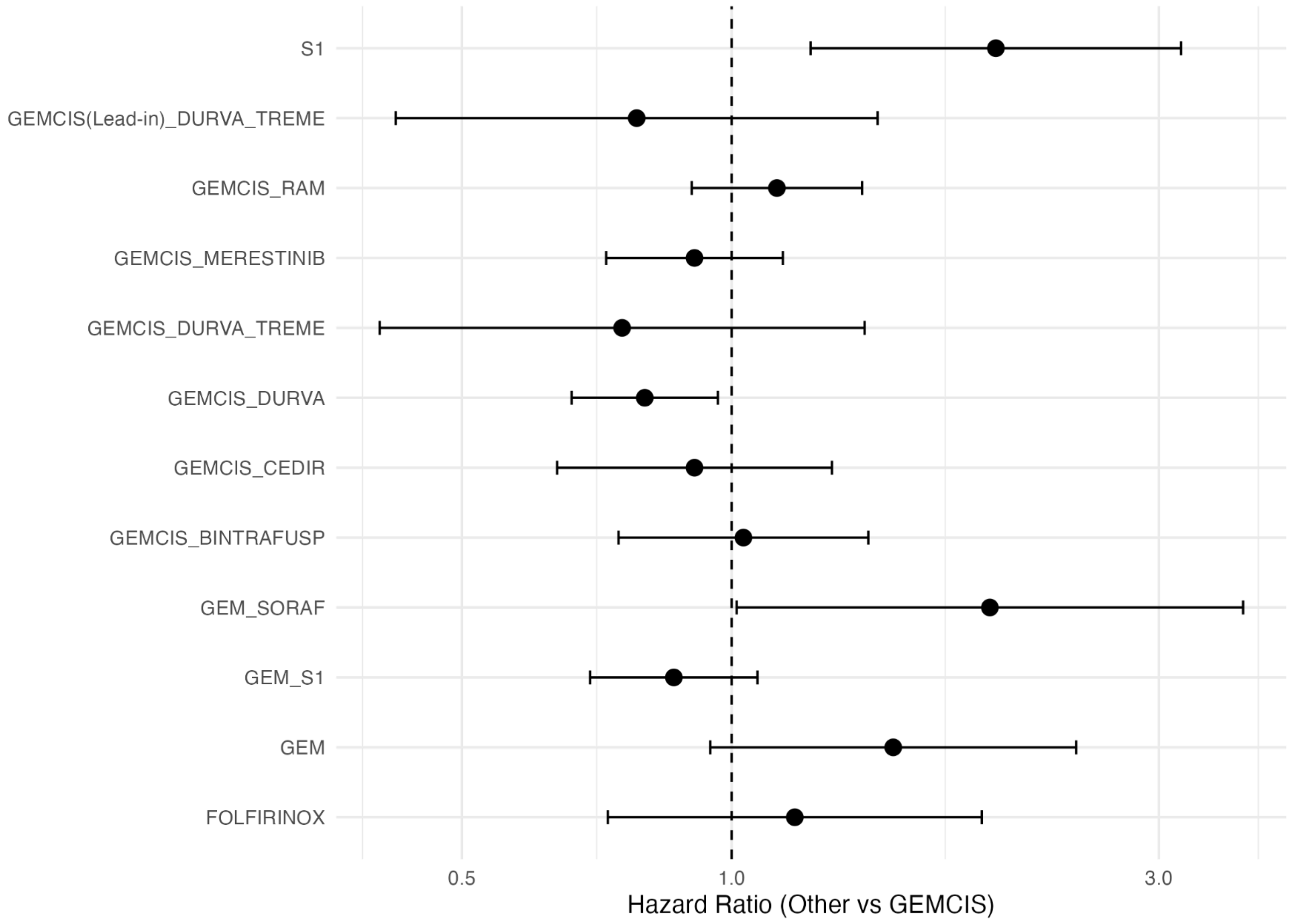

eTable 6. Overall Survival (ECOG 0-1)

Regimen	HR with CI (95%)	SUCRA Rank
Gemcitabine plus Cisplatin plus Durvalumab	0.714 (0.607–0.840)	1
Gemcitabine plus Cisplatin plus Durvalumab plus Tremelimumab	0.703 (0.426–1.161)	2
Gemcitabine plus Cisplatin (Lead-in) plus Durvalumab plus Tremelimumab	0.776 (0.409–1.475)	3
Gemcitabine plus Cisplatin plus Cediranib	0.862 (0.584–1.272)	4
Gemcitabine plus Cisplatin plus Merestinib	0.909 (0.645–1.281)	5
Gemcitabine plus S-1	0.943 (0.778–1.144)	6
S-1	1.098 (0.667–1.807)	7
Gemcitabine plus Cisplatin plus Bintrafusp Alfa	1.235 (0.634–2.405)	8
Gemcitabine plus Cisplatin plus Pembrolizumab	1.205 (1.049–1.384)	9
Fluorouracil plus Leucovorin plus Irinotecan plus Oxaliplatin	1.282 (0.907–1.813)	10
Gemcitabine plus Cisplatin plus Ramucirumab	1.333 (0.961–1.850)	11
Gemcitabine	1.449 (0.884–2.377)	12
Gemcitabine plus Sorafenib	1.746 (0.883–3.454)	13

*Notes: HR = Hazard Ratio; CI = Confidence Interval; SUCRA = Surface Under the Cumulative Ranking curve. SUCRA ranks are assigned based on descending order of adjusted P-scores, with higher P-scores indicating better efficacy and assigned ranks from 1 (best) to 27 (worst) among the 27 regimens. Only regimens with complete HR and CI data are included in the ranking.

© 2026 Elemosho A et al. *JAMA Network Open*.

eFigure 14. OS Network Graph of ECOG 0-1 Trials

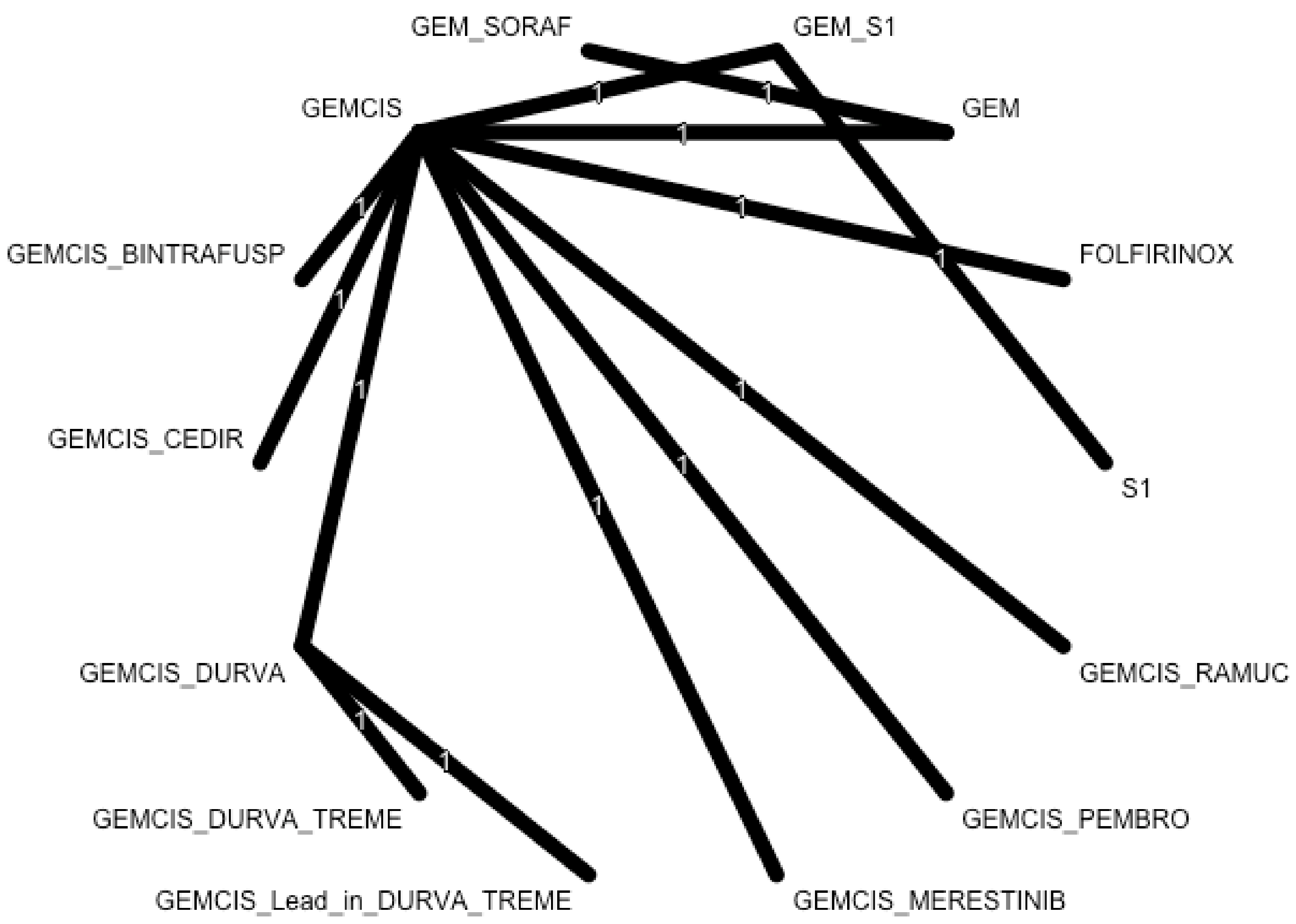

eFigure 15. ECOG 0-1 OS SUCRA

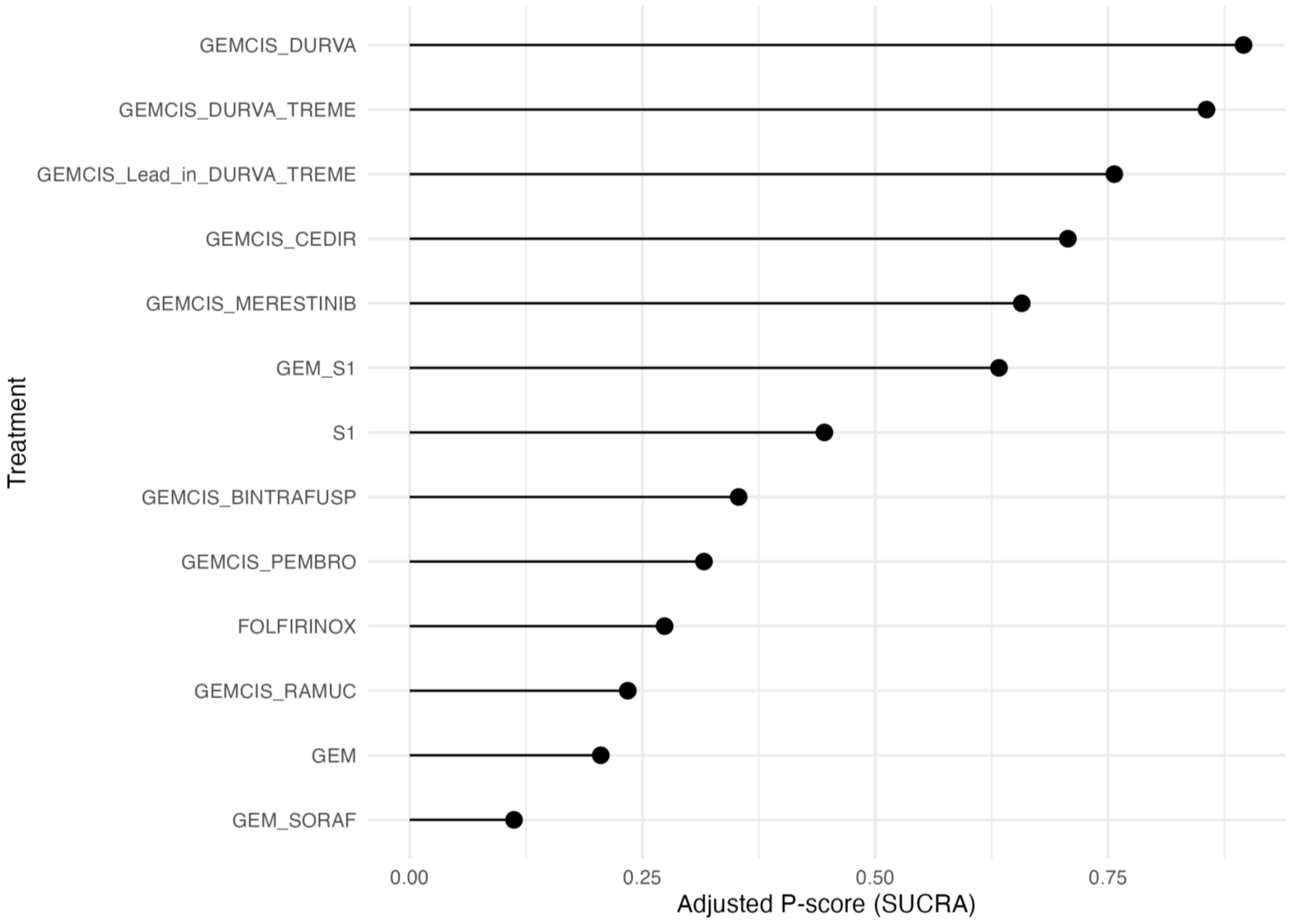

© 2026 Elemosho A et al. *JAMA Network Open*.

eFigure 16. ECOG 0-1 OS Forest Plot

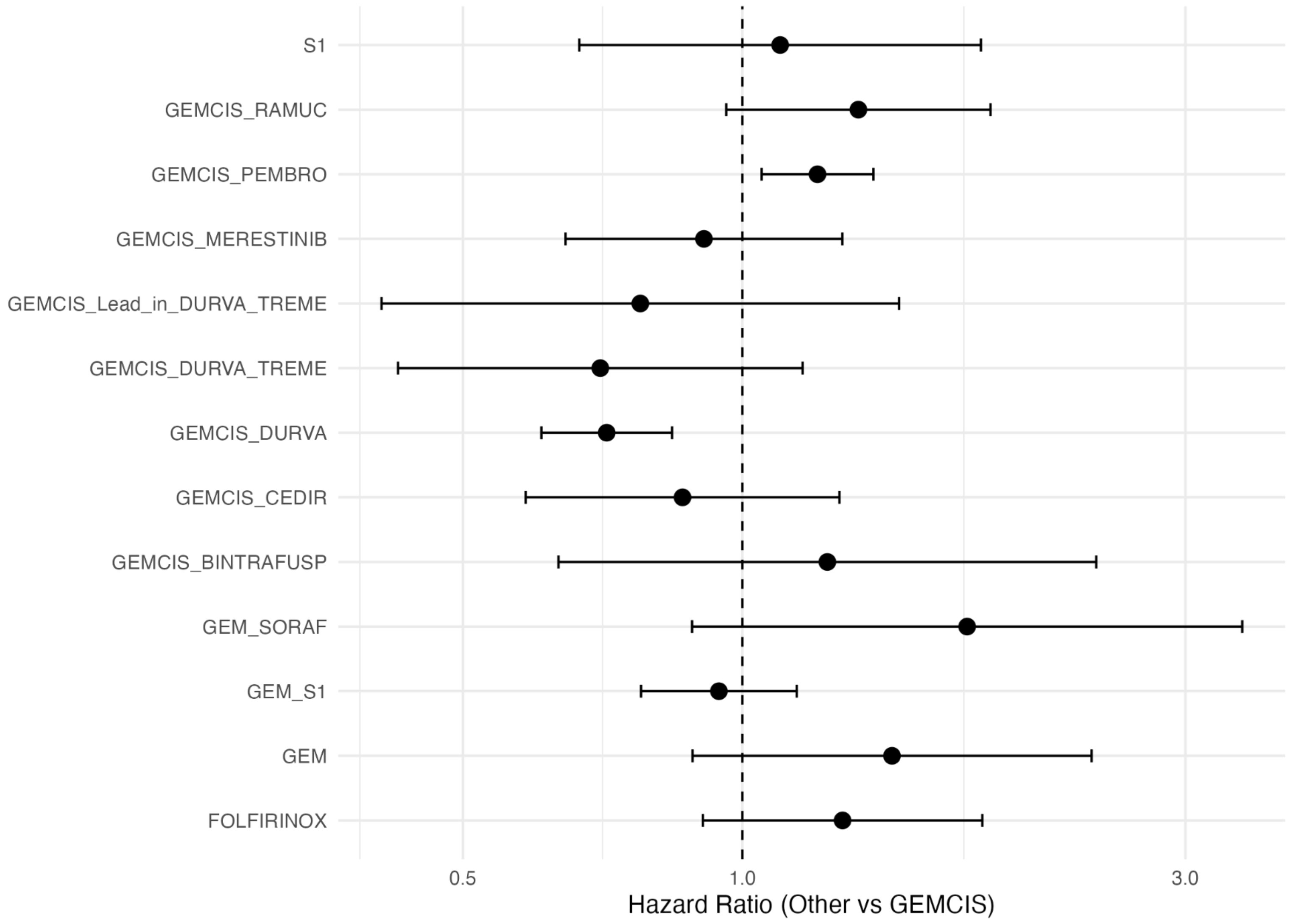

eTable 7. Phase 3 Trials PFS

Regimen	HR with CI (95%)	SUCRA Rank
Gemcitabine plus Oxaliplatin plus Erlotinib	0.727 (0.511–1.035)	1
Gemcitabine plus Cisplatin plus S-1	0.752 (0.585–0.966)	2
Gemcitabine plus Cisplatin plus Durvalumab	0.800 (0.663–0.965)	3
Capecitabine plus Oxaliplatin	0.819 (0.571–1.174)	4
Gemcitabine plus Cisplatin plus Pembrolizumab	0.847 (0.746–0.963)	5
Gemcitabine plus S-1	0.862 (0.695–1.069)	6
Gemcitabine plus Oxaliplatin	0.909 (0.718–1.151)	7
Gemcitabine plus Cisplatin plus Paclitaxel	0.926 (0.730–1.174)	8
Gemcitabine plus Cisplatin plus Pemetrexed	1.031 (0.748–1.421)	9
Nuc plus Cisplatin	1.449 (1.176–1.786)	10
Gemcitabine	1.587 (1.292–1.950)	11

*Notes: HR = Hazard Ratio; CI = Confidence Interval; SUCRA = Surface Under the Cumulative Ranking curve. SUCRA ranks are assigned based on descending order of adjusted P-scores, with higher P-scores indicating better efficacy and assigned ranks from 1 (best) to 27 (worst) among the 27 regimens. Only regimens with complete HR and CI data are included in the ranking.

© 2026 Elemosho A et al. *JAMA Network Open*.

eFigure 17. PFS Network Graph of Phase 3 Trials

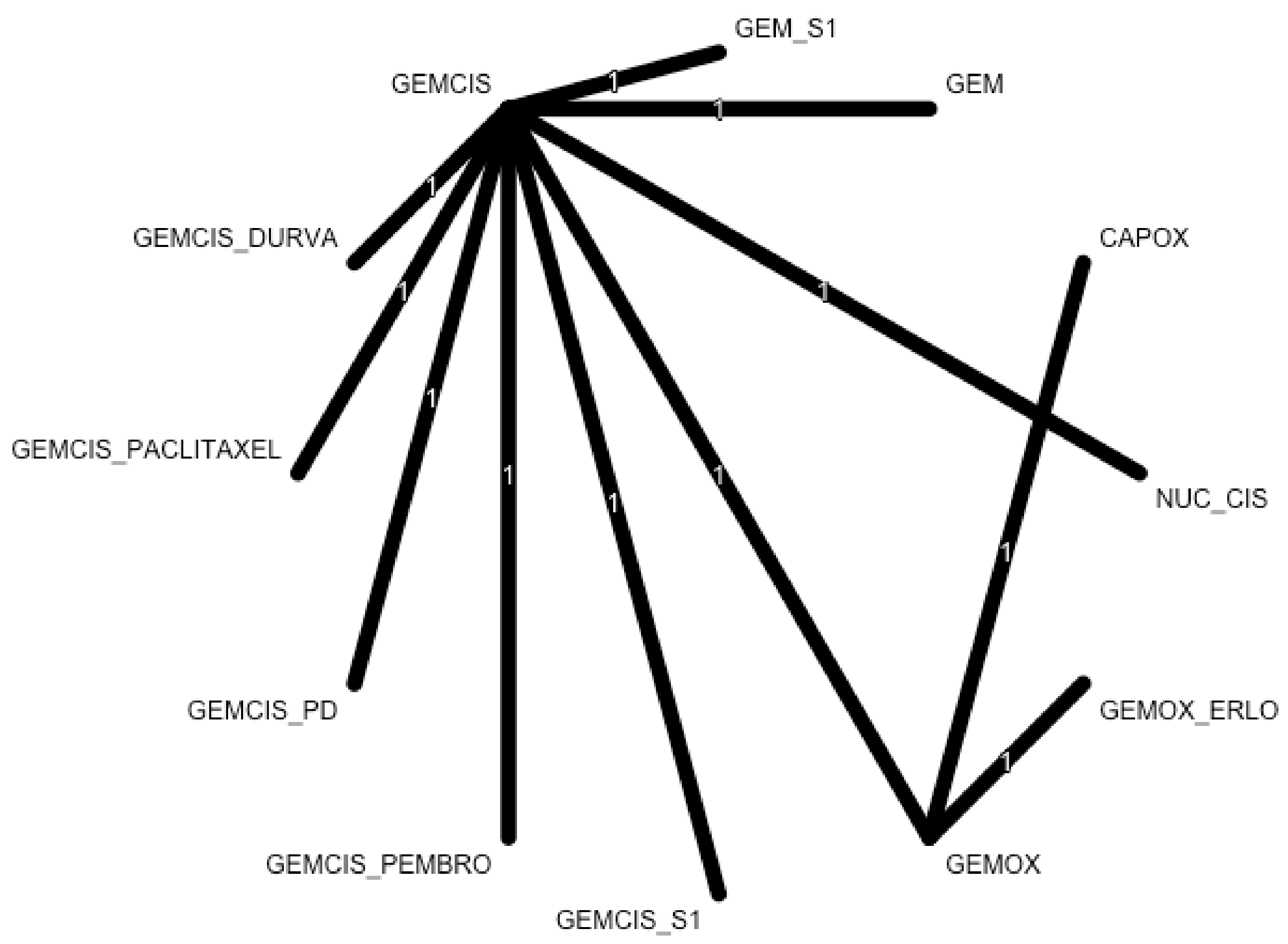

eFigure 18. PFS SUCRA of Phase 3 Trials

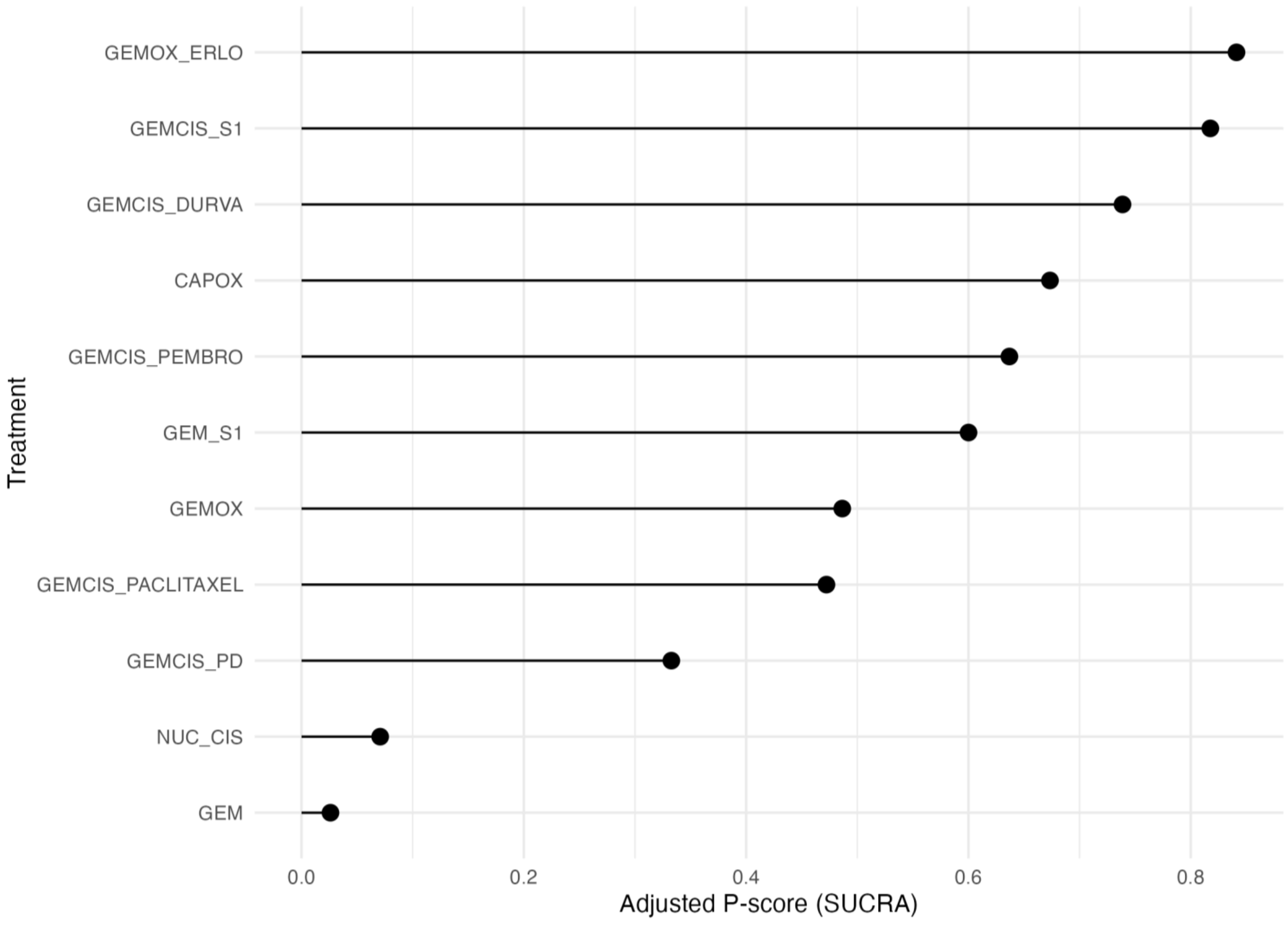

© 2026 Elemosho A et al. *JAMA Network Open*.

eFigure 19. PFS Forest Plot of HRs of Phase 3 Trials

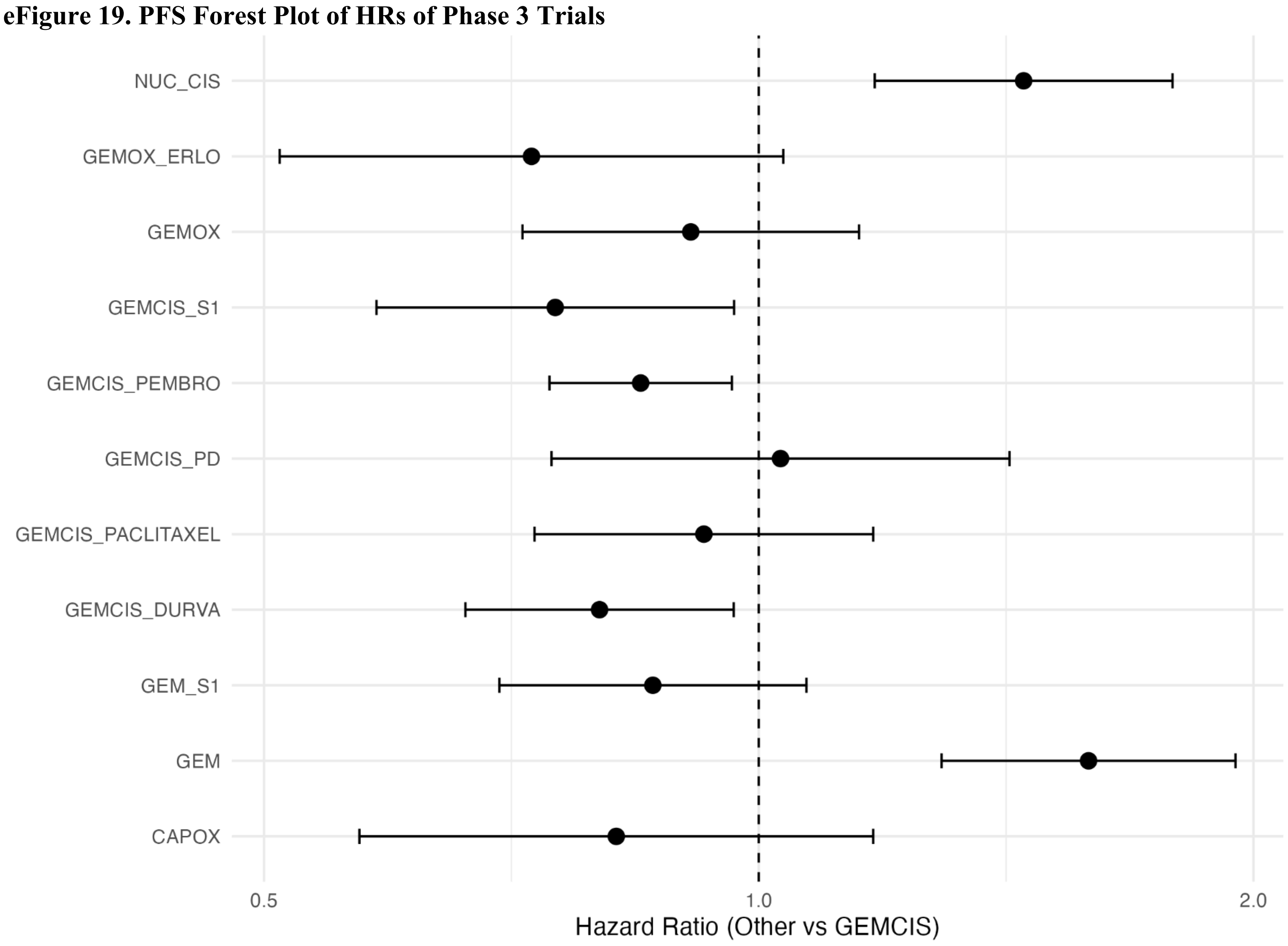

eTable 8. OS Phase 3 Trials

Regimen	HR with CI (95%)	SUCRA Rank
Capecitabine plus Oxaliplatin	0.635 (0.441–0.916)	1
Gemcitabine plus Cisplatin plus Durvalumab	0.714 (0.607–0.840)	2
Gemcitabine plus Oxaliplatin plus Erlotinib	0.723 (0.503–1.040)	3
Gemcitabine plus Oxaliplatin	0.781 (0.635–0.962)	4
Gemcitabine plus Cisplatin plus S-1	0.813 (0.646–1.023)	5
Gemcitabine plus Cisplatin plus Paclitaxel	0.909 (0.717–1.152)	6
Gemcitabine plus S-1	0.943 (0.778–1.144)	7
Gemcitabine plus Cisplatin plus Bintrafusp Alfa	1.235 (0.634–2.405)	8
Gemcitabine plus Cisplatin plus Pembrolizumab	1.205 (1.049–1.384)	9
Gemcitabine	1.563 (1.260–1.938)	10
Nuc plus Cisplatin	1.786 (1.271–2.510)	11

*Notes: HR = Hazard Ratio; CI = Confidence Interval; SUCRA = Surface Under the Cumulative Ranking curve. SUCRA ranks are assigned based on descending order of adjusted P-scores, with higher P-scores indicating better efficacy and assigned ranks from 1 (best) to 27 (worst) among the 27 regimens. Only regimens with complete HR and CI data are included in the ranking.

© 2026 Elemosho A et al. *JAMA Network Open*.

eFigure 20. OS Network Graph of Phase 3 Trials

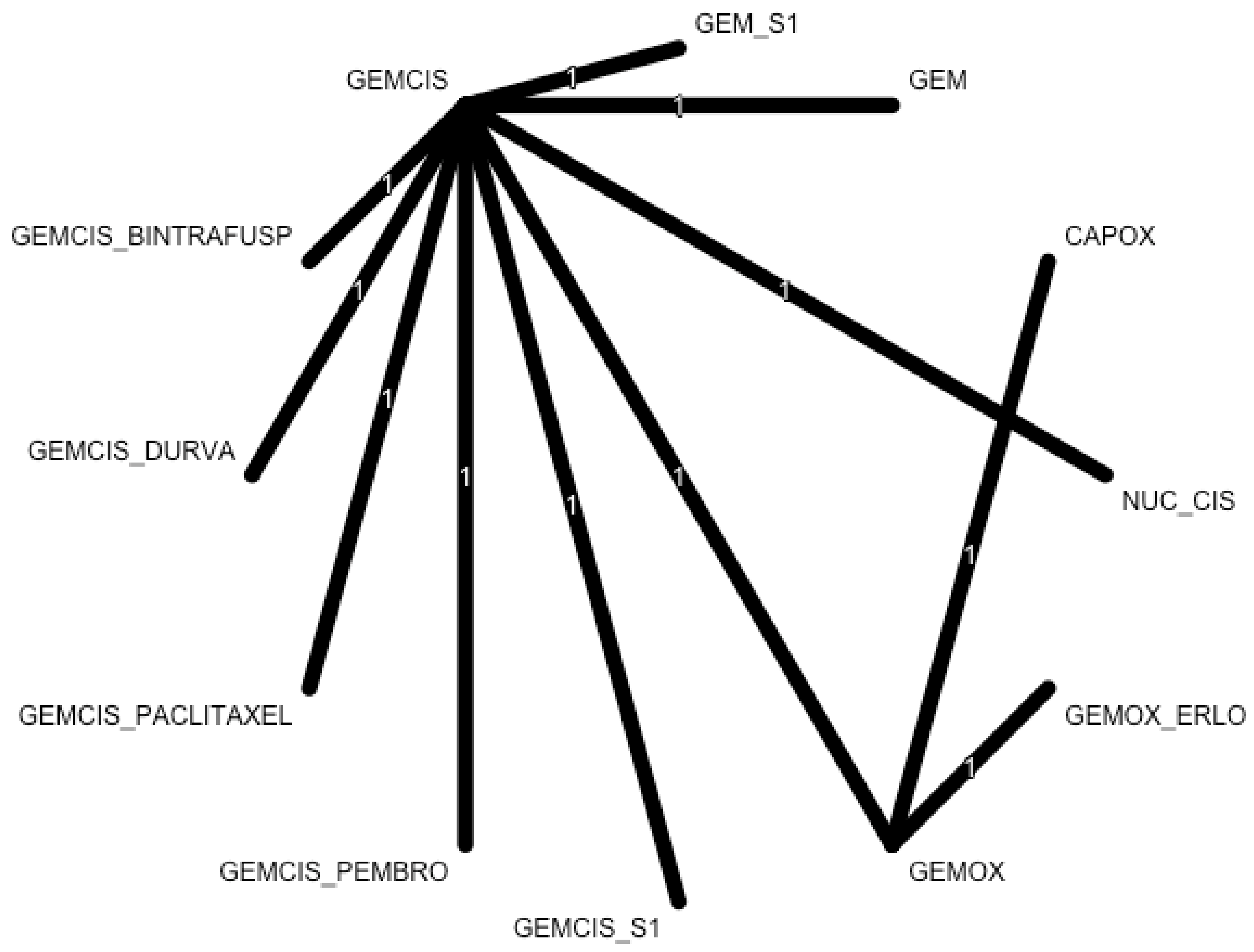

eFigure 21. OS SUCRA of Phase 3 Trials

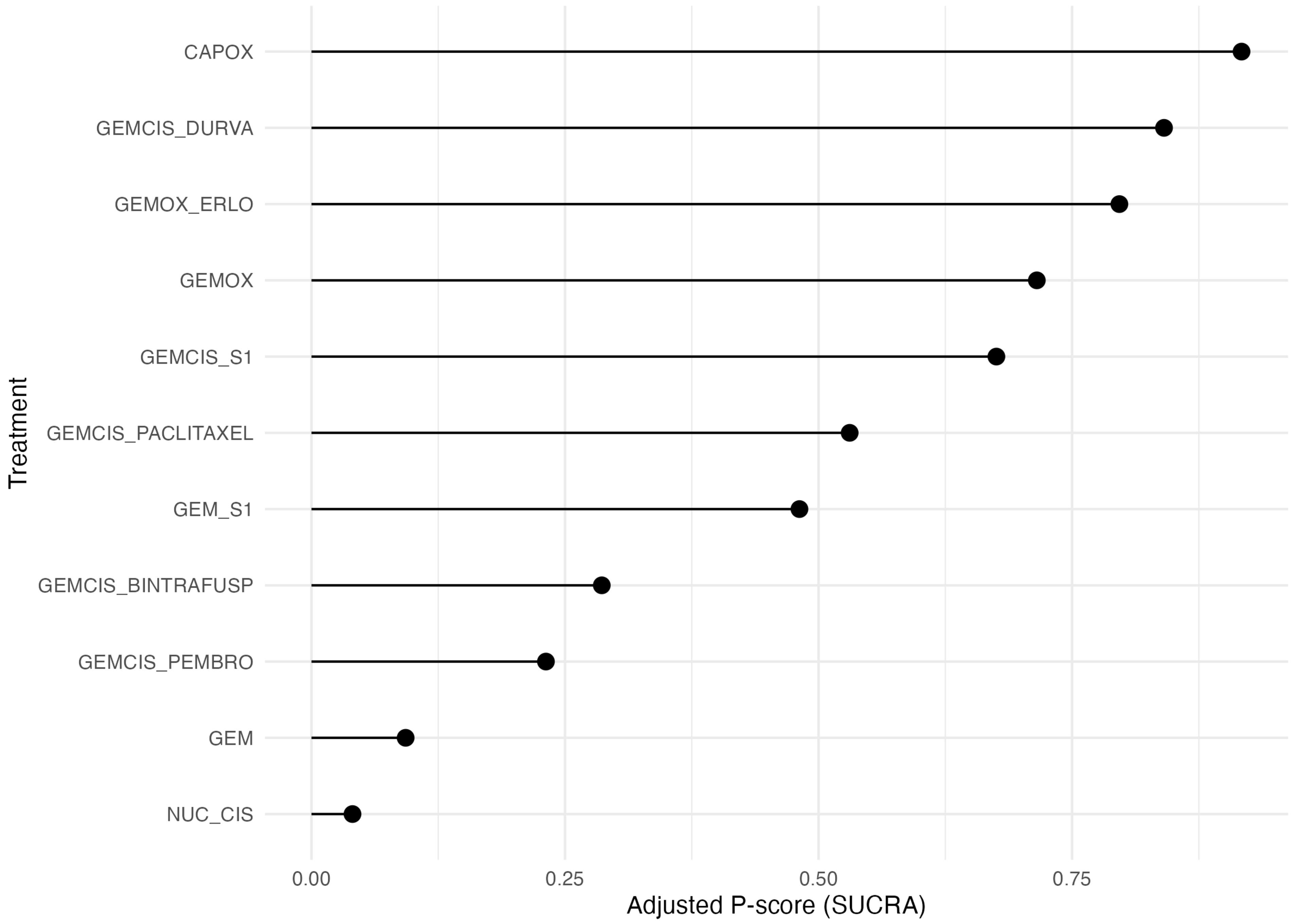

© 2026 Elemosho A et al. *JAMA Network Open*.

eFigure 22. OS Forest Plot of Phase 3 Trials

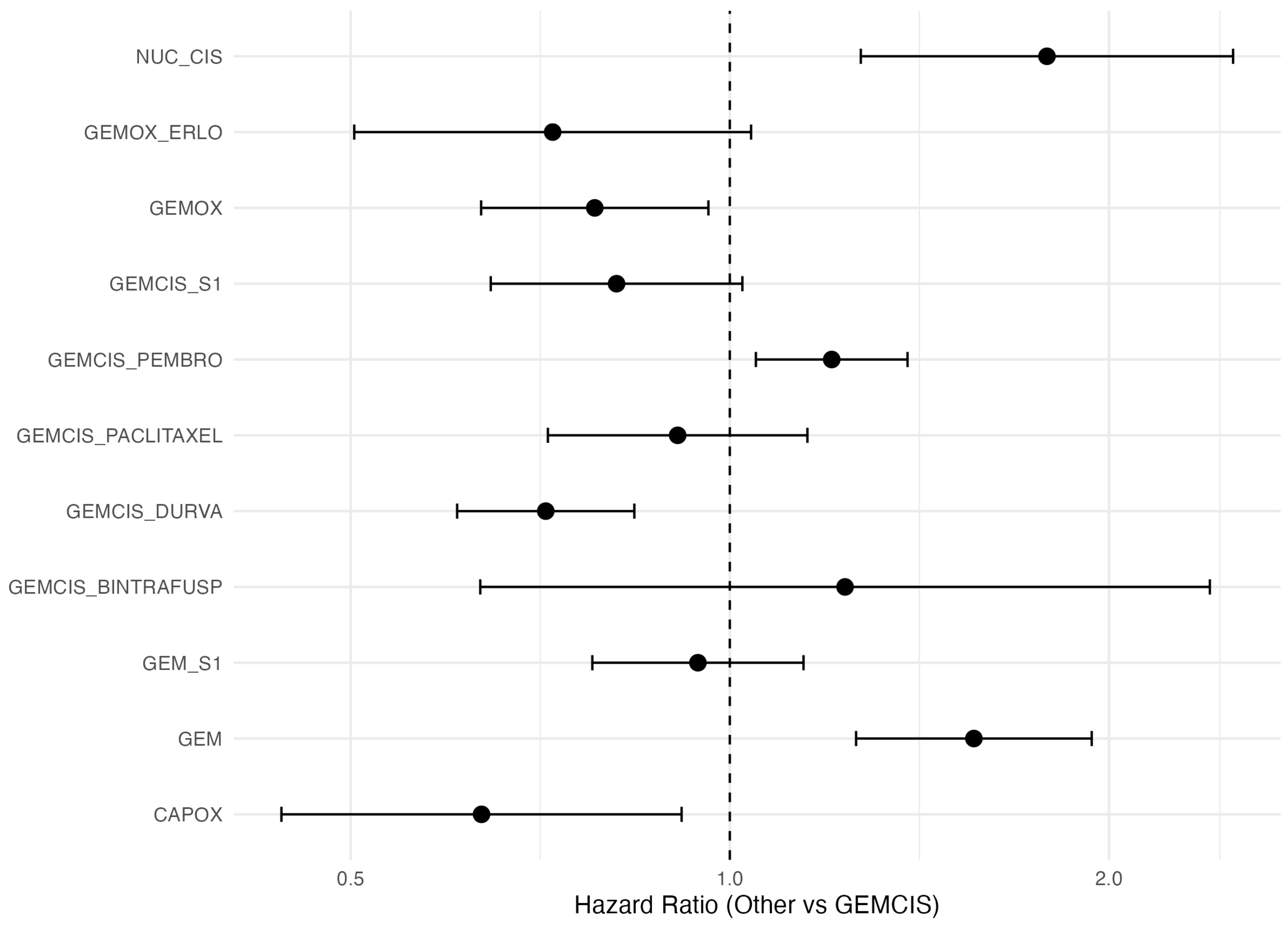

eTable 9 Asian Trials PFS

Regimen	HR with CI (95%)	SUCRA Rank
Gemcitabine plus Cisplatin plus Sintilimab plus Anlotinib	0.476 (0.284–0.799)	1
Gemcitabine plus Oxaliplatin plus Cetuximab	0.610 (0.389–0.958)	2
Gemcitabine plus Oxaliplatin plus Erlotinib	0.727 (0.511–1.035)	3
Gemcitabine plus Cisplatin plus S-1	0.752 (0.585–0.966)	4
Capecitabine plus Oxaliplatin	0.819 (0.571–1.174)	5
Gemcitabine plus S-1	0.890 (0.728–1.088)	6
Gemcitabine plus Oxaliplatin	0.909 (0.718–1.151)	7
Gemcitabine plus Cisplatin plus Bintrafusp Alfa	1.031 (0.748–1.421)	8
Cisplatin plus S-1	1.176 (0.727–1.903)	9
Gemcitabine	1.302 (0.968–1.751)	10
S-1	1.777 (1.207–2.616)	11

*Notes: HR = Hazard Ratio; CI = Confidence Interval; SUCRA = Surface Under the Cumulative Ranking curve. SUCRA ranks are assigned based on descending order of adjusted P-scores, with higher P-scores indicating better efficacy and assigned ranks from 1 (best) to 27 (worst) among the 27 regimens. Only regimens with complete HR and CI data are included in the ranking.

© 2026 Elemosho A et al. *JAMA Network Open*.

eFigure 23. PFS Network Graph of Asian Trials

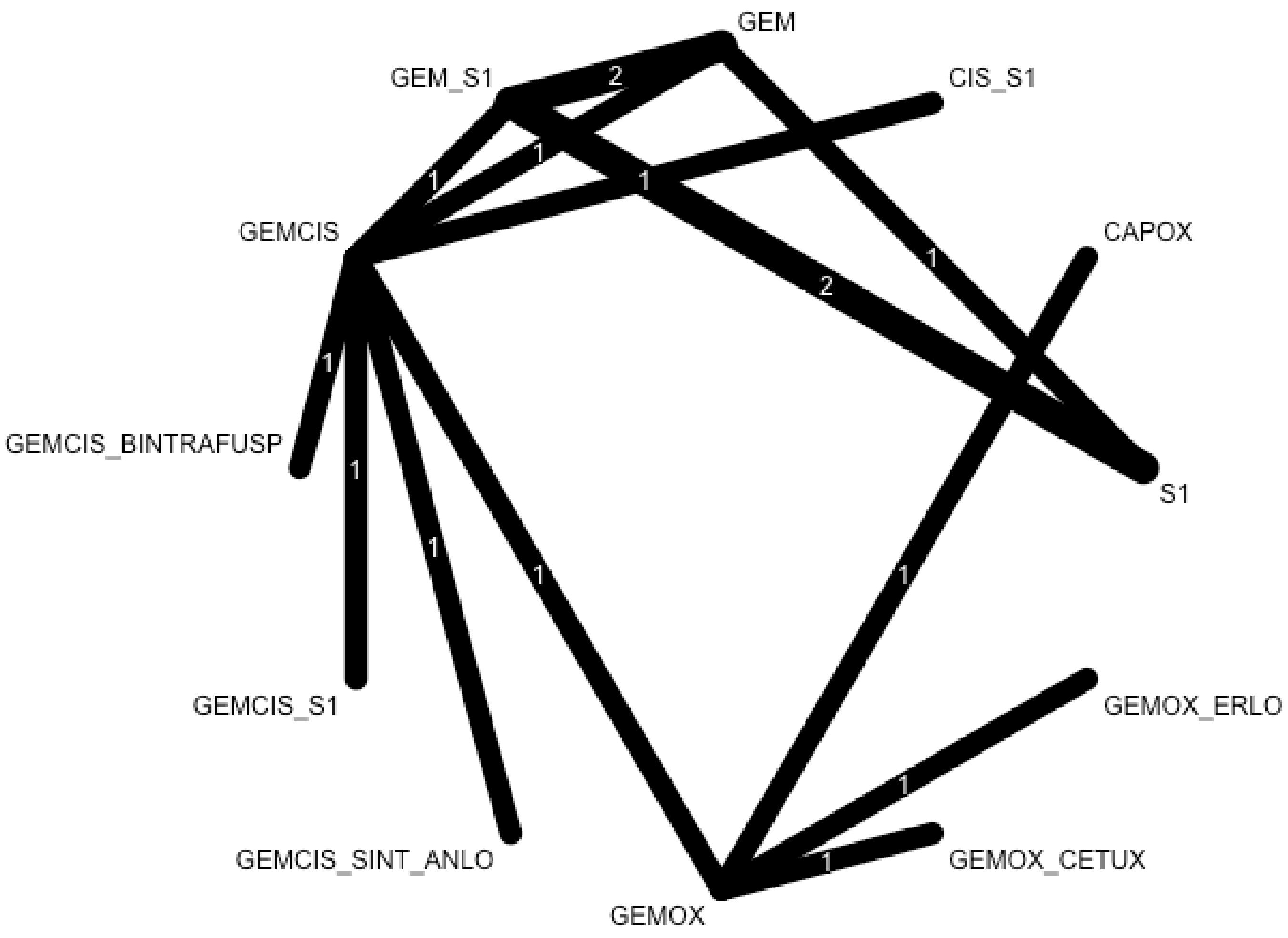

eFigure 24. PFS SUCRA of Asian Trials

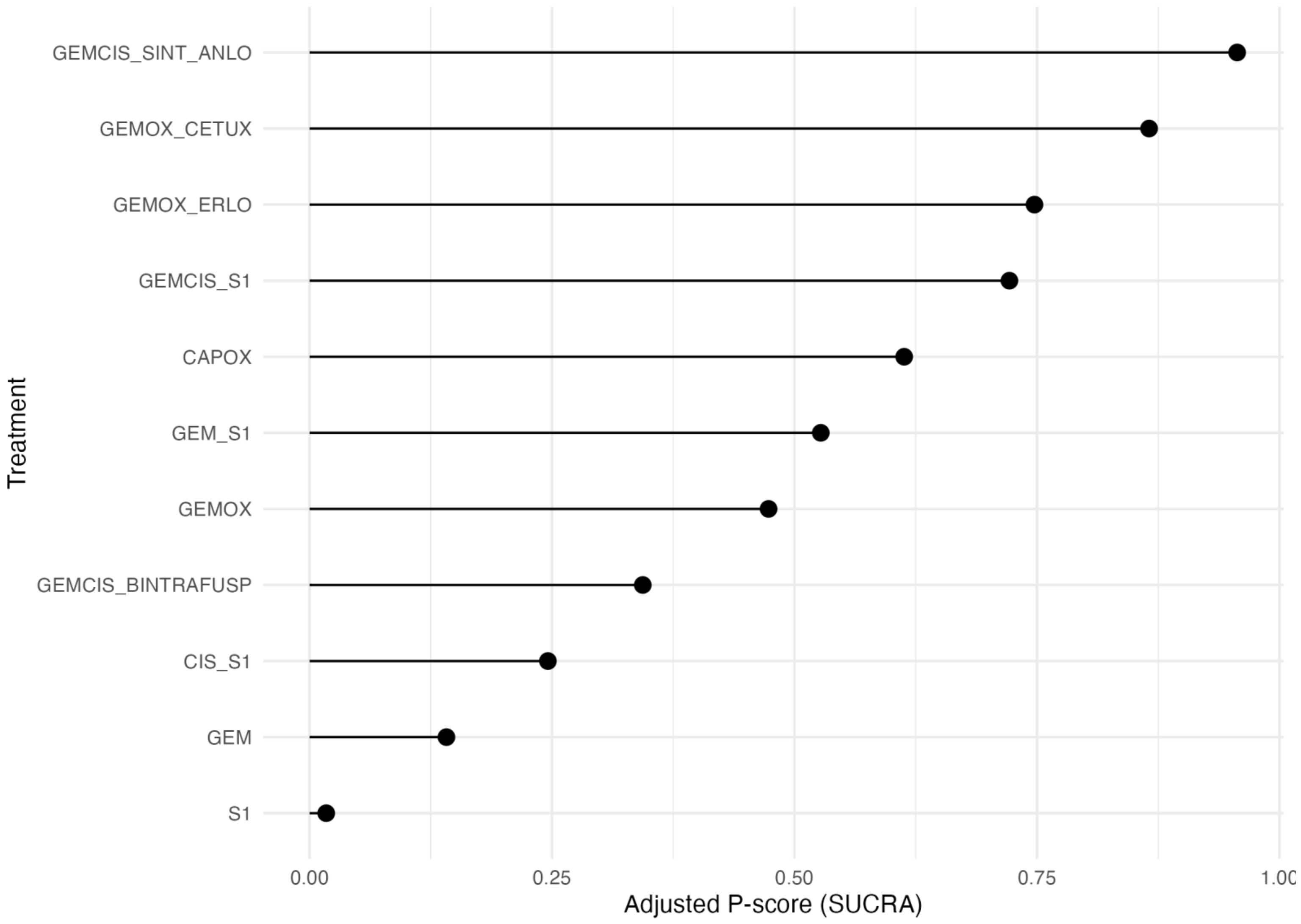

© 2026 Elemosho A et al. *JAMA Network Open*.

eFigure 25 PFS Forest Plot of Asian Trials

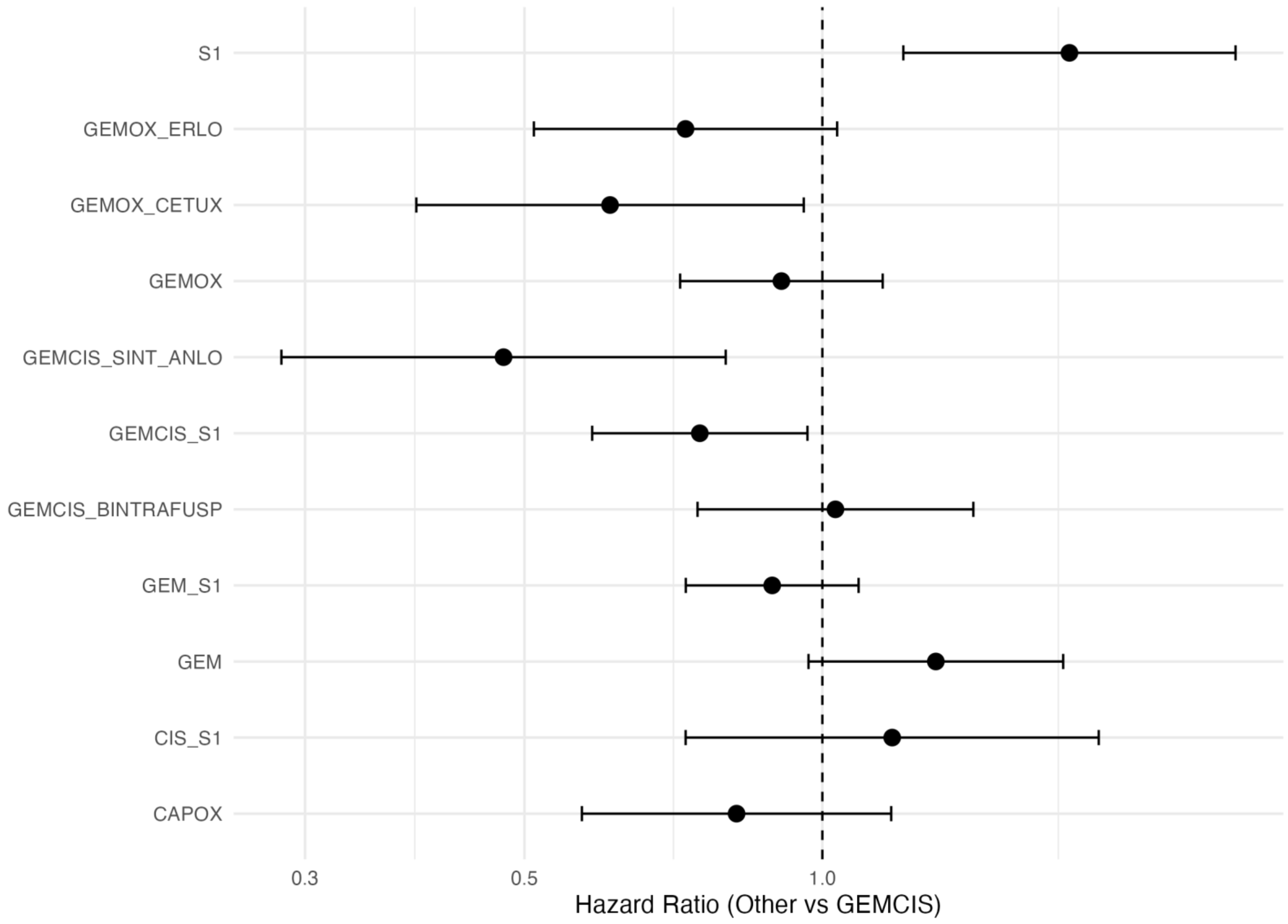

eTable 10. OS Asian Trials

Regimen	HR with CI (95%)	SUCRA Rank
Capecitabine plus Oxaliplatin	0.635 (0.441–0.916)	1
Gemcitabine plus Oxaliplatin plus Cetuximab	0.635 (0.434–0.929)	2
Gemcitabine plus Oxaliplatin plus Erlotinib	0.723 (0.503–1.040)	3
Gemcitabine plus Oxaliplatin	0.781 (0.635–0.962)	4
Gemcitabine plus Cisplatin plus S-1	0.813 (0.646–1.023)	5
Gemcitabine plus S-1	0.951 (0.793–1.141)	6
S-1	0.985 (0.770–1.260)	7
Gemcitabine plus Cisplatin plus Sintilimab plus Anlotinib	1.042 (0.539–2.012)	8
Gemcitabine plus Cisplatin plus Bintrafusp Alfa	1.235 (0.634–2.405)	9
Cisplatin plus S-1	1.389 (0.861–2.239)	10
Gemcitabine	1.376 (1.066–1.775)	11

*Notes: HR = Hazard Ratio; CI = Confidence Interval; SUCRA = Surface Under the Cumulative Ranking curve. SUCRA ranks are assigned based on descending order of adjusted P-scores, with higher P-scores indicating better efficacy and assigned ranks from 1 (best) to 27 (worst) among the 27 regimens. Only regimens with complete HR and CI data are included in the ranking.

© 2026 Elemosho A et al. *JAMA Network Open*.

eFigure 26 OS Network Graph of Asian Trials

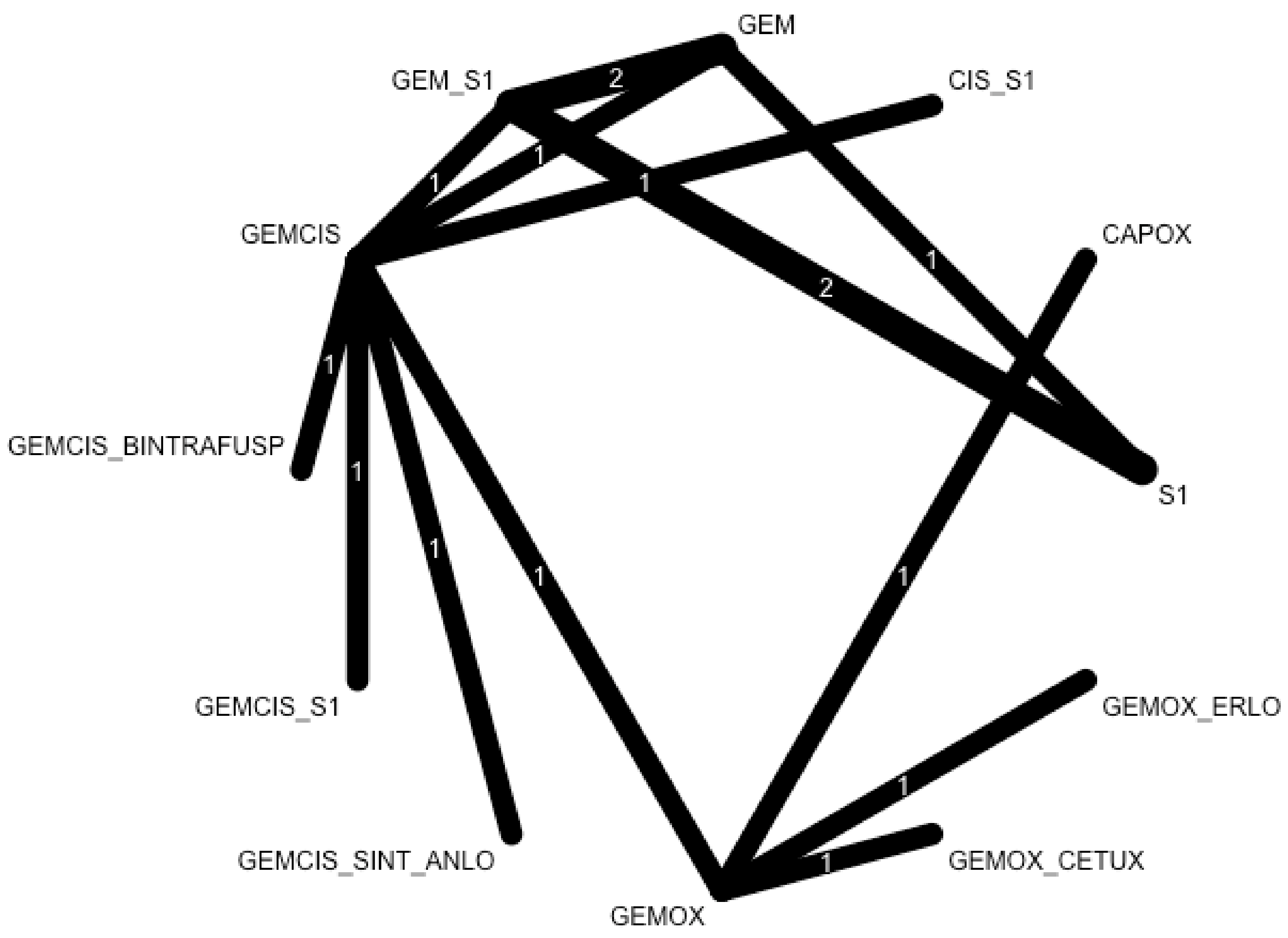

eFigure 27. OS SUCRA of Asian Trials

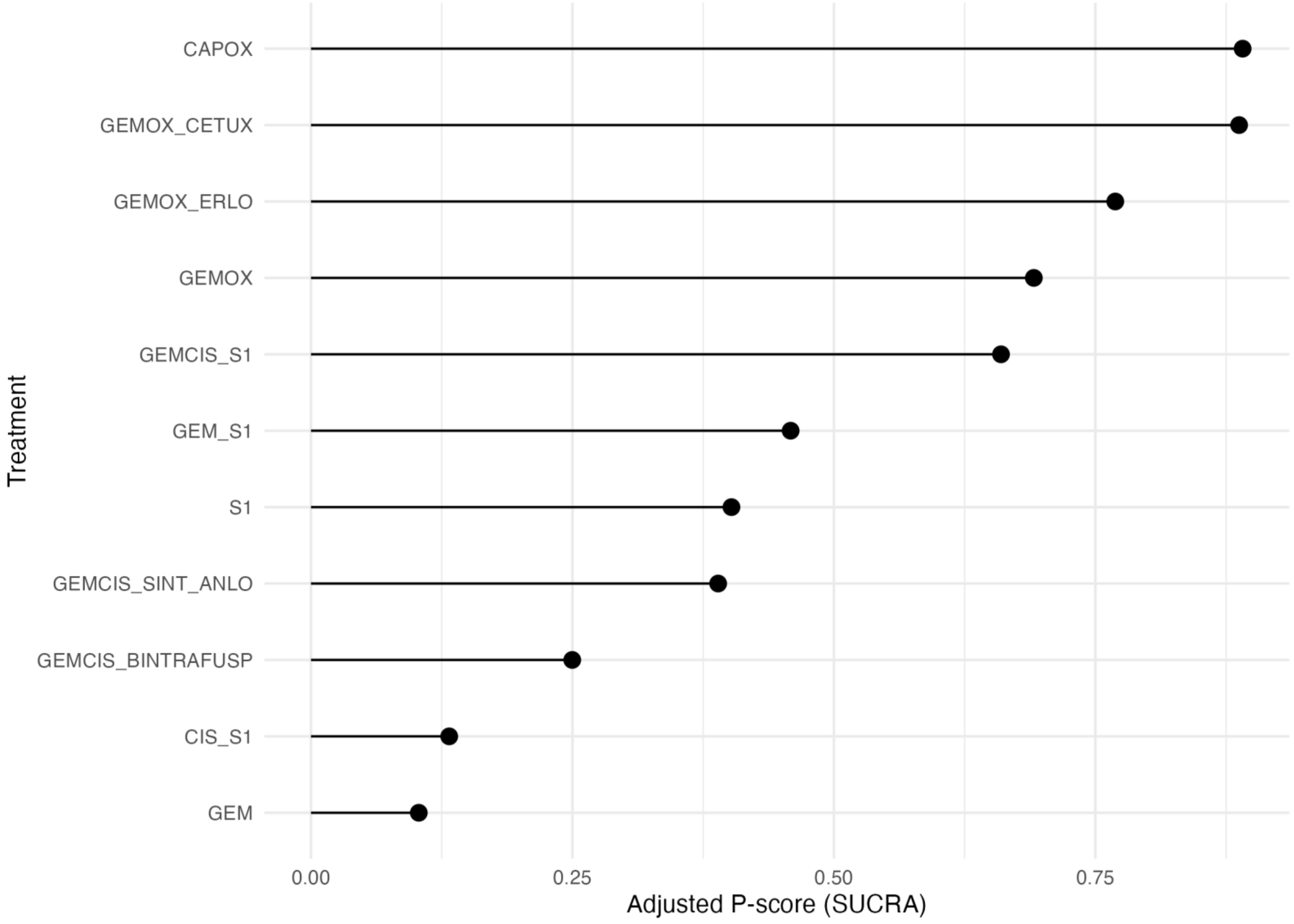

© 2026 Elemosho A et al. *JAMA Network Open*.

eFigure 28. OS Forest Plot of Asian Trials

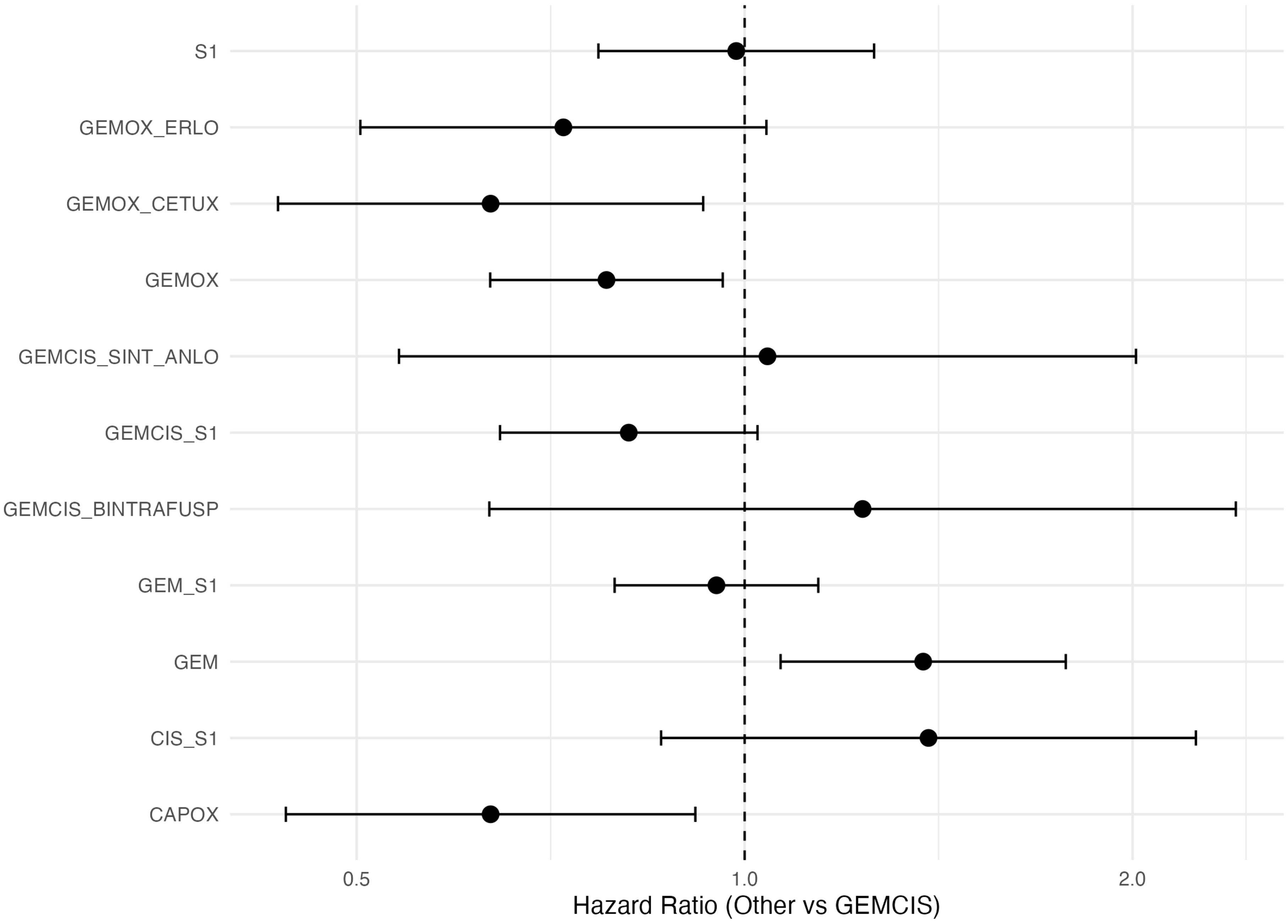

© 2026 Elemosho A et al. *JAMA Network Open*.

SAFETY

eTable 11. Anemia

Regimen	OR with CI	P-score Rank
Gemcitabine plus Cisplatin plus S-1	1.937 (0.83–4.50)	1
Gemcitabine plus Cisplatin plus Durvalumab	1.245 (0.82–1.88)	2
Gemcitabine plus Oxaliplatin	1.255 (0.70–2.26)	3
Gemcitabine plus Oxaliplatin plus Panitumumab	1.225 (0.21–7.20)	4
Gemcitabine plus Oxaliplatin plus Cetuximab	1.061 (0.27–4.23)	5

*P Score is Ranked Worst (1) to Best (5)

eFigure 29. Network Graph of Anemia

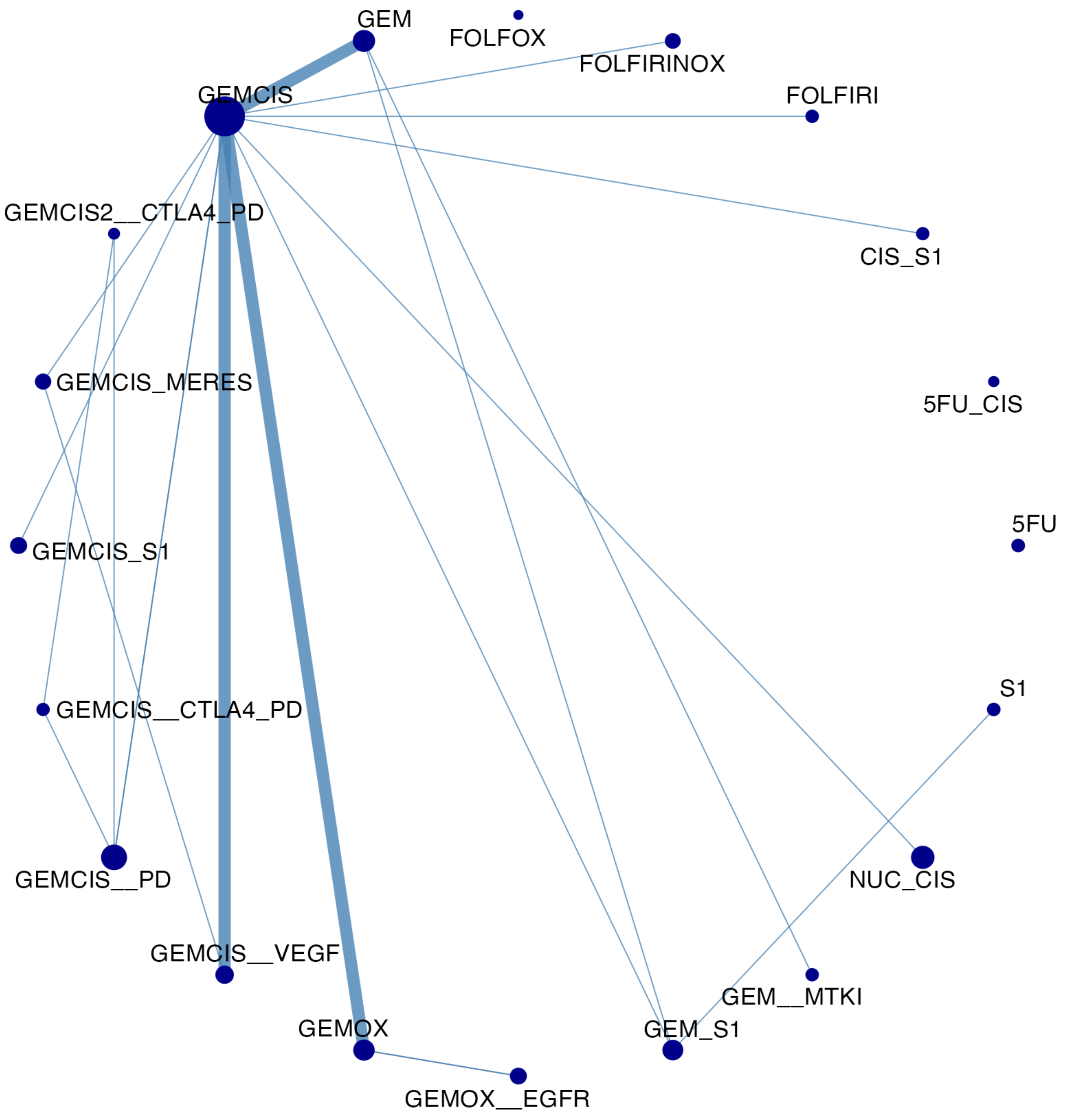

© 2026 Elemosho A et al. *JAMA Network Open*.

eTable 12. Thrombocytopenia

Regimen	OR with CI	P-score Rank
Gemcitabine plus Cisplatin plus S-1	2.729 (0.05–157.38)	1
Gemcitabine plus Oxaliplatin	1.916 (0.09–38.79)	2
Gemcitabine plus Oxaliplatin plus Cetuximab	1.607 (0.02–109.99)	3
Gemcitabine plus Cisplatin plus Durvalumab	0.951 (0.02–53.12)	4
Gemcitabine plus Oxaliplatin plus Panitumumab	0.914 (0.00–236.39)	5

*P Score is Ranked Worst (1) to Best (5)

eFigure 30. Network Graph of Thrombocytopenia

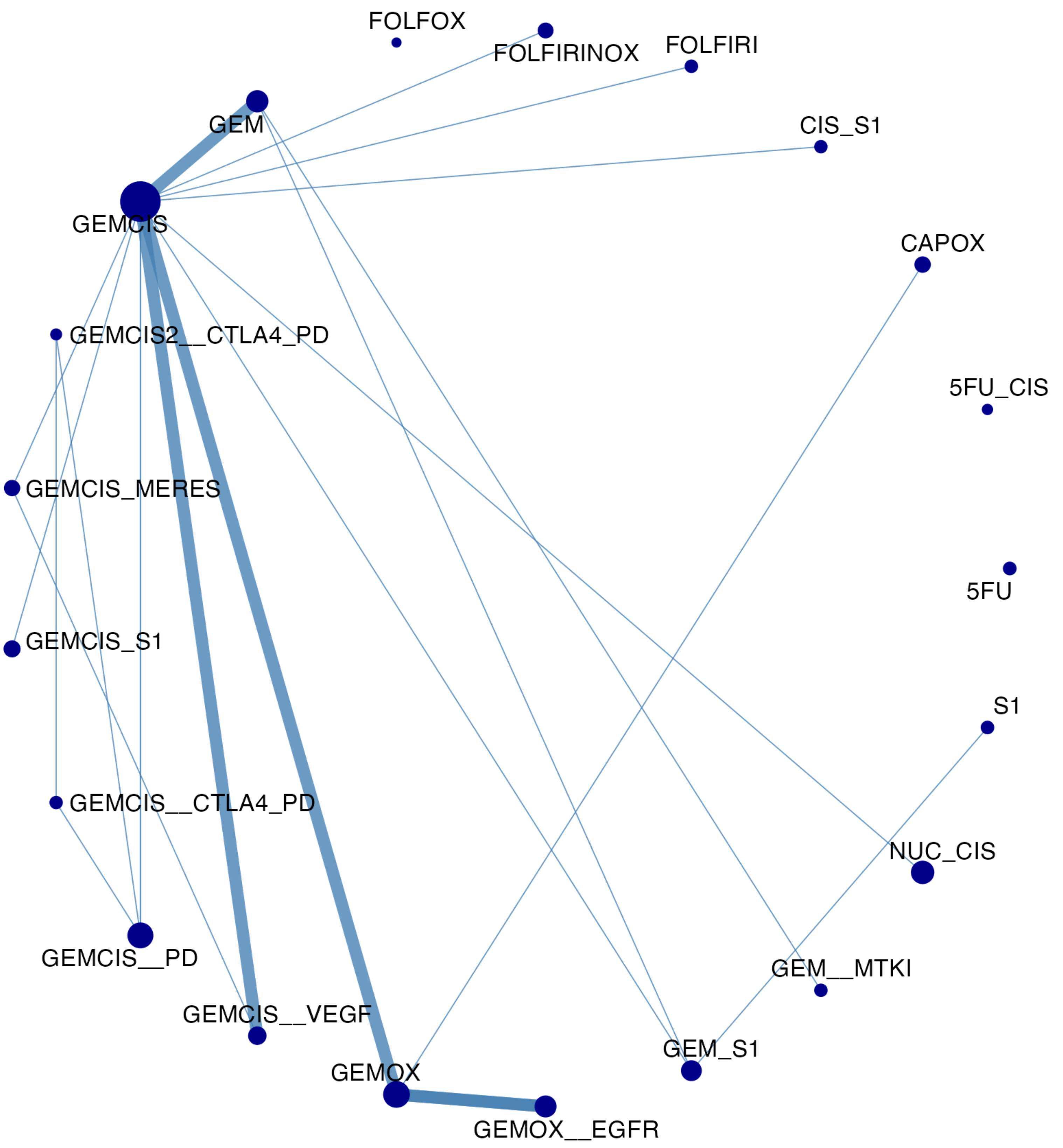

© 2026 Elemosho A et al. *JAMA Network Open*.

eTable 13. Neutropenia

Regimen	OR with CI	P-score Rank
Gemcitabine plus Oxaliplatin	5.946 (0.57–62.19)	1
Gemcitabine plus Oxaliplatin plus Panitumumab	8.918 (0.15–543.88)	2
Gemcitabine plus Oxaliplatin plus Cetuximab	2.080 (0.08–51.06)	3
Gemcitabine plus Cisplatin plus S-1	1.443 (0.08–25.55)	4
Gemcitabine plus Cisplatin plus Durvalumab	0.797 (0.05–13.80)	5

*P Score is Ranked Worst (1) to Best (5)

eFigure 31 Network Graph of Neutropenia

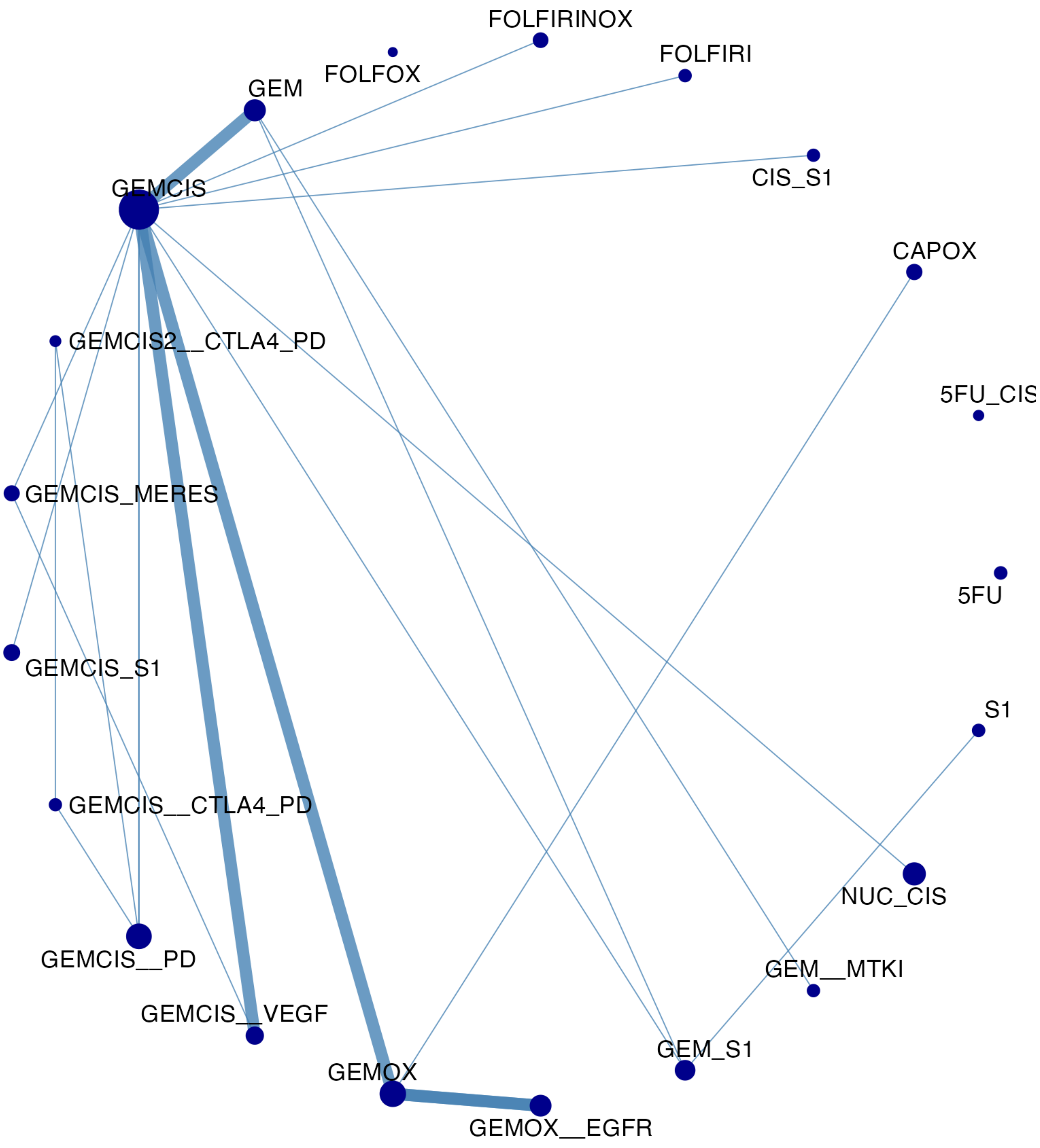

© 2026 Elemosho A et al. *JAMA Network Open*.

eTable 14. Nausea

Regimen	OR with CI	P-score Rank
Gemcitabine plus Oxaliplatin plus Cetuximab	106.324 (3.49–3236.55)	1
Gemcitabine plus Oxaliplatin plus Panitumumab	40.779 (4.00–415.37)	2
Gemcitabine plus Oxaliplatin	19.904 (4.34–91.32)	3
Gemcitabine plus Cisplatin plus S-1	1.000 (0.14–7.21)	4

*P Score is Ranked Worst (1) to Best (5)

eFigure 32. Network Graph of Nausea

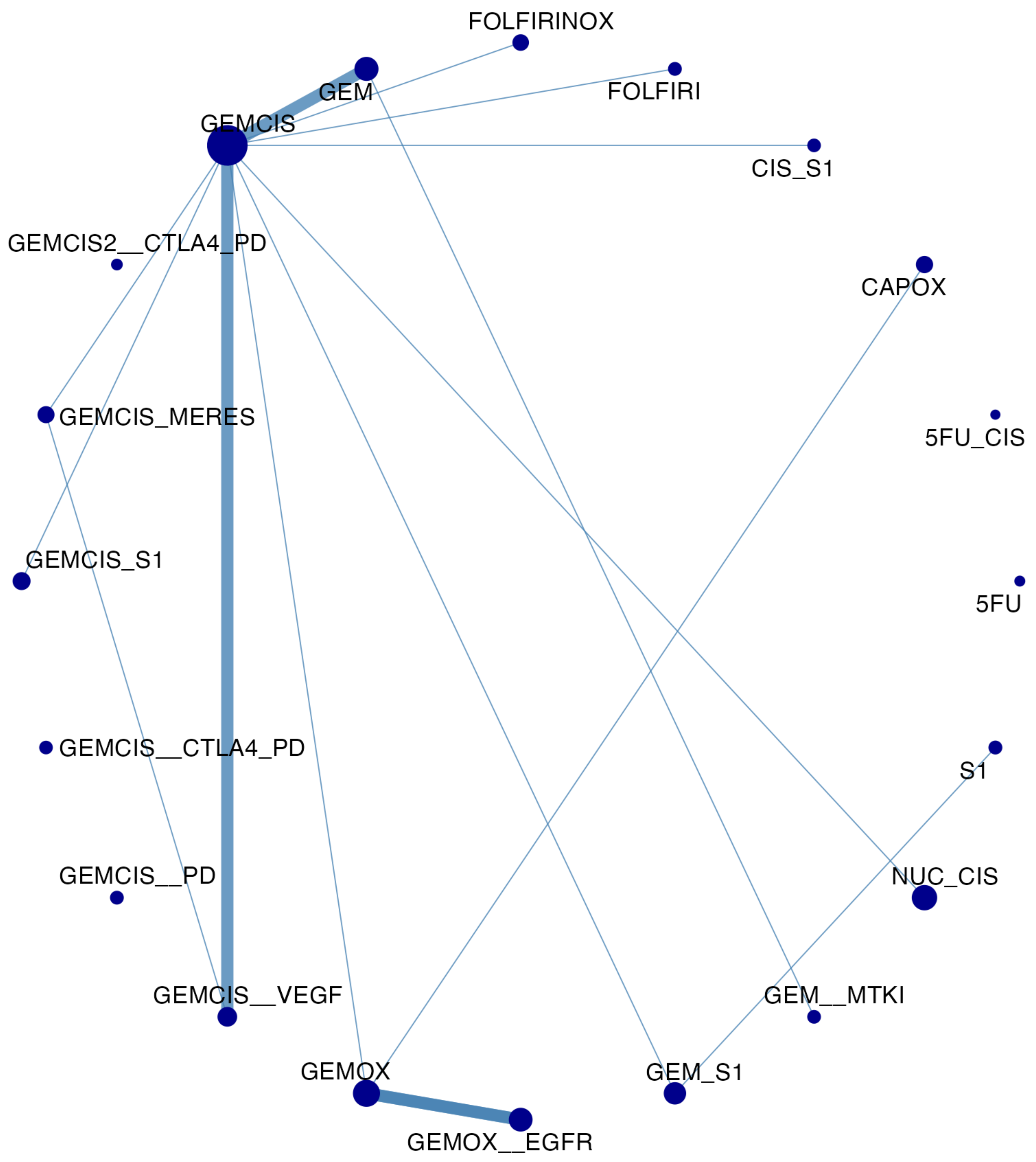

© 2026 Elemosho A et al. *JAMA Network Open*.

eTable 15. Vomiting

Regimen	OR with CI	P-score Rank
Gemcitabine plus Oxaliplatin	8.125 (1.72–38.35)	1
Gemcitabine plus Oxaliplatin plus Panitumumab	7.940 (0.32–195.65)	2
Gemcitabine plus Oxaliplatin plus Cetuximab	5.492 (0.50–59.98)	3

*P Score is Ranked Worst (1) to Best (5)

eFigure 33 Network Graph of Vomiting

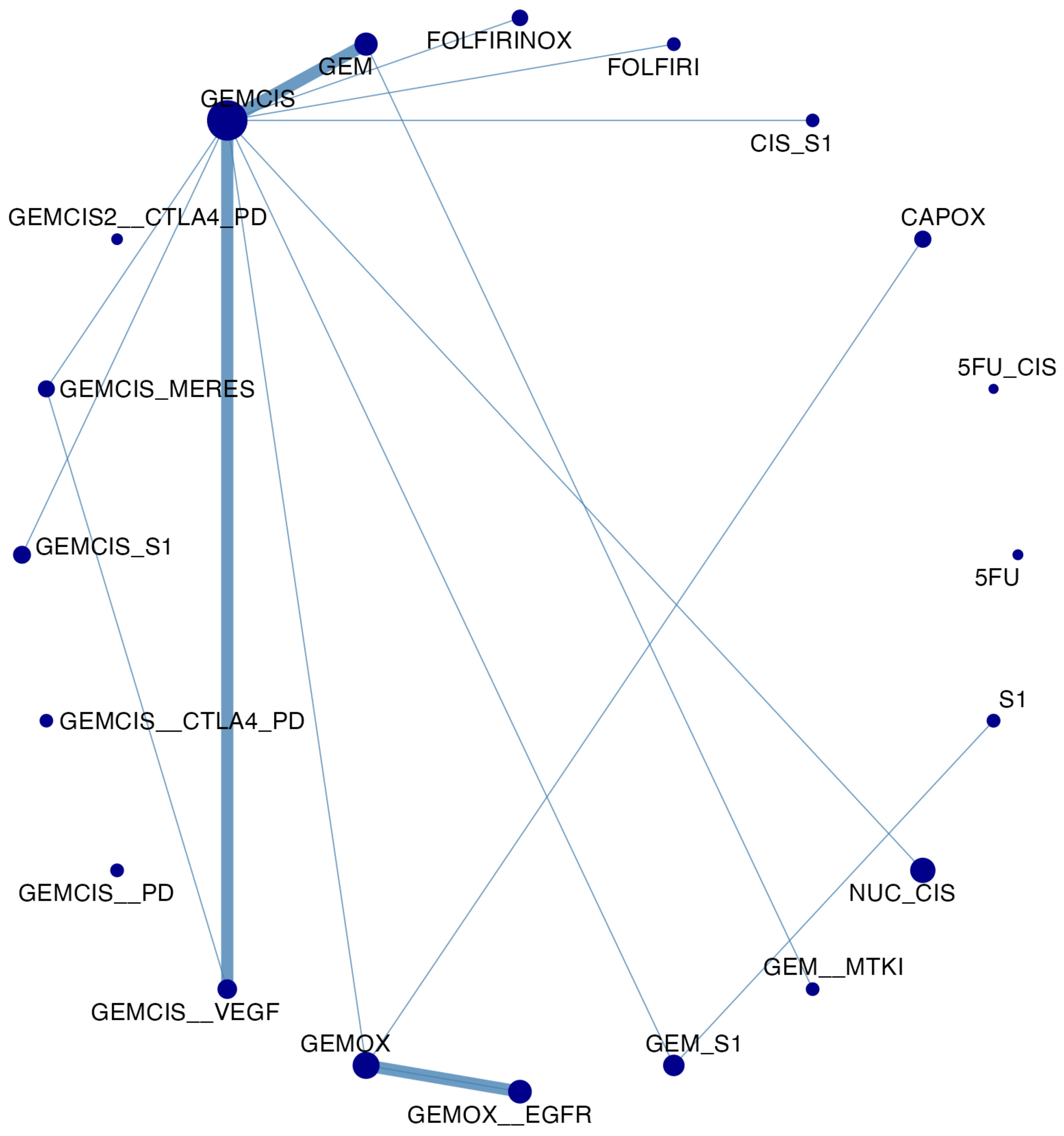

© 2026 Elemosho A et al. *JAMA Network Open*.

eTable 16. Diarrhea

Regimen	OR with CI	P-score Rank
Gemcitabine plus Oxaliplatin	4.390 (0.88–21.88)	1
Gemcitabine plus Oxaliplatin plus Panitumumab	4.286 (0.43–43.08)	2
Gemcitabine plus Oxaliplatin plus Cetuximab	2.781 (0.38–20.16)	3
Gemcitabine plus Cisplatin plus S-1	0.661 (0.11–4.03)	4

*P Score is Ranked Worst (1) to Best (5)

eFigure 34 Network Graph of Diarrhea

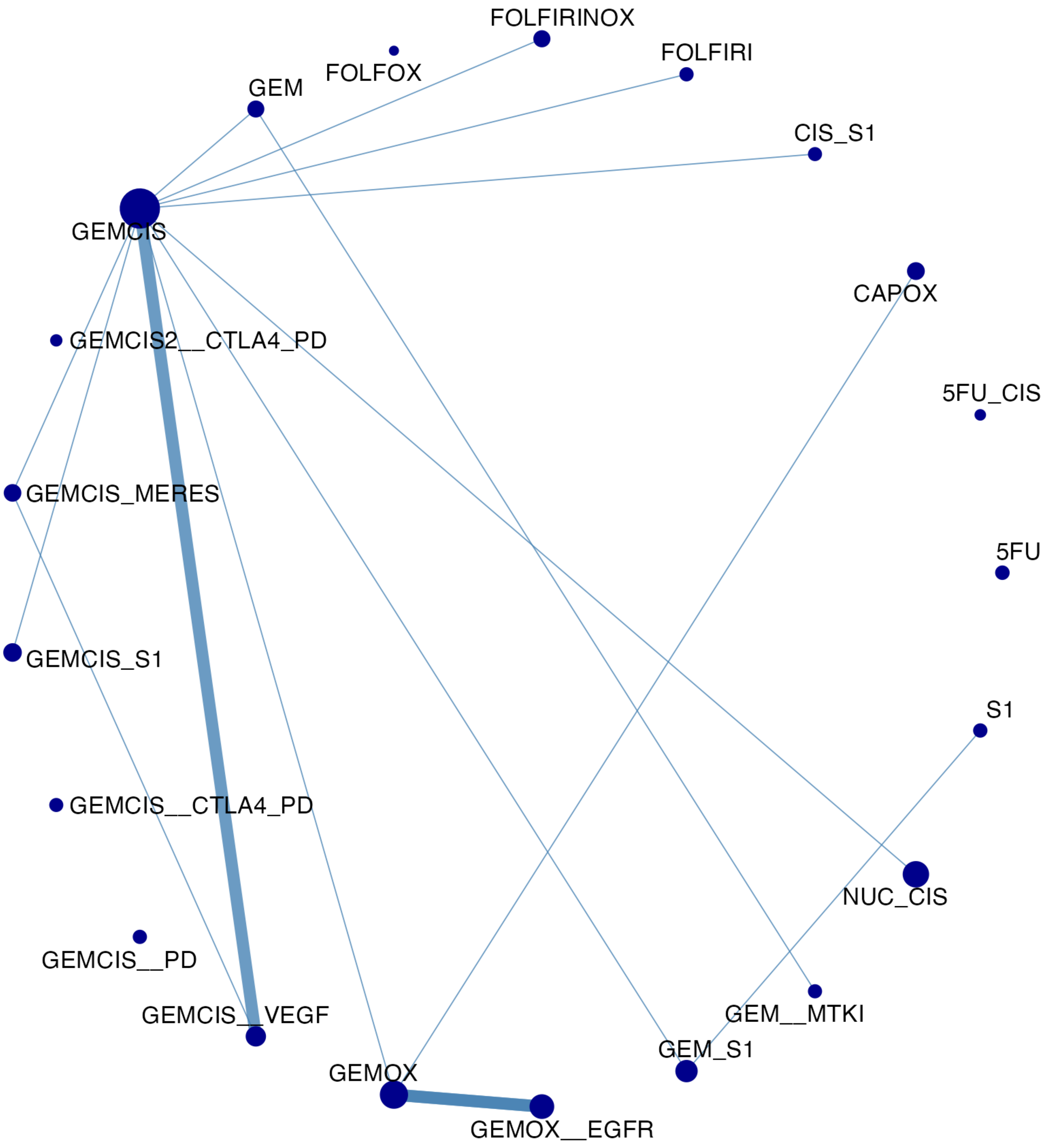

© 2026 Elemosho A et al. *JAMA Network Open*.

eTable 17. Fatigue

Regimen	OR with CI	P-score Rank
Gemcitabine plus Cisplatin plus Durvalumab plus Tremelimumab	56.590 (2.09–1534.76)	1
Gemcitabine plus Oxaliplatin plus Panitumumab	41.747 (3.26–535.40)	2
Gemcitabine plus Oxaliplatin	24.438 (3.09–193.01)	3
Gemcitabine plus Cisplatin plus Durvalumab	19.261 (9.58–38.73)	4
Gemcitabine plus Oxaliplatin plus Cetuximab	19.499 (2.11–179.89)	5
Gemcitabine plus Cisplatin plus S-1	1.539 (0.53–4.46)	6

*P Score is Ranked Worst (1) to Best (5)

eFigure 35 Network Graph of Fatigue

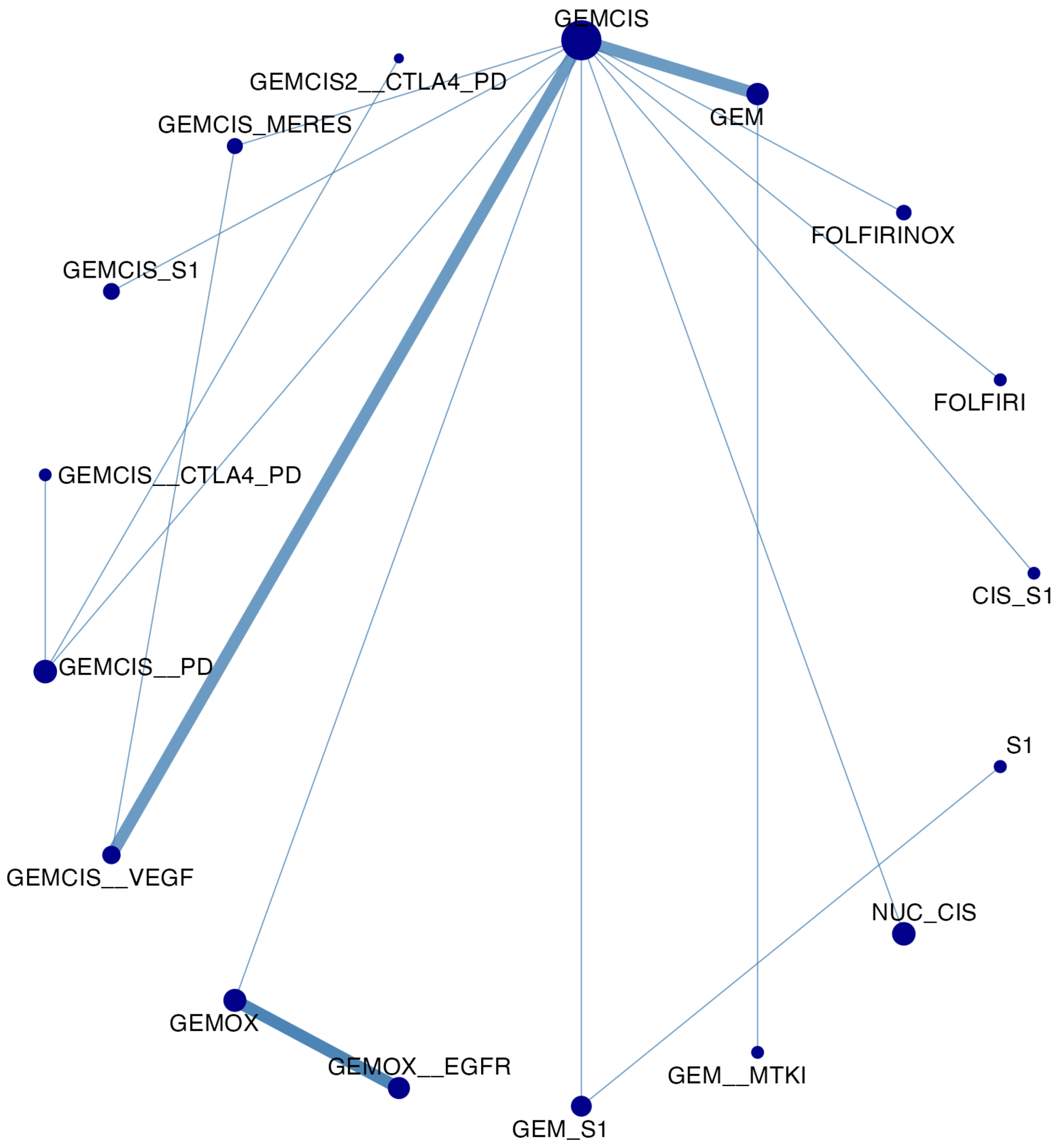

eTable 18. Sensory Neuropathy

Regimen	OR with CI	P-score Rank
Gemcitabine plus Oxaliplatin	0.957 (0.13–7.09)	1
Gemcitabine plus Oxaliplatin plus Panitumumab	0.935 (0.05–15.91)	2
Gemcitabine plus Oxaliplatin plus Cetuximab	0.644 (0.08–5.37)	3
Gemcitabine plus Cisplatin plus S-1	0.331 (0.01–8.20)	4

*P Score is Ranked Worst (1) to Best (5)

eFigure 36. Network Graph of Sensory Neuropathy

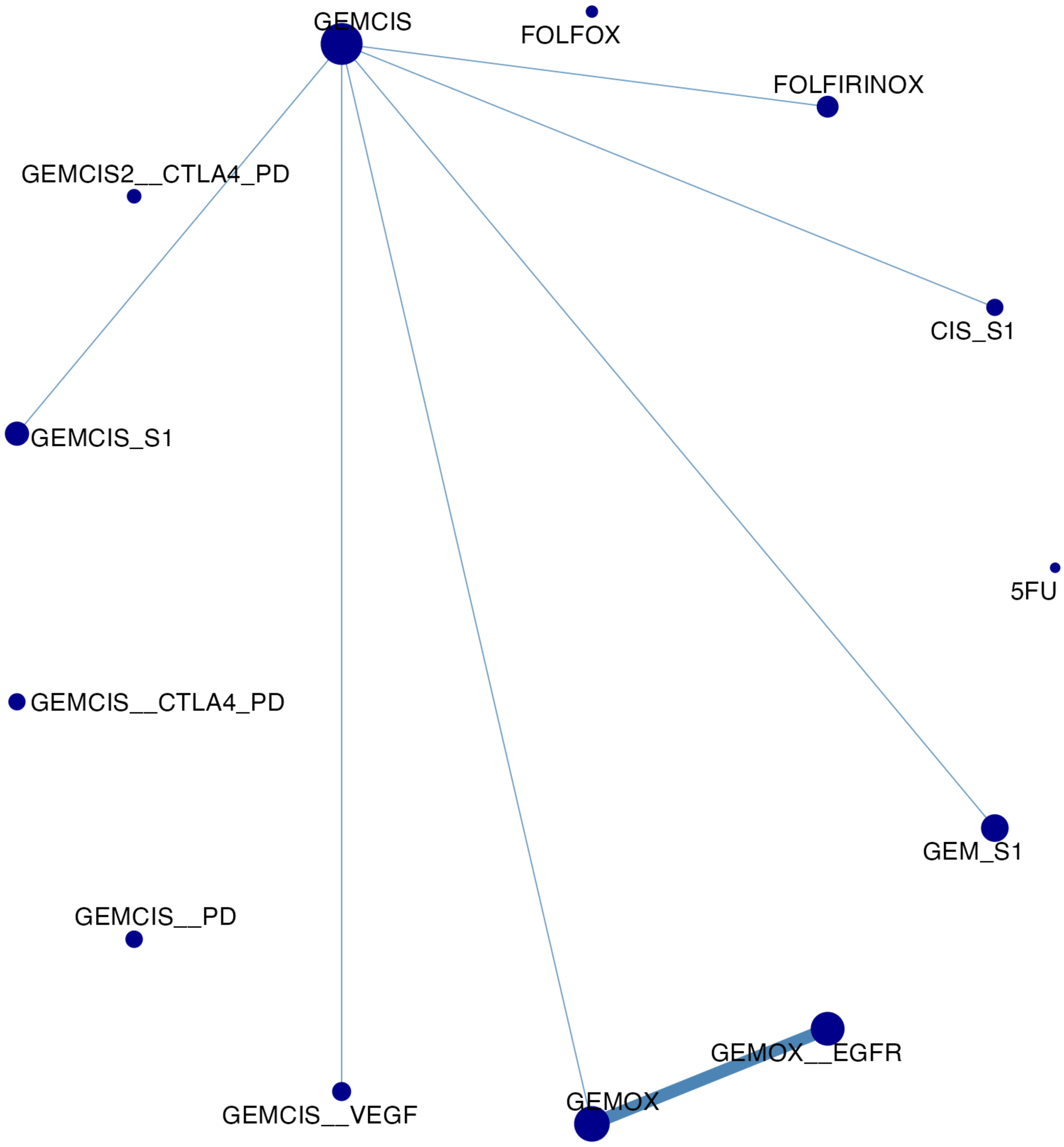

© 2026 Elemosho A et al. *JAMA Network Open*.

eFigure 37. Heat Map Showing the Posterior Probability (of All Regimen) That the Regimen in the Column is Superior to the Regimen in the Row for PFS, Defined as Pr[HR (Column vs Row) < 1]

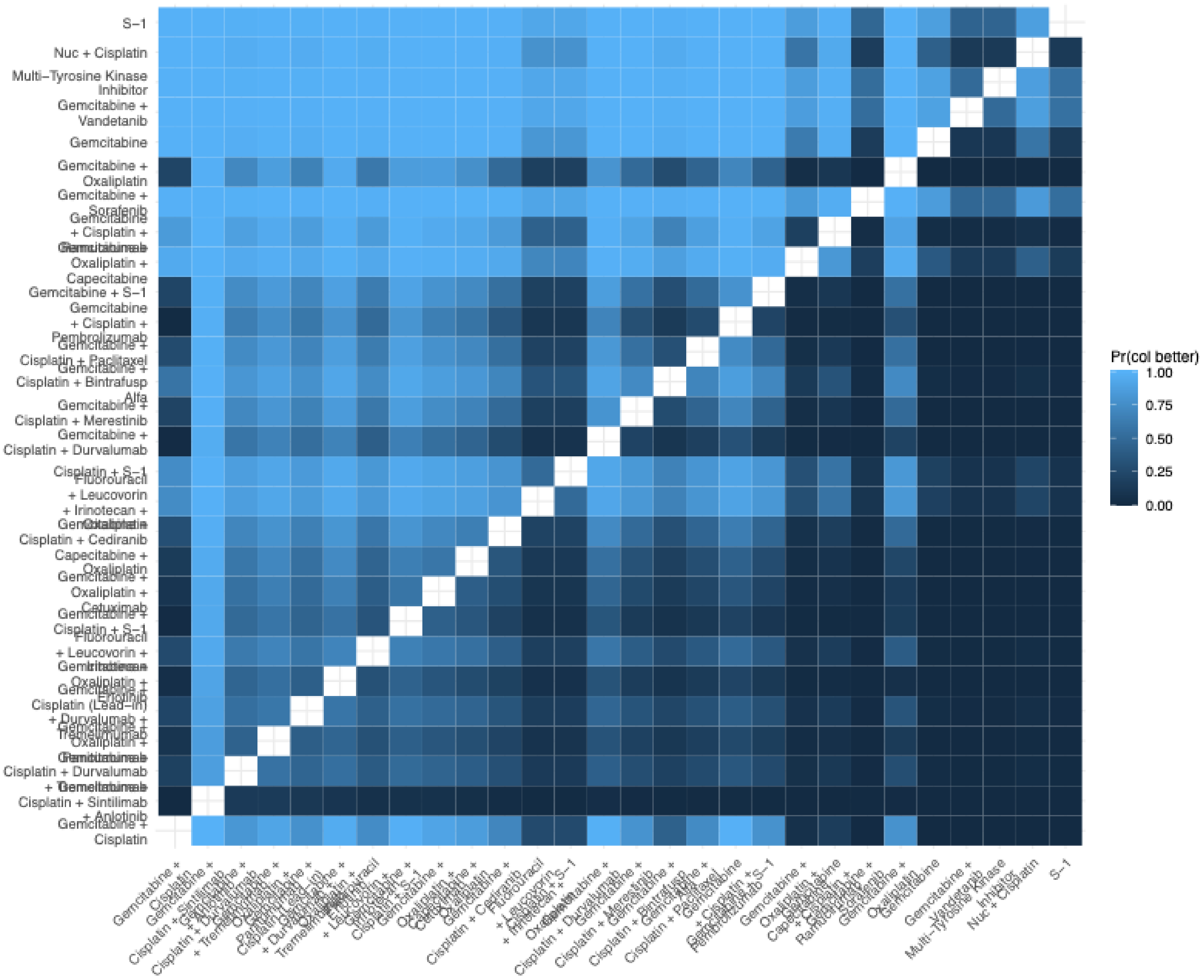

eFigure 38. Heat Map Showing the Posterior Probability (of All Regimen) That the Regimen in the Column is Superior to the Regimen in the Row for OS, Defined as Pr[HR (Column vs Row) < 1]

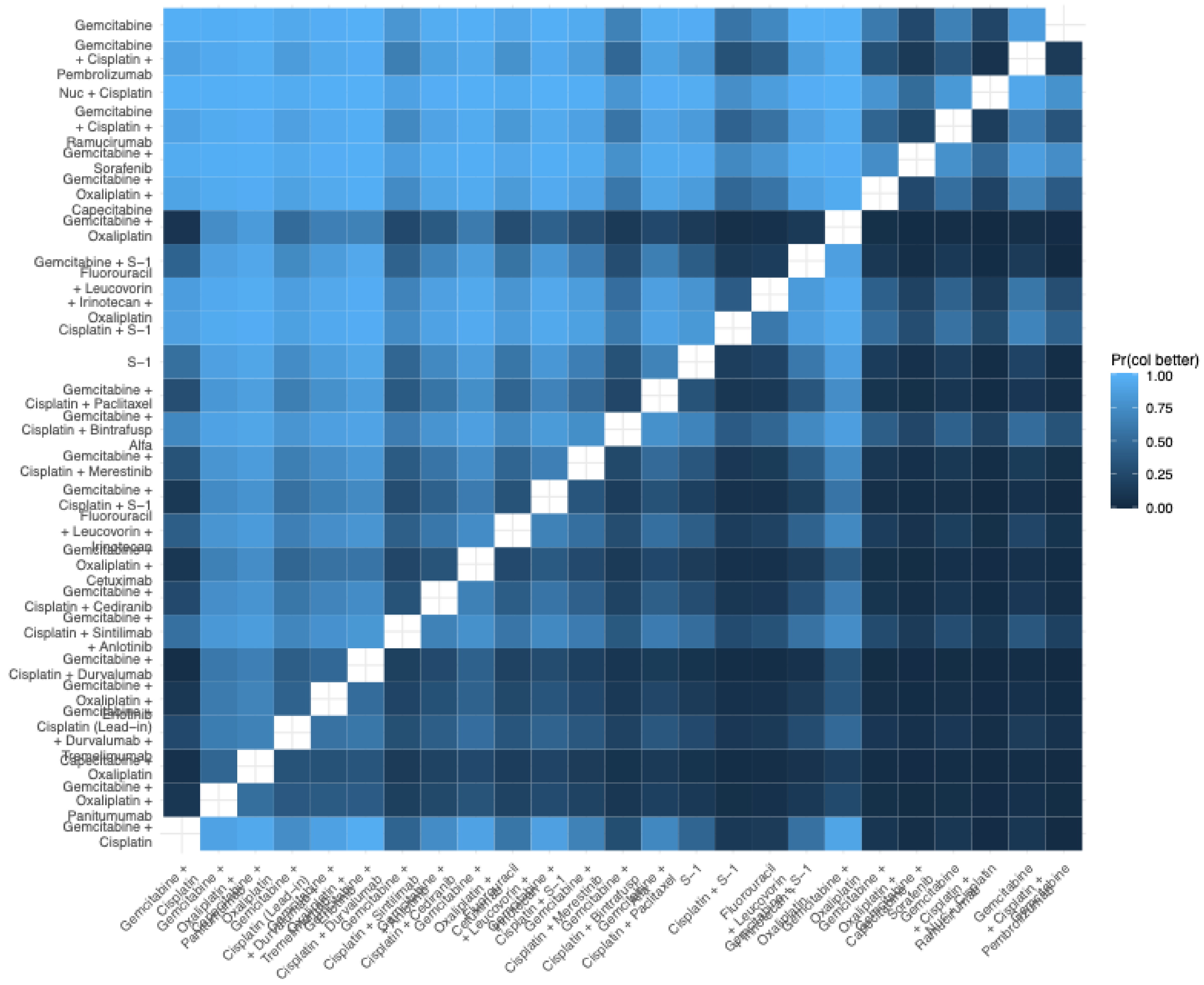

© 2026 Elemosho A et al. *JAMA Network Open*.
