# Supplementary material for: First-Line Chemotherapy Regimens for Unresectable Locally Advanced or Metastatic Biliary Tract Cancer: A Systematic Review and Bayesian Network Meta-Analysis
Source: JAMA Netw Open. 2026 Apr 15;9(4):e266849. doi: 10.1001/jamanetworkopen.2026.6849 (PMC13084461; doi:10.1001/jamanetworkopen.2026.6849)
Supplement: Supplement 2. — Data Sharing Statement [file jamanetwopen-e266849-s002.pdf]

Data Sharing Statement

Elemosho. First-Line Chemotherapy Regimens for Unresectable Locally Advanced or Metastatic Biliary Tract Cancer. *JAMA Netw Open*. Published April 14, 2026. doi:10.1001/jamanetworkopen.2026.6849

Data

Data available: No
